# High‐Fidelity Synthetic Data Replicates Clinical Prediction Performance in a Million‐Patient Diabetes Cohort

**DOI:** 10.1002/advs.202516196

**Published:** 2026-03-16

**Authors:** Francisco Ortuño, Víctor M. de la Oliva Roque, Javier‐Ignacio Ramirez‐Lopez, David P. Kreil, Joaquín Dopazo, Carlos Loucera

**Affiliations:** ^1^ Department of Computer Engineering, Automation and Robotics University of Granada Granada Spain; ^2^ Platform for Computational Medicine Andalusian Public Foundation Progress and Health‐FPS Seville Spain; ^3^ Institute of Biomedicine of Seville, IBiS University Hospital Virgen del Rocío/CSIC/University of Sevilla Sevilla Spain; ^4^ Institute of Molecular Biotechnology, Department of Biotechnology and Food Science BOKU University Vienna Austria; ^5^ Department of Computer Science and Artificial Intelligence Universidad de Sevilla Sevilla Spain

**Keywords:** algorithmic bias, biomedical plausibility, diabetes, generative deep learning, longitudinal patient data, privacy‐preserving research, synthetic electronic health records

## Abstract

Synthetic patient data offer a promising avenue for clinical research, but their usefulness depends on preserving statistical fidelity, biomedical plausibility, and patient privacy. To address this, a dual adversarial autoencoder is employed to generate longitudinal synthetic datasets from real‐world clinical data of nearly one million individuals with diabetes from the Andalusian Population Health Database. A multi‐faceted evaluation assesses data utility in a machine learning task, predicting chronic kidney disease onset, and evaluates the biomedical plausibility of generated disease trajectories. Models trained exclusively on synthetic data demonstrate predictive performance comparable to those trained on real data and show stability in feature importance rankings, indicating clinical coherence. However, bias and domain‐specific sex‐stratified analyses reveal inconsistencies not discernible through standard metrics, while data augmentation provides no performance benefit, as data saturation is reached given the large source population. These findings demonstrate that while synthetic data can replicate predictive performance, a robust validation framework combining machine learning utility with domain‐specific biomedical evaluation is essential. This work supports the use of synthetic data for large‐scale, privacy‐preserving research to enable a collaborative healthcare data ecosystem.

## Graphical abstract

This study generates high‐fidelity synthetic longitudinal records for a million‐patient diabetes cohort, successfully replicating clinical predictive performance. However, deeper analysis reveals algorithmic biases and trajectory inconsistencies that escape standard quality metrics. These findings challenge current validation norms, demonstrating why a robust, domain‐specific biomedical evaluation framework is essential for trustworthy privacy‐preserving healthcare research.

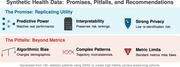

## 1 Introduction

The digitalization of hospitals and healthcare institutions has catalyzed an era where the healthcare system has become one of the most prolific producers of data [1]. This digital transformation has led to an exponential growth in the generation of diverse and extensive datasets, encompassing a myriad of patient information, clinical records, and health‐related variables. Currently, it has been estimated that more than 30% of existing data has been generated in a healthcare environment [2]. These rich sources of data have been instrumental in laying the foundation for the creation of new biomedical knowledge [3], enabling researchers to gain insights from real‐world patient experiences and outcomes, contributing to the expanding field of real‐world evidence (RWE) [4]. In the drive to implement secondary use of electronic health records (EHRs) while safeguarding sensitive medical data, healthcare institutions have employed deidentification techniques to create anonymized datasets [5]. However, de‐identification, while reducing privacy risks, cannot guarantee complete protection against reidentification attempts or linkage attacks that link patient data with external sources [5]. Different methods have been investigated to assess and minimize the risk of reidentification, particularly focusing on trajectory patterns of individual patients [6, 7, 8, 9]. Actually, patient trajectories, when observed sufficiently, retain a unique signature, heightening the risk of reidentification [10].

A more effective privacy assurance strategy involves synthesizing realistic data by learning from actual EHRs. Synthetic records, lacking a direct link to original patient records, prove highly effective in mitigating privacy attacks [11, 12]. Adopting differential privacy (DP) further ensures restricted disclosure of private information.

Generative adversarial networks (GANs) [13] have seen remarkable success in synthesizing Electronic Health Records (EHR) across various clinical applications [12, 14, 15]. Among the most widely used GAN‐based approaches for tabular data are Conditional tabular GANs (CTGAN) and Tabular Variational Autoencoders (TVAE) [16]. More recently, Denoising Diffusion Probabilistic Models (DDPMs) [17] have emerged as a more stable alternative, while Large Language Models (LLMs) [18, 19] excel at generating realistic EHR data. By leveraging rich medical knowledge from pretraining and capturing nuanced clinical semantics during fine‐tuning, LLMs produce contextually coherent records that can be tailored via prompting with minimal post‐processing.

Yet, despite these advancements, the core data sources within EHRs, namely the collections of set‐valued sequences describing patients' chronological medical conditions, have not received adequate attention [12, 14, 15]. This study focuses on the generation of realistic high‐dimensional discrete record sequences that encapsulate medical entities assigned to patients, such as diagnoses, age, sex, and visit dates. Previously, GAN or variational autoencoder (VAE) [20] architectures have been used, mainly applied to high‐dimensional continuous data like images [21]. However, training these models for high‐dimensional and discrete sequences, particularly sequences of patients' structured records, poses significant challenges [20]. The complexity arises due to nondifferentiable operations inherent in sampling words or EHRs [22, 23, 24]. Recently, dual adversarial autoencoder (DAAE) [25], a deep generative model tailored for sequences of set‐valued medical records, have been proposed to address these complexities. Unlike existing models that address either the continuous latent distribution or the discrete data distribution, DAAE adversarially learns both, enhancing the variety and realism of generated sequences while preserving global realistic features [26, 27, 28, 29, 30].

Most of the attempts of generating synthetic patient longitudinal data have used the MIMIC database [31], which contains sequential critical care measurements for more than 50 000 patients. However, critical care databases are designed to capture high‐density, acute care episodes. While invaluable for ICU‐specific tasks, these datasets are limited in their ability to model the long‐term disease trajectories characteristic of general population health.

In contrast, large administrative clinical databases offer a significantly wider temporal scope, encompassing diverse patient cohorts and a wealth of information that spans various medical specialties and healthcare settings, providing a more comprehensive and holistic view of real‐world clinical practices, patient outcomes, and disease trajectories. Specifically, Andalusia, the third largest region in Europe, with a population of 8.5 million inhabitants, holds the Population Health Database (BPS, Spanish acronym for Base Poblacional de Salud) [32], a large database where clinical data of patients has been cumulatively structured and consolidated over the last 20 years. Currently BPS stores detailed clinical data from more than 15 million of users of the Andalusian Public Health System, and one of its main objectives is to foster and provide support to clinical research. Such research can be carried out in Secure Processing Environments such as the Andalusian Platform for Generation of Medical Evidence (PAGEM) [33, 34].

In particular, BPS contains over 1 million EHRs of diabetic patients that were used to generate synthetic patient trajectories in this work. Unlike acute care databases, this cohort serves as a representative case study for modeling the longitudinal progression of chronic multimorbidity due to its systemic nature. These trajectories capture the sequential evolution of comorbidities toward diverse endpoints of varying severity, including renal failure, blindness, cardiovascular events, and exitus. Utilizing these patterns for early risk prediction is essential for future preventive medicine, facilitating interventions that improve patient quality of life while optimizing healthcare resource allocation [35].

In this study, we utilized a dual adversarial autoencoder to synthesize longitudinal datasets derived from near 1 million real‐world individuals with diabetes extracted from the Andalusian Population Health Database. We established a comprehensive evaluation framework to assess the data's utility in a machine learning task (predicting chronic kidney disease onset) and extended our analysis beyond standard metrics to elucidate the biomedical plausibility of the simulated disease trajectories.

More specifically, we aimed to: (i) measure the fidelity, structure and privacy of the generated records, (ii) investigate the potential algorithmic biases and domain‐specific inconsistencies of the generative process, which often escape detection through standard metrics, (iii) determine if models trained on the synthetic cohorts could achieve predictive performance comparable to those trained on real data, while also maintaining stability on the feature rankings, and (iv) replicate the process end‐to‐end to account for the stochastic nature of the generative and predictive models.

Ultimately, our objective is to demonstrate that a robust validation framework is essential for synthetic data generation. Therefore, by validating a reliable proxy of a large cohort of diabetic patients that minimizes re‐identification risks while faithfully retaining the richness of the original data, this work seeks to support the use of synthetic data for research by enabling a more collaborative healthcare data ecosystem, as evidenced by the diverse synthetic cohorts released in the international CAMDA conference [36,63].

## 2 Methods

### 2.1 Data Source

Real world longitudinal data was extracted from the BPS [32]. These datasets are derived from patient EHRs from the Andalusian public health system (i.e. all data from users of the Andalusian public health system). The Ethics Committee for the Coordination of Biomedical Research in Andalusia [37] granted approval for this study (Acta 08/19, 24/09/2019).

### 2.2 Design and Patient Selection

This study aimed to use a retrospective cohort encompassing all patients diagnosed with type 1 or type 2 diabetes mellitus (ICD10 codes E10 and E11) in Andalusia from 2003 to 2022. More specifically, only patients with correctly recorded gender and age at diabetes diagnosis were selected. Also, only patients diagnosed with diabetes at adult ages (≥ 18 years old) were kept. Other codes for 80 comorbidities including chronic diseases and other pathologies of interest were also considered (see Table S1A). The observation period for other comorbidities covers any previous and follow‐up diagnoses. Finally, demographic data like age and sex were coded assigning one label for each specific value (see Table S1B).

### 2.3 Secure Data Management

The data management procedure avoids any aspect that could compromise privacy by following the Andalusian Ministry of Health regulation for secondary use of medical data [38], as well as the Regulation of the European Health Data Space (EHDS) [39]. The data management procedure is as follows: (i) once the study is approved by the Data Access Committee the EHRs are requested to BPS, (ii) the BPS team extract the data and pseudonymize it, (iii) the BPS transfer the pseudonymized data to the PAGEM [34] Secure Processing Environment for the generation of real world evidence, (iv) the analysis of the data is carried out in the PAGEM, (v) once the study is finished the data is removed from the PAGEM infrastructure.

### 2.4 Patient Simulation Strategy

#### 2.4.1 Patient Data Encoding

Longitudinal patient data extracted from the BPS were coded as ordered sequences of visits to the health system that consist of the age of the patient at visit time along with the corresponding diagnoses. Thus, each patient is characterized by his/her sex and an ordered sequence of age‐diagnosis events, including a diabetes diagnosis and other prior or subsequent comorbidities or endpoints (e.g., retinopathy, renal failure) observed during the study period. Although simplistic, these patient trajectories contain a wealth of information about how comorbidities develop, leading up to a diabetes diagnosis and how patients may subsequently progress through additional conditions and symptoms toward endpoints of varying severity, including renal failure, blindness, cardiovascular complications, and, in some cases, death.

#### 2.4.2 Generative Adversarial Training

Dual adversarial autoencoders (DAAE) were used to learn sequences of set‐valued medical records [25]. Unlike existing models that address either the continuous latent distribution or the discrete data distribution, DAAE adversarially learns both, enhancing the variety and realism of generated sequences while preserving global realistic features [26, 27, 28, 29, 30]. Although other recent and promising generative architectures could also have been considered (e.g. transformers or diffusion models), adapting these approaches to discrete, set‐valued EHR datasets still requires complex positional and semantic modelling showing a limited performance. Consequently, as previously mentioned, these models have been more extensively applied to continuous longitudinal clinical data with a more short, regular and controlled structure (e.g., critical data in MIMIC [31]). In contrast, the DAAE provides stable adversarial training and efficient handling of irregular temporal patterns and multi‐label visit structures, making it a practical choice for our dataset and computational constraints.

This model was trained on an NVIDIA Tesla V100 GPU with 32 GB of VRAM. It was configured with a batch size of 256, L2 regularization, and a Gated Recurrent Unit (GRU) layer for the sequence‐to‐sequence autoencoder. The optimizer of the generative model was a differentially private stochastic gradient descent (DP‐Adam), which has been shown to guarantee privacy without leakage [25]. Also, no early‐stopping strategy was implemented, in accordance with the original implementation [25]. Instead, we adopted a fixed‐epoch approach of 500 epochs. This fixed‐epoch strategy does not degrade the model and serves a dual purpose: (i) reducing privacy leakage through DP‐Adam optimizer and (ii) ensuring robustness and consistency across replicates, avoiding suboptimal stopping due to transient noise in validation loss. Thus, this solution provides a controlled and reproducible framework for convergence.

#### 2.4.3 Synthetic Preprocessing

For each generative run, a synthetic cohort of 800 000 individuals was generated using dual adversarial autoencoders (DAAE), based on the real training dataset. This synthetic dataset was reviewed and preprocessed to retain only high‐quality samples. Initially, individuals without a diabetes diagnosis or associated sex were excluded, and visits that were either empty or contained only age labels were removed. Additionally, only the first occurrence per patient was retained for chronic disease diagnoses. Regarding age, when multiple age labels were present within a single visit, only the highest value was kept. On the other hand, if a visit lacked an age label, age was inferred using the average age of the preceding and subsequent visit. The set of samples resulting from this preprocessing pipeline constitutes what we term the *refined synthetic cohort*.

### 2.5 Model Evaluation

To validate our model, we split the real dataset into an 80% training set and a 20% test set. We assessed the model's robustness in two ways. First, for the **Single Split, Multiple Replica (SSMR)** analysis, we generated five synthetic cohorts from the same data split using different random seeds to evaluate how the model's inherent stochasticity affects the results. Second, for the **Multiple Split, Single Replica (MSSR)** analysis, we created five distinct training/test splits and generated one synthetic cohort for each, which helped us assess the model's independence from the specific training data used.

#### 2.5.1 Metrics for Evaluating the Generative Modeling Robustness

To rigorously assess the quality, fidelity, and utility of the generated synthetic cohort, we employed a multi‐faceted evaluation strategy.

The first category comprises metrics that directly assess the statistical properties and structure of the generated dataset itself. These metrics evaluate the synthetic data's fidelity to the original data's distributions and correlations, as well as its ability to generate the same structure, like data ranges, variable types, etc.

This second category comprises metrics designed to quantify the risk of re‐identifying real individuals or disclosing their sensitive attributes from the generated dataset. The evaluation focuses on ensuring the model does not memorize or leak private information from the training data, thereby protecting the privacy of the original cohort's participants.

The third category evaluates the practical, real‐world utility of the synthetic data by measuring the performance of predictive machine learning models. This involves training models on various dataset (real data only, synthetic data only, and a combination of both) and testing them against a held‐out set of real data. This *Train‐Synthetic, Test‐Real* (TSTR) paradigm serves as a benchmark for determining whether the synthetic data retains the complex patterns and relationships necessary to build effective predictive tools.

#### 2.5.2 Metrics to Assess the Fidelity and Quality of the Synthetic Cohort

This set of metrics have been implemented using the *SDMetrics* Python package (v0.21.0 ‐ 2025‐05‐29). We provide two distinct scoring systems: one to properly assess the generative model's performance on the raw dataset, and another set of metrics that analyze the synthetic cohort's quality with respect to its intended use (i.e., after pre‐processing). From now on, the samples resulting from applying the pre‐processing pipeline are referred to as the *refined synthetic cohort* for easy identification.

***Boundary Adherence Score (BAS)***: This metric evaluates whether numerical and datetime columns in the synthetic data respect the minimum and maximum boundaries established by the real data. It calculates the percentage of synthetic rows where values fall within the observed range of the original data. Missing values are considered valid only if they were also present in the real data. A score of 1.0 indicates that all synthetic values are within the real data's boundaries, signifying perfect adherence.

***Category Adherence Score (CAS)***: This metric verifies that categorical and boolean columns in the synthetic data only use categories present in the original data. It computes the proportion of synthetic data points that belong to the set of categories observed in the real data. Missing values are treated as a valid category if they existed in the original dataset. A score of 1.0 indicates perfect adherence, meaning the model did not invent any new categories.

***Kolmogorov–Smirnov Complement Score (KSS)***: This metric compares the distributions of continuous (numerical or datetime) columns between the real and synthetic data using the two‐sample Kolmogorov–Smirnov test. For each column, it measures the maximum difference between the cumulative distribution functions of the real and synthetic data. The final score is the average of 1 ‐ (max difference) across all continuous columns. A score of 1.0 indicates that the distributions are identical, while lower scores signify divergence.

***Correlation Similarity Score (CrSS)***: This metric assesses how well the synthetic data preserves the pairwise correlations between numerical columns found in the real data. It computes the Pearson's correlation matrix for both datasets and calculates the absolute difference between these two matrices. The final score is 1 minus the average of these differences. A score of 1.0 indicates that the correlational structure has been perfectly replicated, while lower scores suggest the model failed to capture the linear relationships between variables.

***Contingency Similarity Score (CtSS)***: This metric evaluates how well the synthetic data captures the association between pairs of discrete columns. It computes a contingency‐based measure of association (Cramer's V) for all pairs of categorical columns in both the real and synthetic datasets. The final score is 1 minus the average absolute difference between these association values. A score of 1.0 means the associations between categorical variables are perfectly replicated. When pairing a numerical column with a categorical column it first discretizes into bins the numerical column.

***Total Variation (TV) Complement Score (TVS)***: This metric compares the probability distributions of discrete (categorical or boolean) columns between the real and synthetic data. For each column, it calculates the Total Variation Distance (TVD), which is half the sum of the absolute differences of the category frequencies. The final score is the average of 1 ‐ TVD across all discrete columns. A score of 1.0 indicates identical distributions, while lower scores signify a divergence in category probabilities.

***Sliced Wasserstein Distance (SWD)***: This metric evaluates the multivariate distributional alignment between the synthetic data and real data beyond pairwise correlations. Features were normalized to [0, 1] prior to computing the metric using the average of the 1D Wasserstein distances along 1,000 random directions [40].

***Co‐ocurrence correlation (CoC)***: This custom‐designed metric compares the co‐occurrence frequencies of comorbidities between real and synthetic cohorts while preserving their temporal order of appearance. Specifically, for each pair of comorbidities, CoC measures how often one condition occurs after another across patient trajectories and computes the Pearson's correlation between real and synthetic co‐occurrence frequencies.

#### 2.5.3 Metrics to Assess the Risk of Re‐identifying Patients

This set of metrics have been implemented using the SDMetrics Python package (v0.21.0 ‐ 2025‐05‐29), except membership inference attack that was implemented with Synth–MIA [41]. The threat model assumes the attacker has access to the sex and age at diagnosis for each record, whereas the MIA is conducted under a zero‐knowledge assumption.

***Disclosure Protection Score (DPS)***: This metric directly quantifies the risk of re‐identification by simulating a worst‐case scenario attack on the entire dataset. It assumes an adversary knows a specific set of attributes for individuals and attempts to infer a sensitive attribute. The metric calculates how much the synthetic data helps the adversary improve their guesses compared to a baseline (e.g., simply guessing the most common category). The resulting score measures the data's safety, where a higher score indicates a lower risk of sensitive information being disclosed.

***Disclosure Protection Estimate Score (DPeS)***: This metric estimates the DPS by running the same simulated attack on multiple, random subsamples of the data and averaging the results. This approach is more computationally efficient for very large datasets. The final score represents the data's safety relative to a baseline of random guessing, where a score of 1.0 indicates that the synthetic data offers disclosure protection equivalent to completely random data, and 0.0 suggests a high risk of disclosure.

***New Row Synthesis (NRS)***: This metric is designed to quantify the novelty of the generated data. Its primary purpose is to assess whether the model is genuinely creating new data points or simply replicating rows from the original training dataset. For each row in the synthetic dataset, the metric identifies the most similar row in the real dataset and calculates a novelty score. The final score is the complement of the fraction of matching rows, where a synthetic row is said to match a real row if the values are identical (categorical variables), within a tolerance for numeric or date‐based variables, or the missing values are on identical positions. The threshold for continuous variables is set to 0.01 following the suggested values of SDMetrics.

***Membership Inference Attack (MIA)***: This metric evaluates the risk of re‐identification by simulating a scenario where an adversary attempts to determine if a specific patient record was used to train the model. We measured the success of this attack using the Area Under the Receiver Operating Characteristic Curve (AUC). An AUC of 0.5 indicates that the attacker performs no better than random guessing, which confirms that the model has not memorized unique training samples. Conversely, an AUC significantly higher than 0.5 would indicate potential data leakage and compromised privacy. It is important to note that the attacker's score can be arbitrarily oriented, AUROC values below 0.5 indicate an inverted ranking (anti‐correlation) rather than improved leakage. For interpretation, when necessary, we report the orientation‐invariant measure ${\mathrm{AUROC}}^{*}=\max\left(\mathrm{AUROC},1-\mathrm{AUROC}\right)$ and the absolute advantage over random Δ=|AUROC−0.5|.

#### 2.5.4 Algorithmic Bias Analysis

First, we assessed potential sex bias in the synthetic cohorts by comparing real and synthetic data within sex and across replicas, focusing on prevalence, distributional fidelity, and the portability of sex‐specific signals. For each disease (feature) we computed (i) prevalence by sex in real and synthetic data and the female–male difference (pp); (ii) *KS* statistic by sex comparing the synthetic vs. real distributions (summarized as mean and max *KS* across runs); (iii) *Sex Bias Delta*, defined as the percentage‐point difference in female representation between synthetic and real cohorts; (iv) feature amplification, the correlation across runs between a feature's sex‐specific prevalence shift [(synthetic ‐ real in women) ‐ (synthetic ‐ real in men)] and *Sex Bias Delta*; and (v) sex predictability (AUROC) using logistic regression in two transfer settings: Real to Synthetic and Synthetic to Real.

Then, we quantitatively analyzed the impact of comorbidity prevalence on the quality of the synthetic data generation. Specifically, we studied how relative prevalence errors between the synthetic and real cohorts vary depending on the original comorbidity prevalence. We aim to identify an empirical prevalence threshold below which comorbidities may exhibit considerable error and should be generated with caution.

#### 2.5.5 Sex‐Driven Comorbidities Patterns

To compare the incidence of diagnoses between sexes across different age groups, a stratified analysis was performed, partitioned by replica status (real world and simulated sets). For each replica, the rate of diagnosis per person was calculated for both males and females within 5‐year age intervals. This was achieved by counting the total number of diagnoses within each age bin and normalizing this count by the total number of individuals of the corresponding sex in that replica. The primary metric for comparison was the absolute difference between the female and male diagnosis rates. A 95% confidence interval was computed for this difference to assess statistical significance using Wald's approximation.

To quantitatively assess the similarity between the trajectory from the original data and those from each synthetic replica, we used Dynamic Time Warping (DTW) [42]. DTW is a well‐established algorithm for measuring the similarity between two temporal sequences, which is robust to shifts or delays in the time axis. This makes it particularly suitable for evaluating if a synthetic replica captures the overall shape of a biomedical pattern, even if the pattern is slightly delayed. We used the *fastdtw* Python library (v 0.3.4) with a standard Euclidean distance to calculate the dissimilarity score between the original trajectory and each synthetic replica's trajectory. A lower DTW distance indicates a higher degree of similarity between the two trajectories.

#### 2.5.6 Static Predictive Performance in Real World Scenarios

The endpoint predictive analysis has been carried out using *scikit‐learn* Python library (v1.2.1), whereas the model fitting has been made using the *interpret* Python library (v0.3.0).

To evaluate the practical, real‐world utility of the synthetic data, we measured the performance of machine learning models in a clinically relevant task: predicting the onset of chronic kidney disease (CKD) in patients post‐diabetes diagnosis. To ensure clinical realism, individuals with non‐ascending ages and those with an end‐point before diabetes were removed and the models were restricted to using only the patient history available up to the time of the diabetes diagnosis. Our evaluation was structured around three distinct training scenarios:

1. **Real Data Model**: An Explainable Boosting Machine (EBM) [43] was trained and validated using only real patient data from the Andalusian Population Health Database (BPS).
2. **Synthetic Data Model**: An identical EBM was trained exclusively on the refined synthetic cohort.
3. **Hybrid Data Model**: A third EBM was trained on a combined dataset of both real and refined synthetic data to assess the potential for data augmentation to improve performance.

The performance of these three models was then evaluated on a held‐out test set composed entirely of real patient data. This approach ensures that the evaluation directly measures how well models trained on synthetic data generalize to real‐world clinical situations. In addition we followed a Cross Validation Strategy (CVS) to ensure the models were not just performant but also stable, by performing ten repeated 10‐fold cross‐validations over the training set, while evaluating the fitted models on the held‐out set.

***Predictive Performance (AUROC)***: The primary metric for model performance was the Area Under the Receiver Operating Characteristic curve (AUROC). This evaluates the model's ability to correctly distinguish between patients who will develop the endpoint and those who will not. We compared the AUROC scores achieved on the test set by the three models (Real, Synthetic, and Hybrid) to quantify the performance gap, if any, between them. In all cases we report mean and standard deviation of the CVS model fits. Furthermore, to account for variations in data quality over time, we also report the AUROC disaggregated by the calendar year of diabetes diagnosis.

***Hyperbolic Weighted Tau (HWT)***: This score is a rank correlation coefficient that measures the similarity between two ordered lists. Unlike the standard Kendall's tau, the weighted version does not treat all disagreements in rank equally. Specifically, hyperbolic weighting [44] penalizes disagreements at the top of the list more heavily than those at the bottom. The score goes from ‐1 (reversed ranks) to 1 (perfect agreement), where 0 indicates no association between the rank systems. We compared the HWT across all the CVS learned ranks for the three models (Real, Synthetic, and Hybrid) and between the ranks learned by the bootstrapped models fitted on real data vs. the ranks learned on the CVS models fitted in the synthetic data.

***Prediction Distribution Stability (PDS)***: We assessed model stability by analyzing the variance in patient‐wise prediction probabilities on the test set. Specifically, we compared predictions from models (Real, Synthetic, and Hybrid) trained on the cross‐validation splits (CVS) to those from the model trained on the full dataset.

#### 2.5.7 Trajectory‐Based Predictive Performance in Real World Scenarios

A similar approach was performed to evaluate the predictive potential of trajectories in synthetic data. Specifically, the appearance of chronic kidney disease (CKD) post‐diabetes diagnosis was predicted based exclusively on their previous ordered clinical records. A recurrent neural network based on a Long Short‐Term Memory (LSTM) architecture was applied to model temporal dependencies in patient histories. A hyperparameter tuning was performed with a training‐validation split strategy (70/30). More details are provided in Supporting Information. Except for the Hybrid Data Model scenario (given no interesting results were previously provided in a simpler model), the same two distinct training environments were proposed: Real Data Model vs. Synthetic Data Model. As previously, the AUROC was used as the primary metric for model performance, comparing Real vs. Synthetic datasets to quantify the performance gap, if any.

## 3 Results

### 3.1 Patient Data

An initial cohort of 1 062 550 patients was considered for this study. From this group, only 908 673 individuals with correctly coded gender information and a diagnosis of diabetes received in adulthood (age >18 years) between 2003 and 2022 were included in the analysis. The average age at diabetes diagnosis was 60.56 ± 13.83 years. Comorbidities lacking an accurately recorded visit date were also excluded from the analysis. Therefore, the patients selected have a total of 5 869 024 pathologies diagnosed in 5 052 059 medical visits between 1930 and 2022.

### 3.2 Synthetic Generation

For each generative run, a synthetic cohort of 800 000 individuals was generated using dual adversarial autoencoders (DAAE) [25], based on the real training dataset. Generative training was computationally intensive, with each synthetic replica requiring approximately 5191 ± 81 min (85–89 h), considering both multiple replicas and splits. Loss curves for the five generations in both Single Split ‐ Multiple Replica (SSMR) and Multiple Split ‐ Single Replica (MSSR) analyses are provided in Figures S1 and S7, respectively. Although these curves indicate stabilization at 100–150 epochs, minimum values were always found after at least 400 epochs, thus reinforcing the selection of a fixed‐epoch strategy.

This synthetic dataset was then reviewed and preprocessed to retain only high‐quality samples. Across all synthetic dataset replicates and splits, an average of 12.38 ± 2.15 percent of synthetic individuals were discarded during preprocessing steps. Namely, 7.76 ± 1.99% of synthetic individuals did not have an associated diabetes diagnosis, 1.85 ± 1.16% had no associated sex, and finally. Resulting from this preprocessing, a refined synthetic cohort of an average of 702.604 ± 13.330 individuals was selected for further analysis for each run. Static predictors are further refined by removing 1.34 ± 0.80% of individuals with non‐ascending diagnosis and 1.43 ± 0.58 % of those with the end‐point diagnosis before their diabetes diagnosis ages. A breakdown of discarded synthetic individuals across all replicates and splits can be found in Table S15.

### 3.3 Synthetic Quality and Privacy Metrics

To provide a robust and transparent assessment of our model's performance despite the limited number of replicates, we report the results for each quality metric as the median value across these five runs, accompanied by the range (minimum and maximum values).

The quality was assessed on both the raw generative model output and on the *refined* cohort (i.e., after applying the pre‐processing pipeline for the prediction task) for the Single Split ‐ Multiple Replica (SSMR) analysis. The results are summarized in Table 1.

**TABLE 1:** Data Fidelity and Privacy Metrics. Scores are presented as Median (Min ‐ Max) across all runs for both the “raw” and “refined” cohorts for the Multiple Splits ‐ Single Replica (MSSR) and Single Split ‐ Multiple Replica (SSMR) analysis. Distribution & Structure (Higher is Better, Max 1.0): KSComplement and TVComplement measure the similarity of continuous and discrete distributions, respectively. BoundaryAdherence and CategoryAdherence assess the preservation of data limits and labels. CorrelationSimilarity, ContingencySimilarity, and CoocurrenceCorrelation evaluate the retention of two‐way relationships. Distance Metrics (Lower is Better, min 0): Sliced Wasserstein measures multivariate distance; DTW measures temporal distortion in diagnosis trajectories. Privacy & Novelty: MIA AUROC estimates re‐identification risk (ideal value 0.5); DPeS indicates disclosure protection (Higher is better, max 1); NRS (New Row Synthesis) measures the generation of unique, non‐memorized records (Higher is better, max 1).

		MSSR		SSMR	
Kind	Metric	(Raw)	(Refined)	(Raw)	(Refined)
Distribution& Structure	Contingency Sim.	0.95 [0.54, 1.00]	0.98 [0.72, 1.00]	0.95 [0.54, 1.00]	0.98 [0.73, 1.00]
	Correlation Sim.	0.97 [0.00, 1.00]	0.97 [0.00, 1.00]	0.97 [0.00, 1.00]	0.97 [0.00, 1.00]
	KS Complement	0.90 [0.38, 0.99]	0.87 [0.38, 0.97]	0.90 [0.38, 0.99]	0.86 [0.40, 0.97]
	TV Complement	1.00 [0.75, 1.00]	0.99 [0.87, 1.00]	0.98 [0.72, 1.00]	0.99 [0.87, 1.00]
	Boundary Adh.	1.00 [0.75, 1.00]	1.00 [0.86, 1.00]	1.00 [0.75, 1.00]	1.00 [0.86, 1.00]
	Category Adh.	1.00 [0.75, 1.00]	1.00 [1.00, 1.00]	1.00 [0.75, 1.00]	1.00 [1.00, 1.00]
	Co‐occurrence Cor.	0.91 [0.90, 0.91]	0.94 [0.93, 0.94]	0.90 [0.87, 0.91]	0.94 [0.91, 0.95]
Distance	Sliced Wasserstein	0.03 [0.02, 0.03]	0.01 [0.01, 0.02]	0.03 [0.03, 0.04]	0.02 [0.01, 0.03]
	DTW Distance	0.50 [0.22, 0.59]	0.48 [0.31, 0.57]	0.39 [0.21, 0.42]	0.37 [0.25, 0.64]
Privacy & Novelty	NRS	0.81 [0.79, 0.82]	0.70 [0.70, 0.74]	0.81 [0.78, 0.82]	0.70 [0.67, 0.73]
	DPeS	0.50 [0.49, 0.50]	0.55 [0.52, 0.55]	0.49 [0.48, 0.51]	0.54 [0.50, 0.55]
	MIA AUROC	0.50 [0.49, 0.51]	0.39 [0.39, 0.40]	0.50 [0.49, 0.50]	0.39 [0.39, 0.40]

It is important to note that similar results can be obtained from the raw and refined synthetic cohorts for both analyses: the Single Split ‐ Multiple Replica (SSMR) and Multiple Split ‐ Single Replica (MSSR). This indicates the metrics are not significantly influenced by either the patient splits used for training (MSSR) or the inherent stochastic processes of the generative methods (SSMR). A descriptive analysis for both the **SSMR** and **MSSR** cohorts, mirroring the analysis presented in this manuscript, is provided in the Figures S2–S5 (SSMR raw cohort) Figures S8–S12 (MSSR raw cohort), and Figures S13–S16 (MSSR refined cohort).

The metrics reported in Table 1 confirm a high degree of statistical fidelity for both the **SSMR** and **MSSR** raw cohorts, which is preserved after applying the pre‐processing pipeline to create the refined cohorts. The median scores for most metrics are excellent, often exceeding 0.95, indicating that, on average, the synthetic data successfully captures the structural properties, univariate distributions, and pairwise trends of the original data. Moreover, the synthetic data exhibits multidimensional fidelity with raw synthetic cohorts exhibiting a low mean transport cost (Median SWD: 0.034–0.036), which improved in the refined cohorts (Median SWD: 0.0175–0.0184), suggesting a strong geometric alignment. Also, the Co‐occurrence Correlation (CoC) metric showed high median values across all cohorts (0.90–0.94), indicating strong agreement between real and synthetic temporally ordered comorbidity co‐occurrences. Interestingly, slightly higher CoC values were observed for the refined cohorts, suggesting an improved preservation of temporal comorbidity relationships with the preprocessing.

Regarding privacy, the empirical disclosure protection estimate score (DPeS) maintained robust median values (0.48–0.55) across all permutations. Most importantly, the raw Membership Inference Attack (MIA) AUROC perfectly mirrored a random‐guessing baseline with scores close to 0.5 across both SSMR and MSSR evaluations. While the refined cohorts yielded AUROC bounds below 0.50, this artifact represents an inverted attacker score orientation, which after correction confirms no meaningful membership signals were leaked (effective ${\mathrm{AUROC}}^{*}*$ ≈ 0.60, Δ ≈ 0.1). Similarly, the New Row Synthesis (NRS) metric confirmed that the model generated novel records rather than memorizing the training data, achieving a median of 0.81 for the raw cohorts and 0.70 for the refined cohorts.

However, the wide range for some metrics, particularly Correlation Similarity (0.00–1.00), highlights variability across the independent replicas irrespectively of the evaluation strategy. A deeper analysis revealed that these low scores were consistently associated with pairs of comorbidities with very low prevalence. The drop in score for these pairs was primarily due to the generative model producing a correlation with the opposite sign to that observed in the real data, a phenomenon more likely to occur when relationships are weak and data is sparse. To further understand the structural origins of these inconsistencies, we will next focus on a detailed analysis of algorithmic bias.

### 3.4 Per‐Replica Performance Diagnostics

To further characterize the generative process, we performed a per‐replica diagnostic to identify performance trade‐offs in the refined cohorts. As shown in Table 2, no single replica dominated within a single split (SSMR, split 1) or across all runs (global: SSMR + MSSR). See Supporting Information metrics.xlsx for the full‐range per‐replica results (raw and refined).

**TABLE 2:** Performance comparison of synthetic data replicas across single‐split (SSMR) and global (SSMR + MSSR) evaluation scopes. The table reports the best and worst performing runs for representative statistical and clinical metrics, alongside the scores of the selected replica (s1r0) used for downstream analysis. Best performing scores for each category are bolded; arrows indicate optimization direction ((↑) maximize, (↓) minimize).

		Best Run	Worst Run	Selected Run
Scope	Metric	(Score)	(Score)	(s1r0) Score
SSMR	Contingency Sim. (↑)	s1r4 (0.971)	s1r1 (0.961)	0.970
	DTW Distance (↓)	**s1r0 (0.251)**	s1r4 (0.641)	0.251
	Sliced Wasserstein (↓)	**s1r0 (0.015)**	s1r1 (0.023)	0.015
Global	KS Complement (↑)	s5r0 (0.860)	s3r0 (0.814)	0.838
	DTW Distance (↓)	**s1r0 (0.251)**	s1r4 (0.641)	0.251

On the one hand, Replica 4 achieved the highest scores for Contingency Similarity (0.97) in the within‐split (SSMR) analysis, suggesting excellent preservation of variable correlations. However, this same replica yielded the poorest temporal fidelity (DTW: 0.64). Conversely, Replica 0 achieved superior temporal alignment (DTW: 0.25) with only a marginal drop in statistical scores, highlighting a selection trade‐off.

On the other hand, when considering all generated instances, the divergence persists. The replica exhibiting the highest statistical fidelity was Run s5r0 (split 5, replica 0), achieving the highest KS Complement (0.86). Yet, its temporal fidelity was poor (DTW: 0.57). The most biomedically coherent instance remained Run s1r0, which minimized diagnosis trajectory error (DTW: 0.25) and effectively balanced multivariate structure.

This dissociation demonstrates that maximizing standard statistical metrics does not guarantee, and may even compete with, the preservation of the complex biomedical patterns across time.

### 3.5 Algorithmic Bias

**FIGURE 1:**
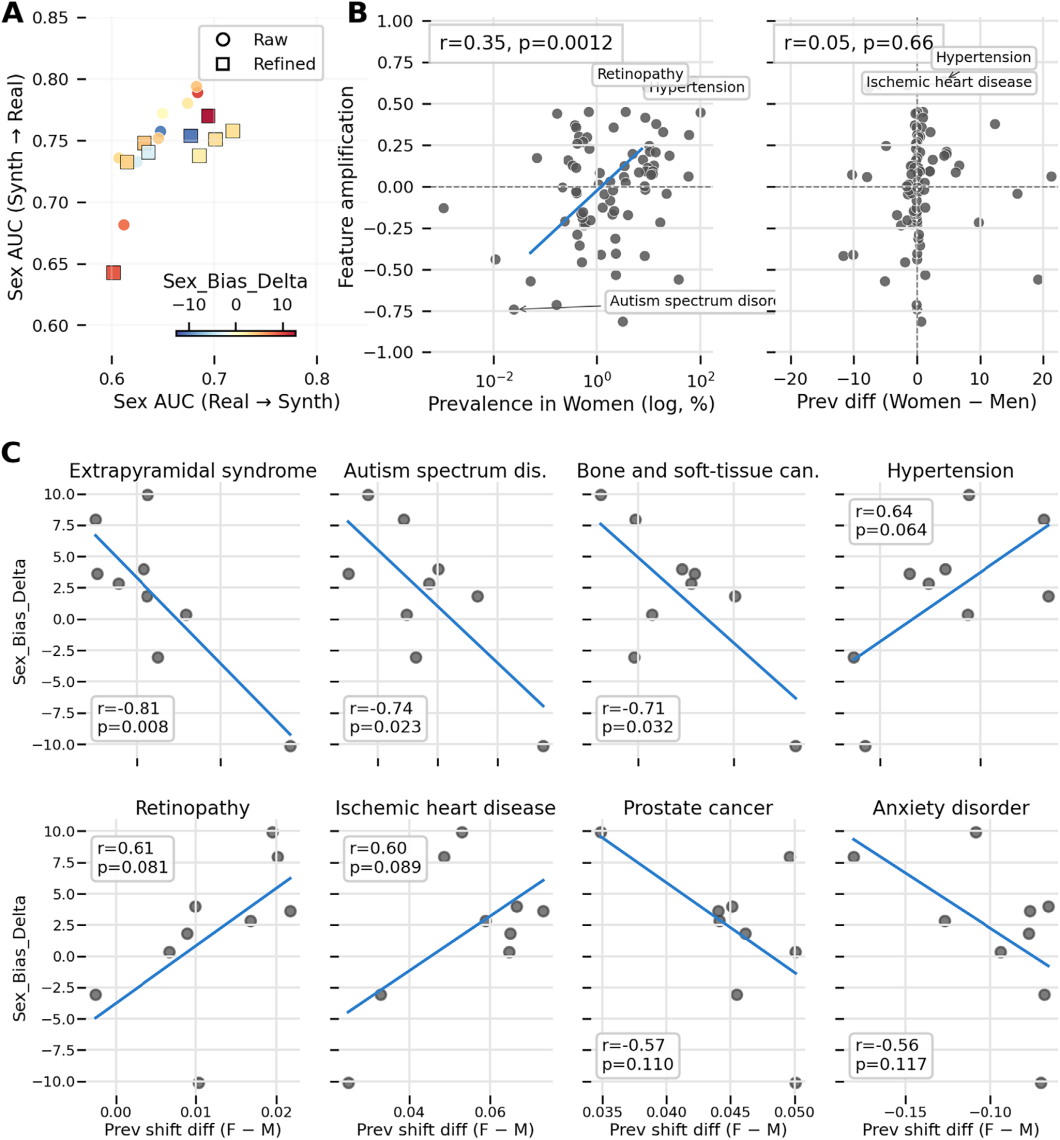
Algorithmic Bias. (A) Sex Signal Portability. Comparison of sex predictability (AUROC) for *Real to Synthetic* and *Synthetic to Real* transfers. Points above the diagonal demonstrate that synthetic data amplifies sex‐discriminative signals, the color shows the prevalence shift toward women (except for two runs). (B) Determinants of Amplification. (Left) Feature amplification correlates positively with disease prevalence in women (r ≈ 0.3), indicating common diseases track with demographic skew. (Right) No correlation exists with the baseline sex prevalence difference (r ≈ 0.05), showing bias is prevalence‐driven rather than sex‐driven. (C) Top Bias Drivers. Differential prevalence shifts reveal a divergence: common metabolic conditions positively align with cohort bias, whereas rare pathologies exhibit inverted shifts, reflecting partial mode collapse for sparse data.

To examine the origin of the observed variability in the synthetic data generation process, we evaluated sex predictability, feature amplification, and disease‐specific prevalence shifts. As shown in Figure 1A, the generative runs display a consistent over‐representation of women in the synthetic cohorts relative to the real data. The shift size (percentage‐point difference in female prevalence) varies across replicas but remains a recurrent property of the generator. Moreover, we observed that models trained on synthetic data and tested on real data consistently yielded higher sex‐prediction accuracy (AUROC ≈ 0.65–0.80) compared to models trained on real data and tested on synthetic data (AUROC ≈ 0.60–0.72), suggesting that the generative process may strengthen sex‐discriminative features.

To explore a possible mechanism, we analyzed feature amplification in relation to disease characteristics (Figure 1B). We observed (Figure 1B‐left) a moderate positive correlation (r = 0.35, p = 0.0012) between feature amplification and the logarithmic prevalence of each feature (disease), while no significant correlation (r = 0.05, p = 0.66) was found between feature amplification and the baseline prevalence difference between sexes (Figure 1B‐right). This suggests a differential simplification by sex tendency, reflecting that bias amplification is linked more to the overall frequency of a diagnosis rather than its biological association with a specific sex.

Finally, we observed (Figure 1C) a distinct model behavior based on the nature of the features while analyzing the diseases showing stronger amplification signals. On the one hand, metabolic and high‐prevalent conditions like Hypertension (r = 0.64) and Retinopathy (r = 0.61) acted as positive drivers, following the overall prevalence of the cohort. On the other hand, rare conditions like Extrapyramidal syndrome (r = –0.81) and Autism spectrum disorder (r = –0.74) acted as negative drivers. This confirms a partial model collapse, while the model successfully resolves the demographics for common comorbidities (the dominant modes), it frequently decouples rare disease trajectories, which leads to inverted sex‐prevalence shifts.

Furthermore, Table S16 summarizes the effect sizes between real and synthetic training set replicates for both comorbidity prevalence and age at diagnosis. Overall, most features exhibit small to negligible effect sizes; however, a subset shows moderate differences, with effect sizes approaching 0.2. These features predominantly include either relatively high‐prevalence diagnoses with mild sex imbalance, such as osteoporosis or asthma, or low‐prevalence conditions, such as hepatic steatosis or urinary lithiasis. In contrast, strongly sex‐specific diagnoses, including prostate and cervical cancer, remain comparatively stable between real and synthetic data. This pattern further supports the interpretation that bias amplification in the generative process is primarily driven by feature frequency rather than by intrinsic biological associations with sex. Furthermore, amplification of prevalence bias is mirrored by a corresponding amplification in age‐at‐diagnosis bias, as the relative ordering of effect sizes for age at diagnosis closely follows that observed for disease prevalence. Finally, our end‐point shows the largest effect size between real and synthetic datasets. This likely reflects the truncation of patient trajectories at this stage, amplifying any bias introduced during the disease trajectory generation process. All the univariate comparisons (means and proportions) between Synthetic and Real sets can be found in Supporting Information tableone.xlsx.

From now on, we will focus on analyzing the results for the refined SSMR synthetic cohorts, as they are used for testing the end‐point prediction models. To provide a visual representation of their fidelity, we illustrate the univariate distributions for both a highly prevalent disease (hypertension) and a low‐prevalence one (Kaposi's sarcoma).

**FIGURE 2:**
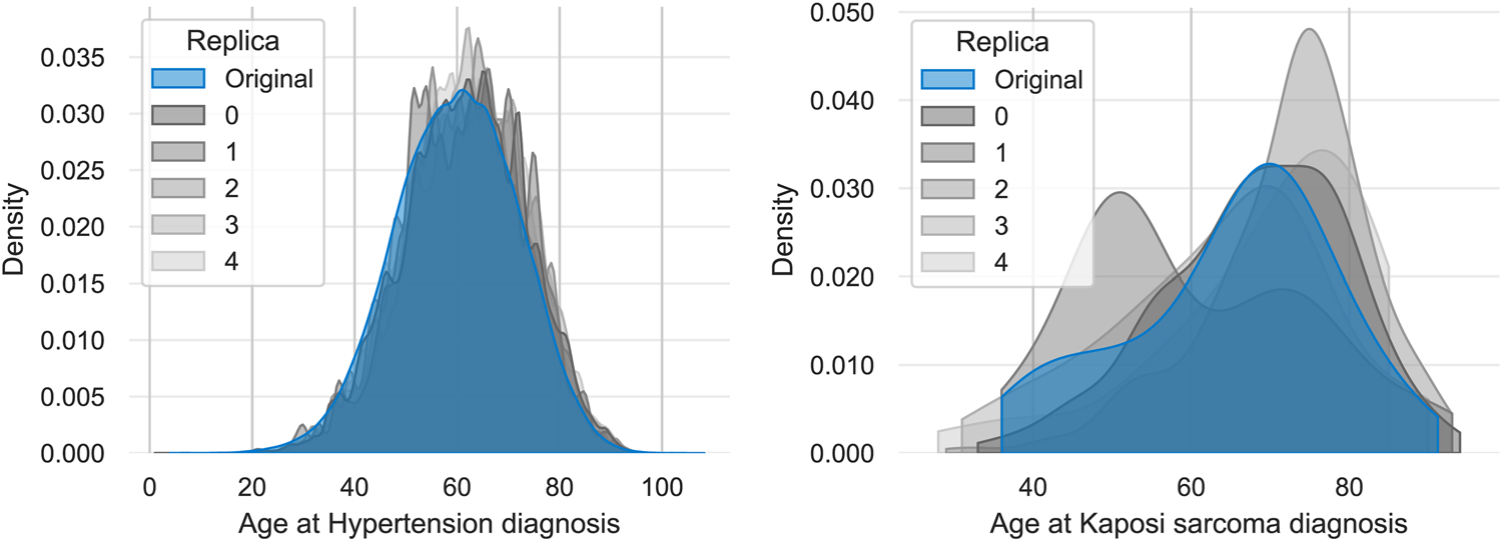
Comparison of Age at Diagnosis Distributions (SSMR‐Refined). The density plots compare the distribution of age at diagnosis for a high‐prevalence comorbidity (Hypertension, left) and a low‐prevalence one (Kaposi's sarcoma, right) between the original data (blue) and three synthetic replicas (grey shades).

**FIGURE 3:**
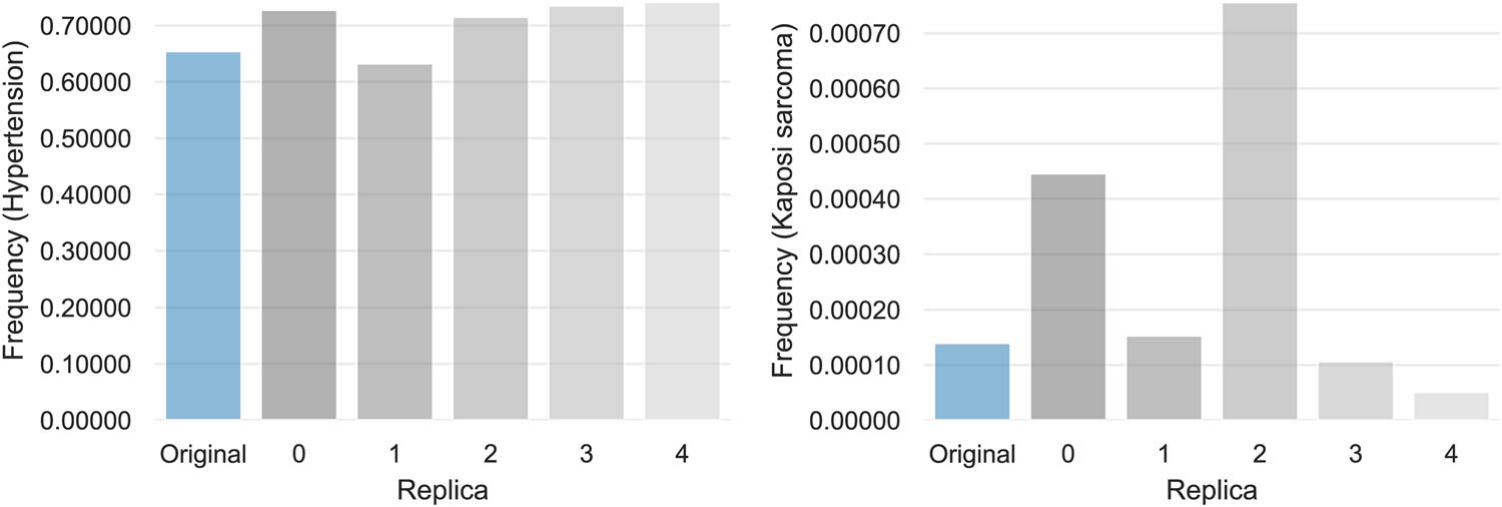
Comparison of Categorical Disease Frequencies (SSMR‐Refined). The bar charts compare the frequency of a high‐prevalence comorbidity (Hypertension, left) and a low‐prevalence one (Kaposi's sarcoma, right). The frequency in the original dataset (blue) is shown alongside the frequencies from three synthetic replicas (grey shades).

Figure 2 shows a comparison of the age at diagnosis distributions between the real and synthetic cohorts, while Figure 3 compares the categorical distributions of the presence and absence for each disease.

These plots visually confirm the high fidelity captured by the quantitative metrics, and the underlying problems when dealing with low‐prevalence diseases. A more detailed study was conducted to identify model's fidelity (relative errors) in low‐prevalence comorbidities (See Figure S6). In this sense, it is observed that relative error substantially increases for diagnoses prevalence below 0.05. In fact, excluding diagnoses below this threshold in the SSMR‐Refined cohorts clearly enhanced the correlation of prevalence in the synthetic datasets from 0.989 to 0.994 while the root mean square relative error (RMSE) was reduced from 0.653 to 0.461. Alternatively, a less restrictive prevalence threshold of 0.013 may be considered to avoid excluding an excessive number of comorbidities. Likewise trends were observed for the MSSR‐Refined cohorts (Figure S17), where the same prevalence thresholds (0.05 and 0.013) were identified.

### 3.6 Sex‐Based Differences in Diagnosis Rates Across the Lifespan

**FIGURE 4:**
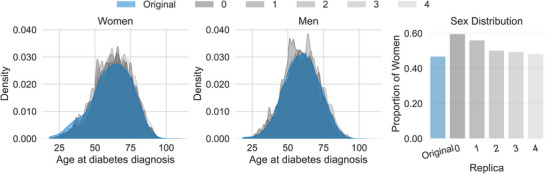
Comparison of Age at Diabetes Diagnosis and Sex Distribution (SSMR‐Refined). The density plots compare the age at diagnosis for women (left) and men (right) between the original data (blue) and five synthetic replicas (grey shades). The bar chart (right) shows the proportion of women in the original cohort vs. the five replicas, demonstrating the model's ability to capture demographic distributions, although women are generally overrepresented.

The model's ability to capture key demographic distributions was further assessed by stratifying by sex. As shown in Figure 4, the age at diabetes diagnosis distributions for both women (Figure 4 ‐ right) and men (Figure 4 ‐ center) are well‐replicated, with the synthetic replicas closely matching the shape and range of the original data. However, the overall sex distribution shows a slight bias (Figure 4 ‐ right), with women being generally overrepresented in the synthetic replicas compared to the original cohort.

To move beyond purely statistical metrics, we performed a domain‐specific validation by examining whether the synthetic data could replicate known, complex biomedical patterns. We analyzed the difference in the rate of comorbidity diagnoses between women and men across their lifespan, a trajectory with established clinical patterns [45, 46, 47, 48], especially in cardiovascular and metabolic‐driven diseases.

**FIGURE 5:**
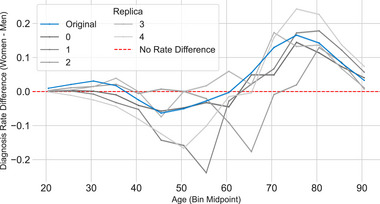
Sex‐Based Differences in the Rate of Diagnosis Across the Lifespan (SSMR‐Refined). The plot displays the absolute difference (and 95% CI), in the per‐person diagnosis rate (Women–Men) across 5‐year age intervals. The trend for the original dataset is shown in blue, with simulated data replicas in grey. The dashed red line at y = 0 indicates no difference between the sexes. The results highlight that the diagnosis rate is higher among women in early adulthood and later life, while men exhibit a higher rate during middle age. The synthetic replicas follow the pattern, specially #0. Other replicas show a similar but delayed pattern.

As shown in Figure 5, the original data (blue line) reveals a distinct pattern: diagnosis rates are slightly higher among women in early adulthood, become higher in men during middle age (approx. 45–65), and then shift back to being significantly higher in women in later life. When evaluating the synthetic replicas, we observed considerable variability in their ability to reproduce this trajectory. Some replicas, particularly replica #0, closely followed the real‐world pattern. Others, however, showed divergent or delayed patterns (e.g., replica #3) or exhibited erratic, clinically implausible fluctuations (e.g., replica #4). This demonstrates that while some replicas are biomedically sound in this regard, others, despite having high statistical fidelity scores, fail this crucial domain‐specific test. A similar pattern of inconsistency was observed when analyzing the raw synthetic replicas in both SSMR and MSSR analysis (Figures S5 and S12, respectively) and the refined cohort of the MSSR analysis (Figure S16).

The synthetic replicas showed considerable variability in their ability to reproduce this trajectory. To quantify this, we calculated the Dynamic Time Warping (DTW) distance between the original trajectory and each replica. For the refined cohort (Table 3), the DTW distances ranged from a low of 0.25 (replica 0) to a high of 0.64 (replica 4), confirming that some replicas captured the biomedical pattern with much higher fidelity than others (a pattern also present in the raw cohort analysis, see Table S2). To ensure this variability was not an artifact of the initial data split, we conducted a sensitivity analysis using multiple train‐test splits (see Tables S8 and S9 for the raw and synthetic cohorts DTW distances of the MSSR analysis). The DTW distances between different real‐data splits were very low (mean ≈ 0.01), indicating the pattern is stable in the real data. In contrast, the distances between each real split and its paired synthetic replica were an order of magnitude higher (mean ≈ 0.44), confirming that the observed variability in biomedical plausibility is a characteristic of the generative model itself.

**TABLE 3:** DTW Distances for Sex‐Based Diagnosis Rate Trajectories (SSMR‐Refined). The table lists the calculated Dynamic Time Warping (DTW) distance between the diagnosis rate difference trajectory of the original data and each of the five synthetic replicas for the refined cohort. A lower distance indicates a higher similarity to the original biomedical pattern.

Type	Replica	DTW Distance
Orig‐Rep	4	0.6413677268
Orig‐Rep	3	0.2958196152
Orig‐Rep	2	0.3706055580
Orig‐Rep	1	0.6279947833
Orig‐Rep	0	0.2507168231

These observed inconsistencies in biomedical plausibility, which are not captured by the aggregate fidelity scores in Table 1, highlight the critical need for domain‐specific evaluation frameworks.

### 3.7 Train‐Synthetic, Test‐Real performance

We evaluated the practical utility of synthetic cohorts through a Train‐Synthetic, Test‐Real (TSTR) strategy. This involved training models on synthetic data to predict chronic kidney disease (CKD) onset and comparing their predictive accuracy and stability against models trained on real data. This quantifies the usefulness of generated data for downstream machine learning.

#### 3.7.1 Static Predictive Performance

**FIGURE 6:**
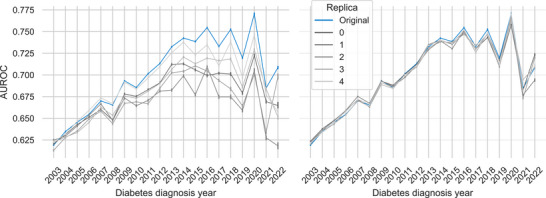
Predictive Performance (AUROC) by Year of Diabetes Diagnosis (SSMR‐Refined). **(Left Panel)** Compares the performance of the model trained on the original data (blue) against models trained on five independent synthetic replicas (grey shades). All models show improving performance over time, with the synthetic replicas consistently tracking the original model's performance while maintaining a slight performance gap. **(Right Panel)** Shows the performance of five different models, each trained on an augmented dataset combining the original data with one of the synthetic replicas. The performance of these hybrid models practically overlaps with the performance of the model trained on the original data alone, indicating that data augmentation did not provide additional predictive value in this scenario.

The predictive performance of the models, when disaggregated by the year of diabetes diagnosis, reveals a consistent trend of improving AUROC scores over time (Figure 6). This trend is observed for models trained on both real and synthetic data, likely reflecting the increasing quality and completeness of the underlying real‐world data in more recent years. As shown in the figure, the models trained on synthetic replicas consistently track the performance of the model trained on original data, albeit with a slight but persistent performance gap. Furthermore, the performance of the hybrid model, trained on an augmented dataset of both real and synthetic data, practically overlapped with the model trained on real data alone, indicating that data augmentation did not provide additional predictive value in this scenario. While there is some variability between the individual synthetic replicas, they generally cluster together below the performance of the original data model. The small error bars for all models indicate high stability across the cross‐validation splits, confirming that the observed performance is a reliable measure for each respective training set. Similar trends are observed when AUROC scores are analyzed separately by sex (Figure S18). Model performance is somewhat more stable in men than in women for models trained on the original data, a pattern that is also replicated in models trained on synthetic data. Consistent with the overall AUROC results, augmenting the data with synthetic samples does not yield performance improvements when the sexes are analyzed independently. In Tables S3–S7, we report the overall number of patients evaluated each year, as well as according to sex, along with the mean and standard deviation of the AUROC scores.

The MSSR analysis shows similar trends, suggesting that the predictive performance for CKD onset is not due to the initial patient split. Refer to Figure S19 for the AUROC trend by year of diabetes diagnosis according to sex, and Tables S10–S14 for the overall and sex independent statistical summary.

##### 3.7.1.1 Representation Stability

**FIGURE 7:**
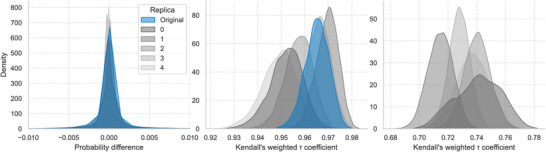
Representation stability (SSMR‐Refined). (Left panel) The distribution of prediction probability differences between models trained on cross‐validation splits and the full dataset is tightly centered on zero, indicating high consistency. **(Center panel)** The internal stability of feature importance rankings within both real and synthetic data models is high, with median hyperbolic weighted Kendall's tau values greater than 0.9. **(Right panel)** The distribution of the Kendall's tau coefficient comparing feature rankings between real and synthetic models shows strong agreement, with a median value between 0.71 and 0.76 depending on the replica, confirming that the synthetic data preserves the clinical interpretability of the model.

The stability of the models was further assessed by comparing their internal behavior. The consistency of prediction probabilities was evaluated by comparing the outputs from models trained on cross‐validation splits against the model trained on the full dataset. The resulting distributions of probability differences were tightly centered around zero for models trained on both real and synthetic data (Figure 7, left panel), indicating a high degree of consistency and minimal variation due to data sampling during training.

Furthermore, to evaluate if the synthetic data preserved the clinical relationships necessary for prediction, we analyzed the stability of feature importance rankings. First, we assessed the internal consistency of the models by comparing feature rankings across the cross‐validation splits of the same dataset (Figure 7, center panel). Both the real and synthetic models produced highly stable rankings, with median hyperbolic weighted Kendall's tau values greater than 0.95. Second, we compared the feature rankings between the real and synthetic models (Figure 7, right panel). The distribution of the Kendall's tau coefficient showed a strong agreement across all the replicas, with a median value ranging from approximately 0.71 to 0.75. This indicates that the models trained on synthetic data prioritize the same clinical predictors as the model trained on real data, suggesting that the synthetic data generation process preserves the interpretability and clinical coherence of the resulting models.

Similar trends were observed in the multiple split, single replica (MSSR) analysis (Figure S21), confirming that these stability findings are robust and independent of the initial train‐test data split.

#### 3.7.2 Trajectory‐Based Predictive Performance

**FIGURE 8:**
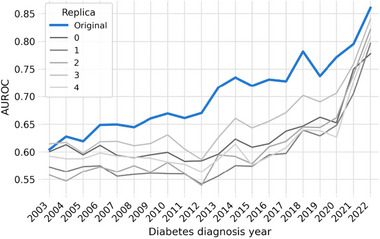
Predictive Performance (AUROC) for trajectory‐based LSTM models by Year of Diabetes Diagnosis (SSMR‐Refined). Both models trained on synthetic replicas (gray shades) and original data (blue) exhibit similar temporal improvement in performance, with slightly lower performance in synthetic. A reduced performance gap is observed for more recent diagnosis years, suggesting closer alignment between synthetic and real data trajectories in periods with improved data completeness.

Consistent with the previous section, the trajectory‐based models also exhibit an acceptable progressive improvement in AUROC when stratified by year of diabetes diagnosis (Figure 8). Although performance remains slightly lower, models trained on synthetic replicas closely follow the same temporal trend as those trained on the original data, with a reduced performance gap in more recent diagnosis years. Both the consistent performance improvement and the convergence between synthetic and real models suggest synthetic data is able to consistently capture relevant characteristics from longitudinal trajectories, particularly for recent years where trajectories exhibited more complete and high‐quality records. Similar performance progression is also observed for original data and synthetic splits in the MSSR analysis, supporting the consistency in performance for CKD independently of the selected split (Figure S20).

## 4 Discussion

In this work, we have demonstrated that high‐fidelity synthetic data, generated from a large, real‐world cohort of over a million diabetic patients, can successfully replicate the predictive performance of real data in a clinically relevant task. Our primary finding, that a model trained exclusively on synthetic data to predict the onset of Chronic Kidney Disease (CKD) can closely approach the performance of a model trained on original data, is strongly supported by the literature. The Train‐Synthetic, Test‐Real (TSTR) methodology is easily interpreted and provides clear utility information; for instance, it has been suggested that synthetic data utility is sufficient if the predictive performance does not decrease by more than 5% [49]. Our results, with a performance decrease of approximately 4.1% (AUROC of 0.70 vs. 0.73), meet this criterion, validating the use of our synthetic data as a reliable proxy for sensitive clinical information in predictive modeling, in league with modern large language model‐driven generative processes [50].

A key strength of our study is the demonstration of both the utility and the robustness of the generated data. The utility is clearly shown by the minimal performance gap in the TSTR evaluation. More than just achieving a high AUROC, the models trained on synthetic data also proved to be interpretable and clinically coherent. This aligns with early work that demonstrated the importance of preserving variable associations in synthetic EHRs [14]. The strong agreement in feature importance rankings, evidenced by a high weighted Kendall's tau, indicates that the synthetic data preserved the complex relationships between comorbidities that are critical for clinical risk stratification. This ability to replicate not just predictions but also the underlying evidence is essential in high‐stakes scenarios such as healthcare [49, 50]. Furthermore, the consistency across the five independent generation runs speaks to the robustness of our BPS‐driven DAAE generative process, suggesting that the model reliably captures the dominant patterns within the training data.

However, a central theme emerging from our results is that a reliance on problem‐agnostic statistical metrics alone is insufficient for fully validating synthetic data in complex biomedical applications. This aligns with a growing consensus advocating for task‐based evaluation over simple statistical fidelity [51]. As can be inferred from the excellent median performance reported in Table 1, our synthetic cohorts appear statistically robust. Yet, a more biomedically‐oriented approach reveals underlying inconsistencies. For example, when comparing the diagnostic rate trajectories for key comorbidities over time, we observe that not all replicas behave consistently from a clinical standpoint, with some showing implausible temporal patterns despite their high aggregate fidelity scores. This highlights the critical need to complement standard quality assessments with domain‐specific, problem‐oriented evaluations to ensure the generated data is not only statistically similar but also biomedically sound and reliable for downstream research.

Notably, our fidelity‐bias analysis suggests that a shared structural tendency drives the inconsistencies of the generative modeling, especially the dichotomies between the privacy and the sex bias: the model's preference for capturing regular, high‐frequency patterns (high‐signal, low‐entropy modes). Also, the increasing errors observed for low‐prevalence comorbidities suggest that additional strategies may be required to better control synthetic data fidelity in this range, particularly below the proposed prevalence thresholds.

On the one hand, while patient matches between the synthetic and training data ranged from 17.9% to 22% across validation runs, according to the new row synthesis scores, most matches occurred almost exclusively in structurally simple profiles (average diagnose count ranging from 0.72 to 0.86). This indicates that the matches primarily consisted of patients with minimal comorbidities (typically limited to inclusion criteria), mirroring the intrinsic redundancy of the real training data where approximately 7.9% of rows were already duplicates.

On the other hand, the Membership Inference Attack AUROC were near random on raw cohorts (AUROC ≈ 0.50). The refined cohorts showed AUROC < 0.5 due to an inverted attacker score; after orientation correction the effective performance was AUROC ≈ 0.60–0.61 (Δ ≈ 0.10–0.11), indicating weak, non‐actionable membership signal and no evidence of memorization. Instead, they correspond to the correct density estimation of broad, high‐anonymity population patterns rather than uniquely identifiable patient trajectories.

Regarding predictive utility, one of the anticipated benefits of synthetic data is its potential for data augmentation. However, in our study, the hybrid model trained on a combination of real and synthetic data offered no performance benefit over the model trained on real data alone. This result, while seemingly counterintuitive, places our work within a broader, ongoing debate in the literature, where the application of data augmentation to tabular clinical data has yielded a wide spectrum of results. Some studies report significant benefits; for instance, using diffusion models for lab values [52] or LLMs for patient‐trial matching [53]. Others find more marginal or context‐dependent gains, noting critical trade‐offs between metrics like sensitivity and overall accuracy [54, 55], or for example in [50] the authors propose a hierarchical autoregressive language model and report marginally better results for data‐augmented chronic classification models. Conversely, and in line with our findings, a notable portion of the literature reports no benefit. A key study on clinical generative modeling [56] found no statistically significant evidence that complex generative models (GANs, VAEs) outperformed simpler baselines on realistic clinical datasets. The contradictions in these findings stem from the specific augmentation methods used, the evaluation metrics chosen, and the inherent characteristics of the data itself, particularly its dimensionality [57]. The fact that our BPS‐driven DAAE model did not yield an augmentation benefit aligns with findings where even sophisticated models do not guarantee meaningful improvement [17, 56].

However, this *data‐saturation* finding does not diminish the value of synthetic augmentation in limited data scenarios, such as rare diseases, underrepresented groups or small single‐center cohorts. In these scenarios characterized by data scarcity, synthetic data can serve as a regularization tool, mitigating class imbalance and bridging the gaps in the datasets [56].

Ultimately, the finding that data augmentation yielded no performance benefit clarifies that in data‐rich environments (on the limit of *data saturation*), the primary value of synthetic data is secure accessibility rather than enhancement (*data democratization*). This insight supports the multi‐faceted evaluation strategy employed in this study (combining predictive utility, feature rank importance distribution, bias forensics, and generative robustness) aligns with the comprehensive frameworks proposed for building trust in synthetic clinical data [51, 58]. However, establishing this trust requires acknowledging that universal thresholds for “acceptable” privacy do not exist. The threshold for safely releasing synthetic data ultimately depends on the assumed adversary model, the availability of auxiliary data, the inherent sensitivity of the original dataset, and the specific institutional governance context. Therefore, multiple complementary metrics must be considered jointly, moving beyond standard machine learning and statistical fidelity to explicitly validate known biomedical‐driven facts. By demonstrating stability across independent runs, a key practice emphasized by [59], and validating both the statistical and clinical coherence of the data, our work provides a robust blueprint for the validation of large‐scale synthetic RWD. To operationalize this blueprint and contextualize the specific boundaries of our privacy audits, we outline the limitations of our approach and provide practical recommendations for deployment in the following section.

## 5 Limitations and Practical Recommendations

### 5.1 Limitations

This study, while comprehensive, has several limitations that should be considered when interpreting the results.

First, the significant computational cost of the generative procedure restricted our analysis to five independent replicas. While the results across these runs were largely consistent, providing evidence for the model's robustness, a larger number of replicas would be necessary to fully characterize the variability of the generative process and the entire distribution of possible synthetic datasets.

Second, our analysis revealed a persistent algorithmic bias in the sex distribution of the synthetic data. Beyond women being generally overrepresented, our analysis identified domain‐specific fidelity limits driven by the model's focus on highly frequent patterns. These issues also affected temporal trends: across some independent runs, the model did not reliably reproduce biologically plausible diagnosis trajectories when stratified by sex. While the model robustly captures common metabolic conditions across runs, it displays distinct error patterns for more complex phenotypes. Specifically, it struggles to accurately reproduce the prevalence and distribution of neuropsychiatric conditions in male cohorts and rare somatic malignancies in female cohorts. This suggests that the model's mode‐seeking behavior favors stable, high‐signal patterns at the expense of less frequent but clinically important subpopulations.

Third, while the model performed well on aggregate, it struggled to accurately replicate trends for very rare events. This was most evident in the correlation similarity metric, where the model failed to capture the correct relationship for comorbidities with very low prevalence. This limitation suggests that while the current synthetic cohort is suitable for studying common disease trajectories, its utility for research focused on rare diseases may be limited. Although a fidelity threshold has been proposed as a boundary for this limitation, additional future strategies are proposed here to address it in a more efficient manner. Specifically, a prevalence‐aware oversampling could increase representation of rare diagnoses during training without distorting overall distribution. Alternatively, providing specialized loss functions that penalize large deviations for low‐prevalence conditions could improve sensitivity to rare events.

Therefore, although these problems did not appear to significantly impact the primary prediction task, it highlights the ongoing challenge of ensuring fairness and mitigating bias in generative models. Other, more subtle biases may exist that were not captured by our evaluation framework.

Fourth, our audits focus on zero‐knowledge MIA and simulated disclosure risk, but do not cover record‐linkage with rich auxiliary data [60], attribute‐inference/model‐inversion under strong priors [61], or targeted attacks on small subpopulations [62]; these require separate, context‐specific safeguards.

Finally, our study utilized a single generative architecture, the DAAE. While effective, it is one of many possible approaches. Other advanced architectures, such as transformer‐based models or diffusion models, might capture different aspects of the complex temporal data more effectively and could be explored in future work.

### 5.2 Practical Recommendations

Our work demonstrates that high fidelity and structural scores alone cannot guarantee that specific instances preserve complex, non‐linear clinical trajectories. These findings are more evident in the contrast between high‐signal well reproduced metabolic conditions, and rare phenotypes, which featured mode collapse, feature erosion and inverted sex‐prevalence shifts: limitations that become clearer given the practical implausibility of testing or constraining the model for every biomedical property individually.

The variability we observed when reproducing complex biomedical patterns, despite high statistical fidelity, leads us to propose a series of recommendations for shifting how synthetic data is validated in clinical scenarios:

#### 5.2.1 Replication

Characterize the stochasticity of the model by generating multiple independent replicas to identify stable signal patterns.

#### 5.2.2 Clinical Unit Testing

Define domain‐specific quantitative checks that replicate biological ground truths in addition to the standard distributional, geometric and privacy metrics.

#### 5.2.3 Bias Auditing

Acknowledge that utility is not uniform across the synthetic population. Audit the synthetic data for the prevalence tier of the target variables. Synthetic cohorts may fail to capture the complex trajectories of low‐prevalence diseases or underrepresented groups, while reliably capturing common comorbidities groups present in the real dataset.

#### 5.2.4 Selection

The combination of multiple replicas, bias auditing, clinical unit testing must serve as a gating criterion. Discard replicas that display implausible biomedical behavior and select only those that pass domain‐specific checks, in addition to having high scores in the common metrics.

## 6 Conclusions

In this study, we successfully generated high‐fidelity synthetic patient trajectories from a large‐scale, real‐world diabetes cohort. Our results demonstrate that this synthetic data can effectively replicate the performance of real data in a clinical prediction task, preserving both predictive accuracy and the interpretability of the resulting models. However, our work also highlights that problem‐agnostic statistical metrics are insufficient for a complete validation. Bias and domain‐specific evaluations, such as the analysis of clinical trajectories, revealed inconsistencies not captured by standard fidelity scores, underscoring the need for a multi‐faceted validation approach.

Ultimately, in a data‐rich setting like ours, the primary value of synthetic data lies not in performance enhancement through augmentation, but in its capacity to broaden research opportunities. By providing a robust, privacy‐preserving proxy, this work paves the way for a wider community of researchers to leverage valuable clinical resources, fostering innovation and collaboration in biomedical research.

## Conflicts of Interest

The authors declare no conflicts of interest.

## Supporting information


**Supporting File**: advs74764‐sup‐0001‐SuppMat.zip.

## Data Availability

The Synthetic Clinical Health Records Challenge, presented in 2023 for the first time in the CAMDA conference, makes available to the interested delegates 1 million EHRs to develop endpoint predictors that can be further validated with the real patient dataset. Data available at: https://bipress.boku.ac.at/camda2025/the‐camda‐contest‐challenges.
