# Supplementary material for: High‐Fidelity Synthetic Data Replicates Clinical Prediction Performance in a Million‐Patient Diabetes Cohort
Source: Adv Sci (Weinh). 2026 Mar 16;13(29):e16196. doi: 10.1002/advs.202516196 (PMC13205598; doi:10.1002/advs.202516196)
Supplement: Supplementary file 1 — Supporting File: advs74764‐sup‐0001‐SuppMat.zip. [file ADVS-13-e16196-s001.zip › advs74764-sup-0001-SuppMat.pdf]

Supplementary Material: High-Fidelity Synthetic Data Replicates Clinical Prediction Performance in a Million-Patient Diabetes Cohort

Francisco Ortuño[1,+,*], Victor de la Oliva Roque[2,3,+], Javier-Ignacio Ramirez-Lopez[1,], David P. Kreil[4], Joaquín Dopazo[2,3,*], Carlos Loucera[2,3,5,*]

1. Department of Computer Engineering, Automation and Robotics, University of Granada, Granada, Spain.

2. Platform for Computational Medicine, Andalusian Public Foundation Progress and Health-FPS, Seville, Spain

3. Institute of Biomedicine of Seville, IBiS, University Hospital Virgen del Rocío/CSIC/University of Sevilla, 41013 Sevilla, Spain

4. Institute of Molecular Biotechnology, Department of Biotechnology and Food Science, BOKU University, Vienna, Austria

5. Department of Computer Science and Artificial Intelligence, Universidad de Sevilla, Avda. Reina Mercedes s/n, 41012, Sevilla, Spain

+: equal contribution

*: corresponding author

- fortuno@ugr.es
- joaquin.dopazo@juntadeandalucia.es
- clou@us.es

Disease Codes

Supplementary Table 1

A. Comorbidities

Internal Code	Grouped comorbidity	ICD-10
101	HIV	B20, B97.35, Z21, V08
201	Head and neck cancer	C00 ,C01, C02, C03, C04, C05, C06, C07, C08, C09, C10, C11, C12, C13, C14, C30 ,C31, C32, C76.0

202	Stomach cancer	C16, C49.A2, C7A.092, D00.2
203	Colorectal cancer	C18, C19, C20, C21, C49.A4, C49.A5, C7A.0
204	Liver and biliary tract cancer	C22, C23, C24, D01.5
205	Pancreatic cancer	C25
206	Bronchial and lung cancer	C34, C7A.090, D02
207	Thyroid cancer	C73, D09.3
208	Bone and soft tissue cancer	C40, C41, C47, C49
209	Skin melanoma	C43, D03
210	Breast cancer	C50, D05
211	Uterine cancer	C54, C55, D07.0
212	Cervical cancer	C53
213	Ovarian cancer	C56
214	Prostate cancer	C61, D07.5
215	Testicular cancer	C62
216	Kidney and renal pelvis cancer	C64, C65, C7A.093
217	Bladder cancer	C67.0, C67.1, C67.2, C67.3, C67.4, C67.5, C67.6, C67.7, C67.8, C67.9, D09.0
218	Hodgkin's disease	C81
219	Non-Hodgkin’s lymphoma	C82, C83, C84, C85, C86, C88.4, C96.4, C96.9, C96.Z
220	Leukemia	C90, C91, C92, C93, C94, C95
221	Immunoproliferative cancer	C88, C90
222	Kaposi's sarcoma	C46
401	Diabetes	E10, E11
402	Dyslipidemia	E78
403	Hypothyroidism	E00, E01.8, E02, E03, E07.1, E89.0
404	Obesity	E66, Z68, V85
500	Dementia	F01.5, F02.8, F03.9, F10.27, F10.97, F13.27, F13.97, F18.97, F19.17, F19.27, F19.97, G30, G31.0 G31.1, G31.83
501	Other organic mental disorder	F04, F05, F06 F07, F09, F10, F11, F12, F13, F14, F15, F16, F18, F19

502	Alcohol dependence	F10.10, F10.11, F10.120, F10.121, F10.129, F10.130, F10.131, F10.132, F10.139, F10.14, F10.150, F10.151, F10.159, F10.180, F10.181, F10.182, F10.188, F10.19, F10.20, F10.21, F10.220, F10.221, F10.229, F10.230, F10.231, F10.232, F10.239, F10.24, F10.250, F10.251, F10.259, F10.26, F10.27, F10.280, F10.281, F10.282, F10.288, F10.29, F10.920, F10.921, F10.929, F10.930, F10.931, F10.932, F10.939, F10.94, F10.950, F10.951, F10.959, F10.96, F10.97, F10.980, F10.981, F10.982, F10.988, F10.99, G31.2, G62.1, G72.1, K70
503	Tobacco dependence	F17
504	Dependence on other substances	F11, F12, F13, F14, F15, F16, F18, F19
505	Schizophrenic disorder	F20, F21, F22, F23, F24, F25, F28, F29
506	Mood disorder	F10, F11, F14, F15, F16, F18, F19, F30, F21, F32, F33, F34, F39
507	Anxiety disorder	F10.180, F10.280, F10.980, F12.180, F12.280, F12.980, F13.180, F13.280, F13.980, F14.180, F14.280, F14.980, F15.180, F15.280, F15.980, F16.180, F16.280, F16.980, F18.180, F18.280, F18.980, F19.180, F19.280, F19.980, F40, F41, F42, F43, F44, F45, F48
508	Eating disorder	F50
509	Other functional disorder	F51, F52, F53, F54, F55, F59, F98.5
510	Personality and adult behavior disorder	F60, F63, F65, F66, F68, F69
511	Intellectual disability	F70, F71, F72, F73, F78, F79
512	Autism spectrum disorder	F84.0, F84.2, F84.3, F84.5, F84.8, F84.9
513	Other developmental disorder	F80, F81, F82, F88, F89
514	Early childhood adolescence disorder	F90, F93, F94, F95, F98
601	Epilepsy	G40
602	Parkinson's disease	G20
603	Extrapyramidal syndrome	G10, G21, G23, G24, G25, G26, G71.1
604	Neurological disease with motor deficit not stroke	E08.4, E75, G04.1, G11, G12, G13, G31.8, G31.9, G32, G35, G36, G37, G60, G61, G62, G63, G65, G70, G71, G72, G73, G80, G81, G82, G83, G90, G91, G93.7, G93.89, G94, G95, G99, Q07.8, Q07.9
701	Age-related macular degeneration	H35.3
702	Glaucoma	H21.82, H40, H42, Q15.0

703	Retinopathy	E08, E09, E10, E11, E13, H30 H31.0, H33, H34, H35.0, H35.1, H35.2, H35.4, H35.5, H36.6, H35.7, H35.8, H35.9, H36
902	Ill-defined and other cerebrovascular disease	G46.7, G46.8, I67.1, I67.2, I67.5, I67, I68
904	Occlusion or stenosis of precerebral arteries	I65
905	Sequelae of cerebrovascular disease	I69
906	Limb arteriopathy	E08.5, E09.5, E10.5, E11.5, E13.5, I70, I73
907	Intraabdominal arteriopathy	I70.1, K55
908	Peripheral and visceral artery aneurysms	I67.0, I71, I72, I77.7, I77.8, I79
909	Atrial fibrillation	I48.0, I48.11, I48.19, I48.20, I48.21, I48.91
910	Ischemic heart disease	I23, I25, T82.2
911	Acquired valvular disease	A52.03, I05, I06, I07, I08, I09, I34, I35, I36, I37, I38, I39, I42.4, T82.01, T82.0, T82.6, Z95.2, Z95.3, Z95.4, V42.2, V43.3
912	Congenital heart and circulatory anomaly	P29.3, Q20, Q21, Q22, Q23, Q24, Q25, Q26, Q27, Q28
913	Hypertension	I10, I11, I12, I13, I15, I67.4,
914	Heart failure	I09.81, I11.0, I13.0, I13.2, I27.0, I27.2, I27.8, I27.9, I50, I97.0, I97.1
1001	Chronic Obstructive Pulmonary Disease (COPD)	J43, J44.0, J47
1002	Asthma	J45
1101	Chronic liver disease except cirrhosis	B18, K70, K71, K73, K74, K76
1102	Hepatic steatosis	K70.0, K75.81, K76.0
1103	Liver cirrhosis	K70.2, K70.30, K70.31, K71.7, K74.0, K74.3, K74.4, K74.5, K74.60, K74.69
1104	Gastroesophageal reflux disease	K21.00, K21.01, K21.9
1105	Regional enteritis and ulcerative colitis	K50, K51
1106	Malabsorption syndrome and food intolerance	E73, E74, K90.0, K90, K91.2
1201	Psoriasis	L40
1202	Atopic dermatitis	L20.0, L20.81, L20.82, L20.83, L20.84, L20.89, L20.9,

		L30.2
1301	Osteoporosis	M80, M81
1302	Osteoarthritis, spondylosis	M15, M16, M17, M18, M19, M47
1303	Gout and other crystal arthropathies	M10, M11, M1A
1304	Other arthropathy	A52.16, E08.6, E09.6, E10.6, E11.6, E13.6, L40.5, M02, M07, M12, M13, M14, M25, M36, M46, M48
1305	Rheumatoid arthritis and related diseases	M05, M06, M08, M12, M45, M48.8
1306	Collagen disease and vasculitis	D68.62, I09.0, I09.2, M30, M31, M32, M33, M34, M35, M36.0, M36.8
1307	Fibromyalgia	M79.7
1401	Chronic kidney disease	D63.1, E08.2, E09.2, E10.2, E11.2, E13.2, I12, I13, I95.3, N18, R88.0, T81.5, T82.4, T85.6, T85.7, T86.1, Z49, Z91.15, Z94.0, Z99.2, V42.0, V45.1, V56
1402	Urinary lithiasis	N20, N21, N22, N23
1999	Amputation	E11.42, I70.261, I70.262, I70.298

B. Demographic

Internal Code	Demographic records
1111	Male (Gender)
2222	Female (Gender)
9000-9120	Age (0-120)

Single Split - Multiple Replicas

Loss curves for Synthetic Generation

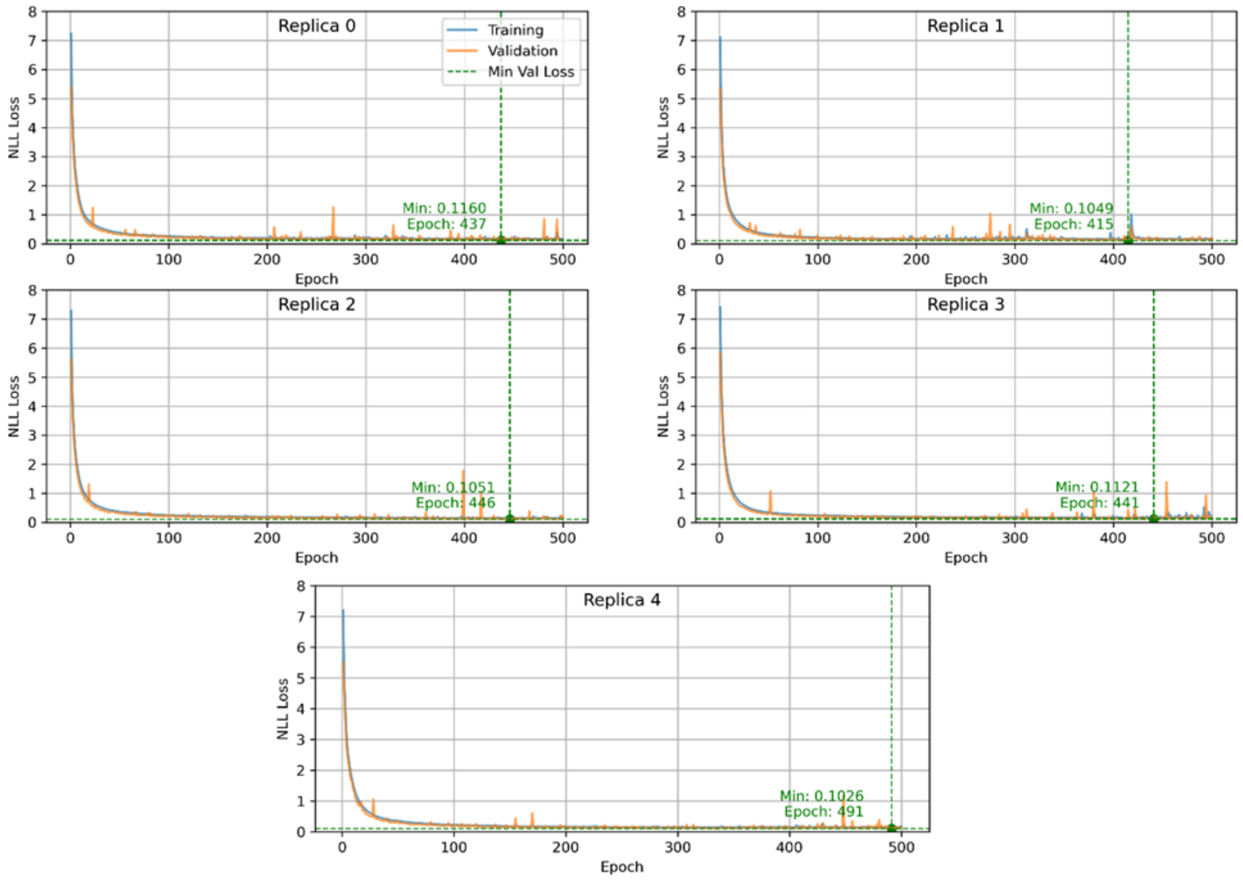

Supplementary Figure 1: Negative log-likelihood (NLL) Loss Curves (SSMR). Training and validation curves were obtained for synthetic generation with a fixed-epoch approach (500 epochs). Although NLL Loss values stabilized around 100-200 epochs, minimal NLL values are found after 400 epochs in all the five replicas.

Raw cohort Descriptive Analysis

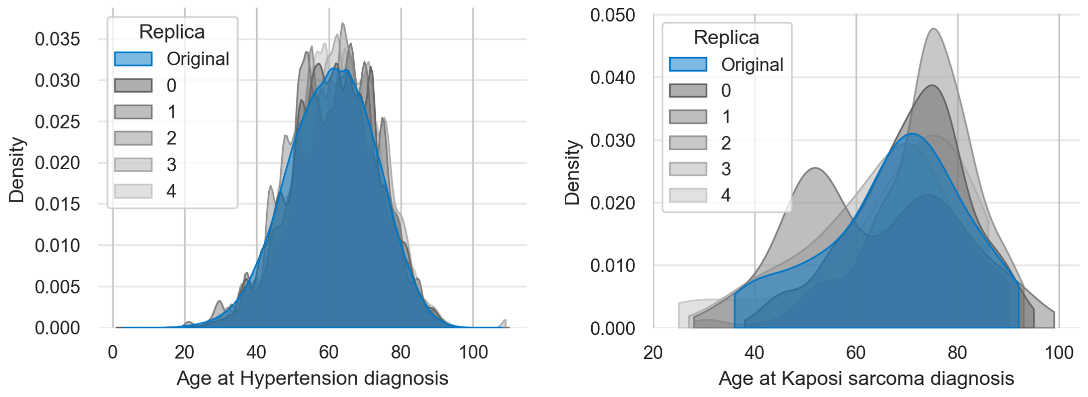

Supplementary Figure 2: Comparison of Age at Diagnosis Distributions (Raw). The density plots compare the distribution of age at diagnosis for a high-prevalence comorbidity (Hypertension, left) and a low-prevalence one (Kaposi's sarcoma, right) between the original data (blue) and five synthetic replicas (grey shades).

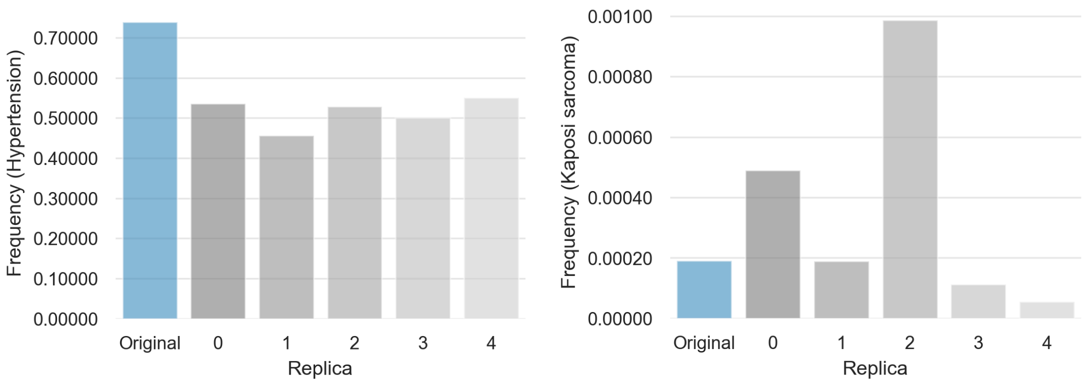

Supplementary Figure 3: Comparison of Categorical Disease Frequencies (Raw). The bar charts compare the frequency of a high-prevalence comorbidity (Hypertension, left) and a low-prevalence one (Kaposi's sarcoma, right). The frequency in the original dataset (blue) is shown alongside the frequencies from five synthetic replicas (grey shades).

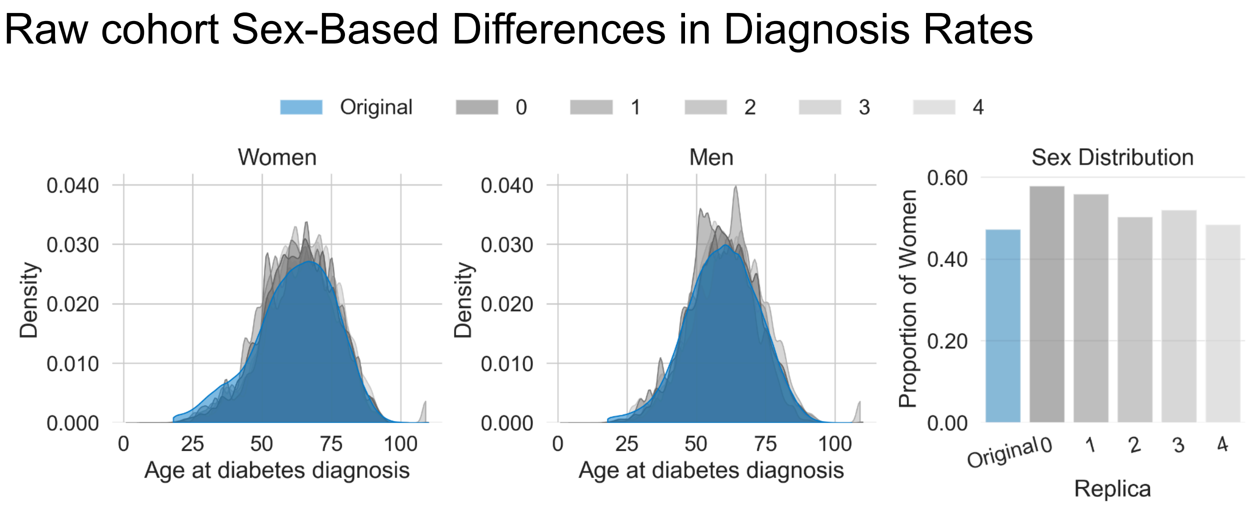

Supplementary Figure 4: Comparison of Age at Diabetes Diagnosis and Sex Distribution (Raw). The density plots compare the age at diagnosis for women (left) and men (right) between the original data (blue) and five synthetic replicas (grey shades). The bar chart (right) shows the proportion of women in the original cohort versus the five replicas, demonstrating the model's ability to capture demographic distributions, although women are generally overrepresented.

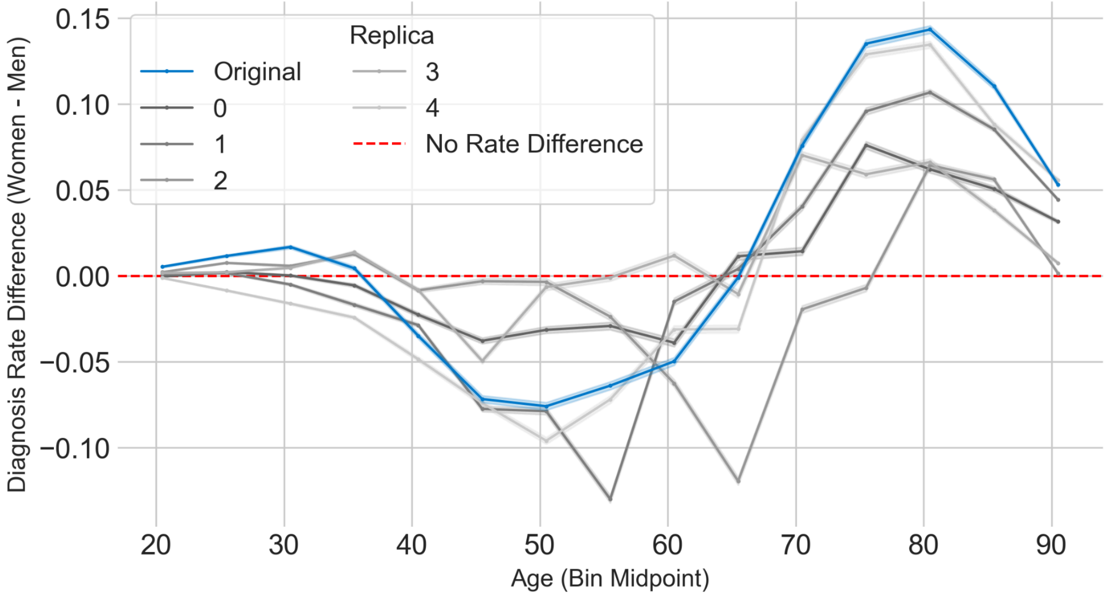

Supplementary Figure 5. Sex-Based Differences in the Rate of Diagnosis Across the Lifespan (Raw). The plot displays the absolute difference in the per-person diagnosis rate (Women - Men) across 5-year age intervals. The trend for the original dataset is shown in blue, with simulated data replicas in grey. The dashed red line at y=0 indicates no difference between the sexes. The results highlight that the diagnosis rate is higher among women in early adulthood and later life, while men exhibit a higher rate during middle age. The synthetic replicas follow the pattern, specially #0. Other replicas show a similar but delayed pattern.

Type	Replica	DTW_Distance
Orig-Rep	4	0,2119451434
Orig-Rep	3	0,3902999385
Orig-Rep	2	0,4264530255
Orig-Rep	1	0,3228595603
Orig-Rep	0	0,3928858674

Supplementary Table 2. DTW distances between the Original and the Replicas (SSMR-Raw). The table lists the calculated Dynamic Time Warping (DTW) distance between the diagnosis rate difference trajectory of the original data and each of the five synthetic replicas for the raw cohort. A lower distance indicates a higher similarity to the original biomedical pattern.

Refined Cohort Comorbidities Prevalence

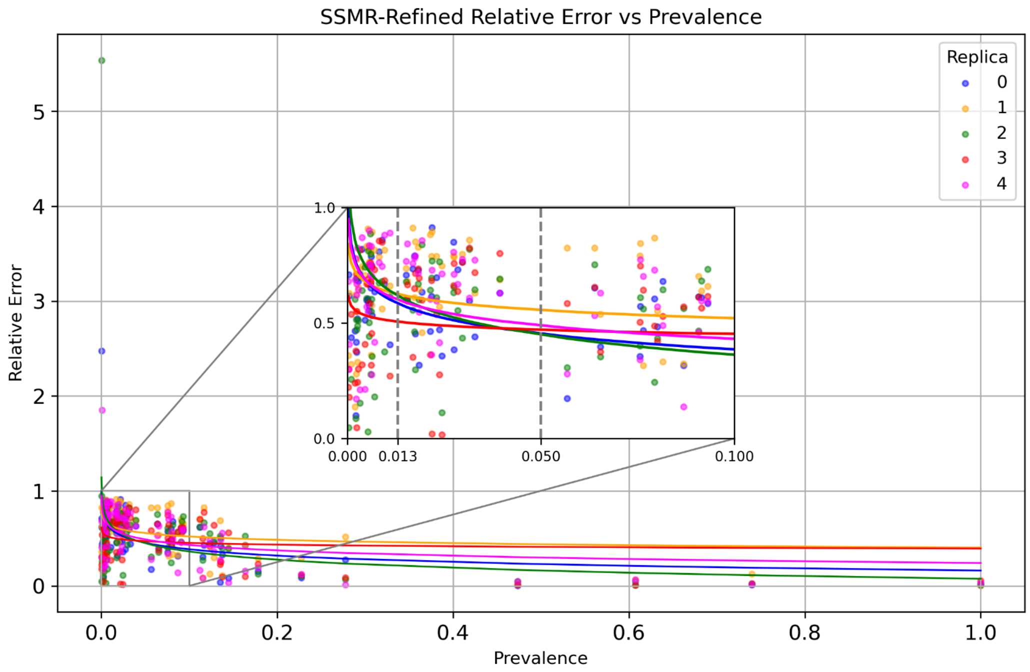

Supplementary Figure 6: Relative error against original prevalence for individual comorbidities (SSMR-Refined). The dashed vertical line at a prevalence of 0.05 indicates the empirically identified threshold below which synthetic generation becomes increasingly sparse and unreliable. A second, less restrictive threshold is also observable at a prevalence of 0.013, suggesting an alternative cutoff at which acceptable fidelity may still be retained. These thresholds provide practical guidance for excluding rare comorbidities, thus improving the overall quality of the synthetic data.

Train-Synthetic, Test-Real performance

Supplementary Table 3. Predictive Performance (AUROC) by Year of Diabetes Diagnosis for Replica 0 (SSMR-Refined).

Diabetes diagnosis year	Metric	Sex	Hybrid	Real	Synthetic
2003	AUROC Mean (SD)	Overall	0.62 (2.44e-04)	0.62 (3.19e-04)	0.62 (7.08e-04)
		Men	0.62 (3.20e-04)	0.62 (3.64e-04)	0.62 (6.47e-04)
		Women	0.63 (3.04e-04)	0.62 (3.90e-04)	0.63 (5.70e-04)
	N negative class	Overall	4544	4544	4544
		Men	2131	2131	2131
		Women	2413	2413	2413
	N positive class	Overall	1474	1474	1474
		Men	765	765	765
		Women	709	709	709
2004	AUROC Mean (SD)	Overall	0.64 (2.31e-04)	0.63 (3.41e-04)	0.63 (5.60e-04)
		Men	0.62 (3.78e-04)	0.62 (4.10e-04)	0.62 (7.26e-04)
		Women	0.65 (2.49e-04)	0.65 (3.78e-04)	0.65 (5.36e-04)
	N negative class	Overall	5390	5390	5390
		Men	2572	2572	2572
		Women	2818	2818	2818
	N positive class	Overall	1425	1425	1425
		Men	739	739	739
		Women	686	686	686
2005	AUROC Mean (SD)	Overall	0.65 (2.20e-04)	0.65 (2.44e-04)	0.64 (7.19e-04)
		Men	0.64 (3.02e-04)	0.64 (3.24e-04)	0.64 (6.89e-04)
		Women	0.66 (2.81e-04)	0.66 (3.61e-04)	0.65 (8.43e-04)
	N negative class	Overall	5166	5166	5166
		Men	2582	2582	2582
		Women	2584	2584	2584
	N positive class	Overall	1330	1330	1330
		Men	718	718	718
		Women	612	612	612
2006	AUROC Mean (SD)	Overall	0.66 (2.60e-04)	0.65 (3.06e-04)	0.65 (7.80e-04)
		Men	0.66 (3.06e-04)	0.66 (3.51e-04)	0.65 (8.35e-04)
		Women	0.66 (3.12e-04)	0.66 (3.94e-04)	0.65 (7.93e-04)
	N negative class	Overall	5682	5682	5682
		Men	2859	2859	2859

		Women	2823	2823	2823
	N positive class	Overall	1252	1252	1252
		Men	705	705	705
		Women	547	547	547
2007	AUROC Mean (SD)	Overall	0.67 (2.68e-04)	0.67 (2.70e-04)	0.66 (7.50e-04)
		Men	0.68 (2.64e-04)	0.68 (2.89e-04)	0.67 (7.04e-04)
		Women	0.67 (3.35e-04)	0.66 (4.06e-04)	0.66 (8.12e-04)
	N negative class	Overall	7016	7016	7016
		Men	3612	3612	3612
		Women	3404	3404	3404
	N positive class	Overall	1460	1460	1460
		Men	791	791	791
		Women	669	669	669
2008	AUROC Mean (SD)	Overall	0.66 (3.10e-04)	0.67 (3.29e-04)	0.65 (9.60e-04)
		Men	0.65 (2.86e-04)	0.65 (3.88e-04)	0.64 (8.50e-04)
		Women	0.68 (4.80e-04)	0.68 (4.59e-04)	0.66 (1.09e-03)
	N negative class	Overall	7361	7361	7361
		Men	3867	3867	3867
		Women	3494	3494	3494
	N positive class	Overall	1292	1292	1292
		Men	702	702	702
		Women	590	590	590
2009	AUROC Mean (SD)	Overall	0.69 (2.83e-04)	0.69 (2.95e-04)	0.68 (8.17e-04)
		Men	0.69 (3.11e-04)	0.69 (3.37e-04)	0.68 (7.16e-04)
		Women	0.7 (3.84e-04)	0.7 (3.77e-04)	0.68 (9.64e-04)
	N negative class	Overall	7414	7414	7414
		Men	4052	4052	4052
		Women	3362	3362	3362
	N positive class	Overall	1136	1136	1136
		Men	658	658	658
		Women	478	478	478
2010	AUROC Mean (SD)	Overall	0.69 (2.98e-04)	0.69 (4.00e-04)	0.68 (8.66e-04)
		Men	0.7 (3.04e-04)	0.7 (3.96e-04)	0.69 (6.86e-04)
		Women	0.67 (4.04e-04)	0.67 (4.63e-04)	0.66 (9.82e-04)
	N negative class	Overall	7663	7663	7663
		Men	4094	4094	4094
		Women	3569	3569	3569

	N positive class	Overall	1100	1100	1100
		Men	641	641	641
		Women	459	459	459
2011	AUROC Mean (SD)	Overall	0.7 (3.07e-04)	0.7 (3.34e-04)	0.68 (9.78e-04)
		Men	0.7 (3.03e-04)	0.7 (3.11e-04)	0.69 (8.53e-04)
		Women	0.7 (5.14e-04)	0.7 (5.71e-04)	0.7 (1.16e-03)
	N negative class	Overall	7444	7444	7444
		Men	4075	4075	4075
		Women	3369	3369	3369
	N positive class	Overall	943	943	943
		Men	511	511	511
		Women	432	432	432
2012	AUROC Mean (SD)	Overall	0.71 (3.25e-04)	0.71 (3.73e-04)	0.69 (9.47e-04)
		Men	0.7 (3.64e-04)	0.7 (4.08e-04)	0.68 (1.11e-03)
		Women	0.73 (4.45e-04)	0.74 (4.98e-04)	0.71 (1.12e-03)
	N negative class	Overall	6730	6730	6730
		Men	3661	3661	3661
		Women	3069	3069	3069
	N positive class	Overall	715	715	715
		Men	432	432	432
		Women	283	283	283
2013	AUROC Mean (SD)	Overall	0.73 (3.12e-04)	0.73 (3.51e-04)	0.71 (9.02e-04)
		Men	0.72 (3.38e-04)	0.72 (3.73e-04)	0.7 (1.01e-03)
		Women	0.75 (5.44e-04)	0.75 (5.73e-04)	0.72 (1.37e-03)
	N negative class	Overall	7103	7103	7103
		Men	3809	3809	3809
		Women	3294	3294	3294
	N positive class	Overall	703	703	703
		Men	421	421	421
		Women	282	282	282
2014	AUROC Mean (SD)	Overall	0.74 (3.35e-04)	0.74 (4.04e-04)	0.71 (9.66e-04)
		Men	0.73 (3.17e-04)	0.72 (4.00e-04)	0.71 (1.03e-03)
		Women	0.76 (5.11e-04)	0.76 (5.56e-04)	0.72 (1.18e-03)
	N negative class	Overall	7011	7011	7011
		Men	3649	3649	3649
		Women	3362	3362	3362
	N positive class	Overall	548	548	548

		Men	307	307	307
		Women	241	241	241
2015	AUROC Mean (SD)	Overall	0.74 (3.91e-04)	0.74 (4.94e-04)	0.71 (1.17e-03)
		Men	0.75 (4.57e-04)	0.75 (6.07e-04)	0.71 (1.40e-03)
		Women	0.73 (5.85e-04)	0.73 (6.35e-04)	0.71 (1.33e-03)
	N negative class	Overall	6457	6457	6457
		Men	3426	3426	3426
		Women	3031	3031	3031
	N positive class	Overall	437	437	437
		Men	246	246	246
		Women	191	191	191
2016	AUROC Mean (SD)	Overall	0.75 (4.38e-04)	0.75 (3.96e-04)	0.7 (1.54e-03)
		Men	0.71 (5.36e-04)	0.72 (4.78e-04)	0.68 (2.01e-03)
		Women	0.79 (5.27e-04)	0.8 (5.79e-04)	0.75 (1.65e-03)
	N negative class	Overall	6561	6561	6561
		Men	3561	3561	3561
		Women	3000	3000	3000
	N positive class	Overall	383	383	383
		Men	205	205	205
		Women	178	178	178
2017	AUROC Mean (SD)	Overall	0.73 (3.75e-04)	0.73 (3.86e-04)	0.7 (1.12e-03)
		Men	0.71 (5.01e-04)	0.72 (5.38e-04)	0.69 (1.37e-03)
		Women	0.75 (5.58e-04)	0.75 (5.68e-04)	0.73 (1.47e-03)
	N negative class	Overall	6820	6820	6820
		Men	3643	3643	3643
		Women	3177	3177	3177
	N positive class	Overall	297	297	297
		Men	162	162	162
		Women	135	135	135
2018	AUROC Mean (SD)	Overall	0.74 (5.59e-04)	0.75 (6.12e-04)	0.7 (1.63e-03)
		Men	0.73 (6.86e-04)	0.74 (7.58e-04)	0.7 (1.71e-03)
		Women	0.76 (6.08e-04)	0.77 (6.56e-04)	0.72 (2.13e-03)
	N negative class	Overall	6951	6951	6951
		Men	3798	3798	3798
		Women	3153	3153	3153
	N positive class	Overall	277	277	277
		Men	156	156	156

		Women	121	121	121
2019	AUROC Mean (SD)	Overall	0.71 (4.94e-04)	0.72 (5.24e-04)	0.68 (1.53e-03)
		Men	0.72 (5.75e-04)	0.72 (6.23e-04)	0.7 (2.10e-03)
		Women	0.71 (8.25e-04)	0.72 (8.22e-04)	0.67 (2.01e-03)
	N negative class	Overall	7318	7318	7318
		Men	4010	4010	4010
		Women	3308	3308	3308
	N positive class	Overall	226	226	226
		Men	125	125	125
		Women	101	101	101
2020	AUROC Mean (SD)	Overall	0.76 (6.72e-04)	0.77 (8.07e-04)	0.72 (1.56e-03)
		Men	0.77 (7.13e-04)	0.78 (7.45e-04)	0.74 (1.84e-03)
		Women	0.75 (1.16e-03)	0.76 (1.27e-03)	0.72 (2.43e-03)
	N negative class	Overall	6464	6464	6464
		Men	3409	3409	3409
		Women	3055	3055	3055
	N positive class	Overall	152	152	152
		Men	83	83	83
		Women	69	69	69
2021	AUROC Mean (SD)	Overall	0.68 (6.16e-04)	0.68 (8.05e-04)	0.67 (1.69e-03)
		Men	0.7 (6.23e-04)	0.7 (8.76e-04)	0.68 (1.90e-03)
		Women	0.66 (1.02e-03)	0.66 (1.24e-03)	0.67 (2.44e-03)
	N negative class	Overall	9712	9712	9712
		Men	4998	4998	4998
		Women	4714	4714	4714
	N positive class	Overall	109	109	109
		Men	62	62	62
		Women	47	47	47
2022	AUROC Mean (SD)	Overall	0.72 (1.11e-03)	0.71 (1.31e-03)	0.66 (3.69e-03)
		Men	0.68 (1.39e-03)	0.66 (1.39e-03)	0.65 (4.13e-03)
		Women	0.78 (1.92e-03)	0.77 (2.35e-03)	0.67 (6.91e-03)
	N negative class	Overall	6511	6511	6511
		Men	3464	3464	3464
		Women	3047	3047	3047
	N positive class	Overall	34	34	34
		Men	19	19	19
		Women	15	15	15

Supplementary Table 4. Predictive Performance (AUROC) by Year of Diabetes Diagnosis for Replica 1 (SSMR-Refined).

betes diagnosis y	Metric	Sex	Hybrid	Real	Synthetic
2003	AUROC Mean (SD)	Overall	0.62 (2.38e-04)	0.62 (3.19e-04)	0.62 (8.32e-04)
		Men	0.63 (4.34e-04)	0.62 (3.64e-04)	0.63 (1.05e-03)
		Women	0.63 (3.39e-04)	0.62 (3.90e-04)	0.62 (9.59e-04)
	N negative class	Overall	4544	4544	4544
		Men	2131	2131	2131
		Women	2413	2413	2413
	N positive class	Overall	1474	1474	1474
		Men	765	765	765
		Women	709	709	709
2004	AUROC Mean (SD)	Overall	0.64 (3.22e-04)	0.63 (3.41e-04)	0.63 (9.44e-04)
		Men	0.62 (7.55e-04)	0.62 (4.10e-04)	0.61 (1.27e-03)
		Women	0.65 (3.06e-04)	0.65 (3.78e-04)	0.64 (9.68e-04)
	N negative class	Overall	5390	5390	5390
		Men	2572	2572	2572
		Women	2818	2818	2818
	N positive class	Overall	1425	1425	1425
		Men	739	739	739
		Women	686	686	686
2005	AUROC Mean (SD)	Overall	0.65 (2.02e-04)	0.65 (2.44e-04)	0.64 (8.16e-04)
		Men	0.64 (4.08e-04)	0.64 (3.24e-04)	0.65 (1.07e-03)
		Women	0.65 (2.87e-04)	0.66 (3.61e-04)	0.64 (9.79e-04)
	N negative class	Overall	5166	5166	5166
		Men	2582	2582	2582
		Women	2584	2584	2584
	N positive class	Overall	1330	1330	1330
		Men	718	718	718
		Women	612	612	612
2006	AUROC Mean (SD)	Overall	0.66 (2.89e-04)	0.65 (3.06e-04)	0.65 (7.92e-04)
		Men	0.66 (3.38e-04)	0.66 (3.51e-04)	0.66 (7.93e-04)
		Women	0.66 (4.73e-04)	0.66 (3.94e-04)	0.65 (1.13e-03)
	N negative class	Overall	5682	5682	5682
		Men	2859	2859	2859

		Women	2823	2823	2823
	N positive class	Overall	1252	1252	1252
		Men	705	705	705
		Women	547	547	547
2007	AUROC Mean (SD)	Overall	0.68 (2.21e-04)	0.67 (2.70e-04)	0.67 (7.81e-04)
		Men	0.68 (3.19e-04)	0.68 (2.89e-04)	0.68 (1.04e-03)
		Women	0.67 (3.26e-04)	0.66 (4.06e-04)	0.65 (9.13e-04)
	N negative class	Overall	7016	7016	7016
		Men	3612	3612	3612
		Women	3404	3404	3404
	N positive class	Overall	1460	1460	1460
		Men	791	791	791
		Women	669	669	669
2008	AUROC Mean (SD)	Overall	0.67 (2.68e-04)	0.67 (3.29e-04)	0.65 (9.63e-04)
		Men	0.66 (3.14e-04)	0.65 (3.88e-04)	0.65 (1.08e-03)
		Women	0.68 (4.57e-04)	0.68 (4.59e-04)	0.64 (1.27e-03)
	N negative class	Overall	7361	7361	7361
		Men	3867	3867	3867
		Women	3494	3494	3494
	N positive class	Overall	1292	1292	1292
		Men	702	702	702
		Women	590	590	590
2009	AUROC Mean (SD)	Overall	0.69 (2.70e-04)	0.69 (2.95e-04)	0.67 (1.06e-03)
		Men	0.69 (3.25e-04)	0.69 (3.37e-04)	0.68 (1.16e-03)
		Women	0.7 (3.96e-04)	0.7 (3.77e-04)	0.67 (1.38e-03)
	N negative class	Overall	7414	7414	7414
		Men	4052	4052	4052
		Women	3362	3362	3362
	N positive class	Overall	1136	1136	1136
		Men	658	658	658
		Women	478	478	478
2010	AUROC Mean (SD)	Overall	0.69 (2.84e-04)	0.69 (4.00e-04)	0.66 (1.09e-03)
		Men	0.7 (3.52e-04)	0.7 (3.96e-04)	0.69 (1.15e-03)
		Women	0.67 (4.33e-04)	0.67 (4.63e-04)	0.64 (1.47e-03)
	N negative class	Overall	7663	7663	7663
		Men	4094	4094	4094
		Women	3569	3569	3569

	N positive class	Overall	1100	1100	1100
		Men	641	641	641
		Women	459	459	459
2011	AUROC Mean (SD)	Overall	0.7 (3.10e-04)	0.7 (3.34e-04)	0.67 (1.01e-03)
		Men	0.7 (4.06e-04)	0.7 (3.11e-04)	0.67 (9.20e-04)
		Women	0.7 (5.00e-04)	0.7 (5.71e-04)	0.67 (1.37e-03)
	N negative class	Overall	7444	7444	7444
		Men	4075	4075	4075
		Women	3369	3369	3369
	N positive class	Overall	943	943	943
		Men	511	511	511
		Women	432	432	432
2012	AUROC Mean (SD)	Overall	0.71 (3.43e-04)	0.71 (3.73e-04)	0.68 (9.64e-04)
		Men	0.7 (3.79e-04)	0.7 (4.08e-04)	0.67 (1.11e-03)
		Women	0.73 (4.66e-04)	0.74 (4.98e-04)	0.69 (1.28e-03)
	N negative class	Overall	6730	6730	6730
		Men	3661	3661	3661
		Women	3069	3069	3069
	N positive class	Overall	715	715	715
		Men	432	432	432
		Women	283	283	283
2013	AUROC Mean (SD)	Overall	0.73 (3.56e-04)	0.73 (3.51e-04)	0.68 (1.24e-03)
		Men	0.72 (4.13e-04)	0.72 (3.73e-04)	0.68 (1.21e-03)
		Women	0.74 (5.43e-04)	0.75 (5.73e-04)	0.68 (1.69e-03)
	N negative class	Overall	7103	7103	7103
		Men	3809	3809	3809
		Women	3294	3294	3294
	N positive class	Overall	703	703	703
		Men	421	421	421
		Women	282	282	282
2014	AUROC Mean (SD)	Overall	0.74 (3.23e-04)	0.74 (4.04e-04)	0.7 (1.24e-03)
		Men	0.72 (2.93e-04)	0.72 (4.00e-04)	0.68 (1.30e-03)
		Women	0.76 (5.09e-04)	0.76 (5.56e-04)	0.72 (1.58e-03)
	N negative class	Overall	7011	7011	7011
		Men	3649	3649	3649
		Women	3362	3362	3362
	N positive class	Overall	548	548	548

		Men	307	307	307
		Women	241	241	241
2015	AUROC Mean (SD)	Overall	0.73 (4.67e-04)	0.74 (4.94e-04)	0.68 (1.53e-03)
		Men	0.74 (5.51e-04)	0.75 (6.07e-04)	0.68 (1.66e-03)
		Women	0.72 (5.82e-04)	0.73 (6.35e-04)	0.68 (1.58e-03)
	N negative class	Overall	6457	6457	6457
		Men	3426	3426	3426
		Women	3031	3031	3031
	N positive class	Overall	437	437	437
		Men	246	246	246
		Women	191	191	191
2016	AUROC Mean (SD)	Overall	0.75 (3.36e-04)	0.75 (3.96e-04)	0.71 (1.25e-03)
		Men	0.72 (4.39e-04)	0.72 (4.78e-04)	0.69 (1.08e-03)
		Women	0.79 (5.11e-04)	0.8 (5.79e-04)	0.73 (2.05e-03)
	N negative class	Overall	6561	6561	6561
		Men	3561	3561	3561
		Women	3000	3000	3000
	N positive class	Overall	383	383	383
		Men	205	205	205
		Women	178	178	178
2017	AUROC Mean (SD)	Overall	0.73 (3.74e-04)	0.73 (3.86e-04)	0.67 (1.77e-03)
		Men	0.71 (5.39e-04)	0.72 (5.38e-04)	0.66 (1.56e-03)
		Women	0.75 (4.99e-04)	0.75 (5.68e-04)	0.69 (2.47e-03)
	N negative class	Overall	6820	6820	6820
		Men	3643	3643	3643
		Women	3177	3177	3177
	N positive class	Overall	297	297	297
		Men	162	162	162
		Women	135	135	135
2018	AUROC Mean (SD)	Overall	0.75 (6.18e-04)	0.75 (6.12e-04)	0.67 (2.23e-03)
		Men	0.74 (7.64e-04)	0.74 (7.58e-04)	0.67 (2.18e-03)
		Women	0.76 (6.57e-04)	0.77 (6.56e-04)	0.68 (2.67e-03)
	N negative class	Overall	6951	6951	6951
		Men	3798	3798	3798
		Women	3153	3153	3153
	N positive class	Overall	277	277	277
		Men	156	156	156

		Women	121	121	121
2019	AUROC Mean (SD)	Overall	0.72 (4.53e-04)	0.72 (5.24e-04)	0.66 (1.82e-03)
		Men	0.73 (6.41e-04)	0.72 (6.23e-04)	0.68 (2.10e-03)
		Women	0.7 (7.53e-04)	0.72 (8.22e-04)	0.63 (2.43e-03)
	N negative class	Overall	7318	7318	7318
		Men	4010	4010	4010
		Women	3308	3308	3308
	N positive class	Overall	226	226	226
		Men	125	125	125
		Women	101	101	101
2020	AUROC Mean (SD)	Overall	0.77 (7.25e-04)	0.77 (8.07e-04)	0.71 (1.84e-03)
		Men	0.78 (8.19e-04)	0.78 (7.45e-04)	0.73 (2.01e-03)
		Women	0.76 (1.07e-03)	0.76 (1.27e-03)	0.68 (3.03e-03)
	N negative class	Overall	6464	6464	6464
		Men	3409	3409	3409
		Women	3055	3055	3055
	N positive class	Overall	152	152	152
		Men	83	83	83
		Women	69	69	69
2021	AUROC Mean (SD)	Overall	0.68 (6.70e-04)	0.68 (8.05e-04)	0.63 (2.30e-03)
		Men	0.69 (7.97e-04)	0.7 (8.76e-04)	0.65 (2.12e-03)
		Women	0.65 (1.10e-03)	0.66 (1.24e-03)	0.6 (3.59e-03)
	N negative class	Overall	9712	9712	9712
		Men	4998	4998	4998
		Women	4714	4714	4714
	N positive class	Overall	109	109	109
		Men	62	62	62
		Women	47	47	47
2022	AUROC Mean (SD)	Overall	0.69 (1.44e-03)	0.71 (1.31e-03)	0.62 (3.46e-03)
		Men	0.67 (1.28e-03)	0.66 (1.39e-03)	0.64 (2.78e-03)
		Women	0.72 (2.83e-03)	0.77 (2.35e-03)	0.59 (6.45e-03)
	N negative class	Overall	6511	6511	6511
		Men	3464	3464	3464
		Women	3047	3047	3047
	N positive class	Overall	34	34	34
		Men	19	19	19
		Women	15	15	15

Supplementary Table 5. Predictive Performance (AUROC) by Year of Diabetes Diagnosis for Replica 2 (SSMR-Refined).

Diabetes diagnosis year	Metric	Sex	Hybrid	Real	Synthetic
2003	AUROC Mean (SD)	Overall	0.62 (3.13e-04)	0.62 (3.19e-04)	
		Men	0.62 (3.92e-04)	0.62 (3.64e-04)	0.62 (6.57e-04)
		Women	0.63 (4.34e-04)	0.62 (3.90e-04)	0.61 (9.06e-04)
	N negative class	Overall	4544	4544	
		Men	2131	2131	2131
		Women	2413	2413	2413
	N positive class	Overall	1474	1474	
		Men	765	765	765
		Women	709	709	709
2004	AUROC Mean (SD)	Overall	0.64 (2.85e-04)	0.63 (3.41e-04)	0.63 (7.01e-04)
		Men	0.62 (4.51e-04)	0.62 (4.10e-04)	0.62 (8.00e-04)
		Women	0.65 (3.86e-04)	0.65 (3.78e-04)	0.64 (9.46e-04)
	N negative class	Overall	5390	5390	5390
		Men	2572	2572	2572
		Women	2818	2818	2818
	N positive class	Overall	1425	1425	1425
		Men	739	739	739
		Women	686	686	686
2005	AUROC Mean (SD)	Overall	0.64 (3.07e-04)	0.65 (2.44e-04)	0.64 (7.72e-04)
		Men	0.64 (4.32e-04)	0.64 (3.24e-04)	0.63 (8.86e-04)
		Women	0.65 (3.96e-04)	0.66 (3.61e-04)	0.64 (1.06e-03)
	N negative class	Overall	5166	5166	5166
		Men	2582	2582	2582
		Women	2584	2584	2584
	N positive class	Overall	1330	1330	1330
		Men	718	718	718
		Women	612	612	612
2006	AUROC Mean (SD)	Overall	0.66 (2.80e-04)	0.65 (3.06e-04)	0.65 (7.73e-04)
		Men	0.66 (3.45e-04)	0.66 (3.51e-04)	0.65 (7.01e-04)
		Women	0.66 (3.88e-04)	0.66 (3.94e-04)	0.64 (1.10e-03)
	N negative class	Overall	5682	5682	5682
		Men	2859	2859	2859
		Women	2823	2823	2823

	N positive class	Overall	1252	1252	1252
		Men	705	705	705
		Women	547	547	547
2007	AUROC Mean (SD)	Overall	0.67 (3.06e-04)	0.67 (2.70e-04)	0.66 (8.10e-04)
		Men	0.68 (3.50e-04)	0.68 (2.89e-04)	0.67 (7.51e-04)
		Women	0.66 (4.41e-04)	0.66 (4.06e-04)	0.65 (9.71e-04)
	N negative class	Overall	7016	7016	7016
		Men	3612	3612	3612
		Women	3404	3404	3404
	N positive class	Overall	1460	1460	1460
		Men	791	791	791
		Women	669	669	669
2008	AUROC Mean (SD)	Overall	0.66 (3.14e-04)	0.67 (3.29e-04)	0.64 (9.74e-04)
		Men	0.65 (3.62e-04)	0.65 (3.88e-04)	0.64 (8.38e-04)
		Women	0.68 (4.69e-04)	0.68 (4.59e-04)	0.65 (1.34e-03)
	N negative class	Overall	7361	7361	7361
		Men	3867	3867	3867
		Women	3494	3494	3494
	N positive class	Overall	1292	1292	1292
		Men	702	702	702
		Women	590	590	590
2009	AUROC Mean (SD)	Overall	0.69 (2.82e-04)	0.69 (2.95e-04)	0.67 (8.73e-04)
		Men	0.69 (3.40e-04)	0.69 (3.37e-04)	0.67 (8.19e-04)
		Women	0.69 (4.04e-04)	0.7 (3.77e-04)	0.66 (1.22e-03)
	N negative class	Overall	7414	7414	7414
		Men	4052	4052	4052
		Women	3362	3362	3362
	N positive class	Overall	1136	1136	1136
		Men	658	658	658
		Women	478	478	478
2010	AUROC Mean (SD)	Overall	0.69 (3.20e-04)	0.69 (4.00e-04)	0.67 (9.97e-04)
		Men	0.7 (3.79e-04)	0.7 (3.96e-04)	0.68 (1.01e-03)
		Women	0.67 (4.14e-04)	0.67 (4.63e-04)	0.65 (1.30e-03)
	N negative class	Overall	7663	7663	7663
		Men	4094	4094	4094
		Women	3569	3569	3569
	N positive class	Overall	1100	1100	1100

		Men	641	641	641
		Women	459	459	459
2011	AUROC Mean (SD)	Overall	0.7 (3.76e-04)	0.7 (3.34e-04)	0.67 (1.03e-03)
		Men	0.7 (3.67e-04)	0.7 (3.11e-04)	0.67 (1.01e-03)
		Women	0.7 (5.90e-04)	0.7 (5.71e-04)	0.67 (1.37e-03)
	N negative class	Overall	7444	7444	7444
		Men	4075	4075	4075
		Women	3369	3369	3369
	N positive class	Overall	943	943	943
		Men	511	511	511
		Women	432	432	432
2012	AUROC Mean (SD)	Overall	0.71 (3.54e-04)	0.71 (3.73e-04)	0.68 (1.04e-03)
		Men	0.69 (4.17e-04)	0.7 (4.08e-04)	0.67 (1.22e-03)
		Women	0.73 (4.59e-04)	0.74 (4.98e-04)	0.71 (1.43e-03)
	N negative class	Overall	6730	6730	6730
		Men	3661	3661	3661
		Women	3069	3069	3069
	N positive class	Overall	715	715	715
		Men	432	432	432
		Women	283	283	283
2013	AUROC Mean (SD)	Overall	0.73 (3.34e-04)	0.73 (3.51e-04)	0.7 (9.83e-04)
		Men	0.72 (3.39e-04)	0.72 (3.73e-04)	0.7 (1.10e-03)
		Women	0.75 (5.62e-04)	0.75 (5.73e-04)	0.7 (1.40e-03)
	N negative class	Overall	7103	7103	7103
		Men	3809	3809	3809
		Women	3294	3294	3294
	N positive class	Overall	703	703	703
		Men	421	421	421
		Women	282	282	282
2014	AUROC Mean (SD)	Overall	0.74 (3.53e-04)	0.74 (4.04e-04)	0.7 (1.16e-03)
		Men	0.73 (3.36e-04)	0.72 (4.00e-04)	0.69 (1.16e-03)
		Women	0.75 (5.40e-04)	0.76 (5.56e-04)	0.72 (1.47e-03)
	N negative class	Overall	7011	7011	7011
		Men	3649	3649	3649
		Women	3362	3362	3362
	N positive class	Overall	548	548	548
		Men	307	307	307

		Women	241	241	241
2015	AUROC Mean (SD)	Overall	0.74 (4.12e-04)	0.74 (4.94e-04)	0.71 (1.13e-03)
		Men	0.75 (4.45e-04)	0.75 (6.07e-04)	0.72 (1.31e-03)
		Women	0.72 (5.27e-04)	0.73 (6.35e-04)	0.7 (1.40e-03)
	N negative class	Overall	6457	6457	6457
		Men	3426	3426	3426
		Women	3031	3031	3031
	N positive class	Overall	437	437	437
		Men	246	246	246
		Women	191	191	191
2016	AUROC Mean (SD)	Overall	0.75 (4.96e-04)	0.75 (3.96e-04)	0.7 (1.51e-03)
		Men	0.71 (6.31e-04)	0.72 (4.78e-04)	0.69 (1.25e-03)
		Women	0.79 (5.73e-04)	0.8 (5.79e-04)	0.73 (2.53e-03)
	N negative class	Overall	6561	6561	6561
		Men	3561	3561	3561
		Women	3000	3000	3000
	N positive class	Overall	383	383	383
		Men	205	205	205
		Women	178	178	178
2017	AUROC Mean (SD)	Overall	0.73 (3.78e-04)	0.73 (3.86e-04)	0.69 (1.35e-03)
		Men	0.72 (4.49e-04)	0.72 (5.38e-04)	0.68 (1.49e-03)
		Women	0.75 (5.16e-04)	0.75 (5.68e-04)	0.71 (1.71e-03)
	N negative class	Overall	6820	6820	6820
		Men	3643	3643	3643
		Women	3177	3177	3177
	N positive class	Overall	297	297	297
		Men	162	162	162
		Women	135	135	135
2018	AUROC Mean (SD)	Overall	0.75 (5.63e-04)	0.75 (6.12e-04)	0.68 (1.99e-03)
		Men	0.74 (7.22e-04)	0.74 (7.58e-04)	0.69 (1.87e-03)
		Women	0.76 (6.37e-04)	0.77 (6.56e-04)	0.69 (2.64e-03)
	N negative class	Overall	6951	6951	6951
		Men	3798	3798	3798
		Women	3153	3153	3153
	N positive class	Overall	277	277	277
		Men	156	156	156
		Women	121	121	121

2019	AUROC Mean (SD)	Overall	0.71 (4.86e-04)	0.72 (5.24e-04)	0.66 (1.52e-03)
		Men	0.71 (6.47e-04)	0.72 (6.23e-04)	0.68 (1.69e-03)
		Women	0.71 (8.62e-04)	0.72 (8.22e-04)	0.65 (2.38e-03)
	N negative class	Overall	7318	7318	7318
		Men	4010	4010	4010
		Women	3308	3308	3308
	N positive class	Overall	226	226	226
		Men	125	125	125
		Women	101	101	101
2020	AUROC Mean (SD)	Overall	0.77 (6.41e-04)	0.77 (8.07e-04)	0.7 (1.97e-03)
		Men	0.77 (7.38e-04)	0.78 (7.45e-04)	0.7 (2.65e-03)
		Women	0.76 (1.04e-03)	0.76 (1.27e-03)	0.7 (2.67e-03)
	N negative class	Overall	6464	6464	6464
		Men	3409	3409	3409
		Women	3055	3055	3055
	N positive class	Overall	152	152	152
		Men	83	83	83
		Women	69	69	69
2021	AUROC Mean (SD)	Overall	0.67 (6.17e-04)	0.68 (8.05e-04)	0.63 (1.82e-03)
		Men	0.69 (7.88e-04)	0.7 (8.76e-04)	0.64 (2.20e-03)
		Women	0.65 (9.03e-04)	0.66 (1.24e-03)	0.62 (2.87e-03)
	N negative class	Overall	9712	9712	9712
		Men	4998	4998	4998
		Women	4714	4714	4714
	N positive class	Overall	109	109	109
		Men	62	62	62
		Women	47	47	47
2022	AUROC Mean (SD)	Overall	0.72 (1.14e-03)	0.71 (1.31e-03)	0.7 (2.95e-03)
		Men	0.69 (1.45e-03)	0.66 (1.39e-03)	0.73 (1.74e-03)
		Women	0.75 (1.96e-03)	0.77 (2.35e-03)	0.66 (6.17e-03)
	N negative class	Overall	6511	6511	6511
		Men	3464	3464	3464
		Women	3047	3047	3047
	N positive class	Overall	34	34	34
		Men	19	19	19
		Women	15	15	15

Supplementary Table 6. Predictive Performance (AUROC) by Year of Diabetes Diagnosis for Replica 3 (SSMR-Refined).

Diabetes diagnosis year	Metric	Sex	Hybrid	Real	Synthetic
2003	AUROC Mean (SD)	Overall	0.62 (3.07e-04)	0.62 (3.19e-04)	0.62 (5.07e-04)
		Men	0.62 (4.74e-04)	0.62 (3.64e-04)	0.62 (5.90e-04)
		Women	0.63 (2.93e-04)	0.62 (3.90e-04)	0.63 (5.96e-04)
	N negative class	Overall	4544	4544	4544
		Men	2131	2131	2131
		Women	2413	2413	2413
	N positive class	Overall	1474	1474	1474
		Men	765	765	765
		Women	709	709	709
2004	AUROC Mean (SD)	Overall	0.63 (3.01e-04)	0.63 (3.41e-04)	0.63 (4.88e-04)
		Men	0.62 (5.30e-04)	0.62 (4.10e-04)	0.61 (6.32e-04)
		Women	0.65 (3.45e-04)	0.65 (3.78e-04)	0.65 (5.89e-04)
	N negative class	Overall	5390	5390	5390
		Men	2572	2572	2572
		Women	2818	2818	2818
	N positive class	Overall	1425	1425	1425
		Men	739	739	739
		Women	686	686	686
2005	AUROC Mean (SD)	Overall	0.64 (2.33e-04)	0.65 (2.44e-04)	0.63 (4.89e-04)
		Men	0.64 (3.39e-04)	0.64 (3.24e-04)	0.63 (6.18e-04)
		Women	0.65 (3.23e-04)	0.66 (3.61e-04)	0.64 (6.68e-04)
	N negative class	Overall	5166	5166	5166
		Men	2582	2582	2582
		Women	2584	2584	2584
	N positive class	Overall	1330	1330	1330
		Men	718	718	718
		Women	612	612	612
2006	AUROC Mean (SD)	Overall	0.65 (3.08e-04)	0.65 (3.06e-04)	0.64 (5.94e-04)
		Men	0.65 (3.21e-04)	0.66 (3.51e-04)	0.64 (6.22e-04)
		Women	0.66 (3.79e-04)	0.66 (3.94e-04)	0.65 (7.49e-04)
	N negative class	Overall	5682	5682	5682
		Men	2859	2859	2859

		Women	2823	2823	2823
	N positive class	Overall	1252	1252	1252
		Men	705	705	705
		Women	547	547	547
2007	AUROC Mean (SD)	Overall	0.67 (2.44e-04)	0.67 (2.70e-04)	0.66 (5.19e-04)
		Men	0.67 (3.00e-04)	0.68 (2.89e-04)	0.66 (6.76e-04)
		Women	0.67 (3.01e-04)	0.66 (4.06e-04)	0.66 (5.66e-04)
	N negative class	Overall	7016	7016	7016
		Men	3612	3612	3612
		Women	3404	3404	3404
	N positive class	Overall	1460	1460	1460
		Men	791	791	791
		Women	669	669	669
2008	AUROC Mean (SD)	Overall	0.66 (2.57e-04)	0.67 (3.29e-04)	0.65 (6.81e-04)
		Men	0.65 (2.66e-04)	0.65 (3.88e-04)	0.65 (5.98e-04)
		Women	0.68 (3.67e-04)	0.68 (4.59e-04)	0.66 (9.66e-04)
	N negative class	Overall	7361	7361	7361
		Men	3867	3867	3867
		Women	3494	3494	3494
	N positive class	Overall	1292	1292	1292
		Men	702	702	702
		Women	590	590	590
2009	AUROC Mean (SD)	Overall	0.69 (2.80e-04)	0.69 (2.95e-04)	0.68 (7.84e-04)
		Men	0.69 (3.33e-04)	0.69 (3.37e-04)	0.67 (8.37e-04)
		Women	0.69 (3.73e-04)	0.7 (3.77e-04)	0.68 (9.21e-04)
	N negative class	Overall	7414	7414	7414
		Men	4052	4052	4052
		Women	3362	3362	3362
	N positive class	Overall	1136	1136	1136
		Men	658	658	658
		Women	478	478	478
2010	AUROC Mean (SD)	Overall	0.68 (2.89e-04)	0.69 (4.00e-04)	0.67 (6.24e-04)
		Men	0.69 (3.16e-04)	0.7 (3.96e-04)	0.68 (5.93e-04)
		Women	0.67 (4.06e-04)	0.67 (4.63e-04)	0.66 (7.69e-04)
	N negative class	Overall	7663	7663	7663
		Men	4094	4094	4094
		Women	3569	3569	3569

	N positive class	Overall	1100	1100	1100
		Men	641	641	641
		Women	459	459	459
2011	AUROC Mean (SD)	Overall	0.7 (2.72e-04)	0.7 (3.34e-04)	0.68 (7.28e-04)
		Men	0.7 (3.20e-04)	0.7 (3.11e-04)	0.68 (7.22e-04)
		Women	0.7 (4.33e-04)	0.7 (5.71e-04)	0.68 (9.87e-04)
	N negative class	Overall	7444	7444	7444
		Men	4075	4075	4075
		Women	3369	3369	3369
	N positive class	Overall	943	943	943
		Men	511	511	511
		Women	432	432	432
2012	AUROC Mean (SD)	Overall	0.71 (2.77e-04)	0.71 (3.73e-04)	0.69 (8.47e-04)
		Men	0.69 (3.27e-04)	0.7 (4.08e-04)	0.67 (7.84e-04)
		Women	0.74 (4.40e-04)	0.74 (4.98e-04)	0.71 (1.20e-03)
	N negative class	Overall	6730	6730	6730
		Men	3661	3661	3661
		Women	3069	3069	3069
	N positive class	Overall	715	715	715
		Men	432	432	432
		Women	283	283	283
2013	AUROC Mean (SD)	Overall	0.73 (2.80e-04)	0.73 (3.51e-04)	0.71 (9.39e-04)
		Men	0.72 (2.93e-04)	0.72 (3.73e-04)	0.69 (9.82e-04)
		Women	0.75 (4.80e-04)	0.75 (5.73e-04)	0.73 (1.27e-03)
	N negative class	Overall	7103	7103	7103
		Men	3809	3809	3809
		Women	3294	3294	3294
	N positive class	Overall	703	703	703
		Men	421	421	421
		Women	282	282	282
2014	AUROC Mean (SD)	Overall	0.74 (3.09e-04)	0.74 (4.04e-04)	0.72 (8.32e-04)
		Men	0.72 (3.04e-04)	0.72 (4.00e-04)	0.71 (8.24e-04)
		Women	0.76 (4.99e-04)	0.76 (5.56e-04)	0.73 (1.20e-03)
	N negative class	Overall	7011	7011	7011
		Men	3649	3649	3649
		Women	3362	3362	3362
	N positive class	Overall	548	548	548

		Men	307	307	307
		Women	241	241	241
2015	AUROC Mean (SD)	Overall	0.74 (4.18e-04)	0.74 (4.94e-04)	0.71 (9.78e-04)
		Men	0.75 (5.12e-04)	0.75 (6.07e-04)	0.72 (1.26e-03)
		Women	0.73 (5.09e-04)	0.73 (6.35e-04)	0.71 (9.90e-04)
	N negative class	Overall	6457	6457	6457
		Men	3426	3426	3426
		Women	3031	3031	3031
	N positive class	Overall	437	437	437
		Men	246	246	246
		Women	191	191	191
2016	AUROC Mean (SD)	Overall	0.75 (3.43e-04)	0.75 (3.96e-04)	0.72 (1.01e-03)
		Men	0.71 (3.67e-04)	0.72 (4.78e-04)	0.7 (1.14e-03)
		Women	0.79 (5.76e-04)	0.8 (5.79e-04)	0.75 (1.45e-03)
	N negative class	Overall	6561	6561	6561
		Men	3561	3561	3561
		Women	3000	3000	3000
	N positive class	Overall	383	383	383
		Men	205	205	205
		Women	178	178	178
2017	AUROC Mean (SD)	Overall	0.73 (3.51e-04)	0.73 (3.86e-04)	0.72 (1.13e-03)
		Men	0.72 (4.48e-04)	0.72 (5.38e-04)	0.69 (1.29e-03)
		Women	0.76 (4.64e-04)	0.75 (5.68e-04)	0.75 (1.42e-03)
	N negative class	Overall	6820	6820	6820
		Men	3643	3643	3643
		Women	3177	3177	3177
	N positive class	Overall	297	297	297
		Men	162	162	162
		Women	135	135	135
2018	AUROC Mean (SD)	Overall	0.75 (4.75e-04)	0.75 (6.12e-04)	0.72 (1.28e-03)
		Men	0.73 (5.85e-04)	0.74 (7.58e-04)	0.69 (1.50e-03)
		Women	0.76 (5.34e-04)	0.77 (6.56e-04)	0.75 (1.53e-03)
	N negative class	Overall	6951	6951	6951
		Men	3798	3798	3798
		Women	3153	3153	3153
	N positive class	Overall	277	277	277
		Men	156	156	156

		Women	121	121	121
2019	AUROC Mean (SD)	Overall	0.71 (4.54e-04)	0.72 (5.24e-04)	0.68 (1.48e-03)
		Men	0.71 (5.14e-04)	0.72 (6.23e-04)	0.68 (1.66e-03)
		Women	0.71 (6.98e-04)	0.72 (8.22e-04)	0.69 (1.93e-03)
	N negative class	Overall	7318	7318	7318
		Men	4010	4010	4010
		Women	3308	3308	3308
	N positive class	Overall	226	226	226
		Men	125	125	125
		Women	101	101	101
2020	AUROC Mean (SD)	Overall	0.76 (6.89e-04)	0.77 (8.07e-04)	0.73 (1.40e-03)
		Men	0.77 (6.97e-04)	0.78 (7.45e-04)	0.74 (1.53e-03)
		Women	0.75 (1.08e-03)	0.76 (1.27e-03)	0.72 (2.21e-03)
	N negative class	Overall	6464	6464	6464
		Men	3409	3409	3409
		Women	3055	3055	3055
	N positive class	Overall	152	152	152
		Men	83	83	83
		Women	69	69	69
2021	AUROC Mean (SD)	Overall	0.69 (6.64e-04)	0.68 (8.05e-04)	0.68 (1.52e-03)
		Men	0.72 (8.20e-04)	0.7 (8.76e-04)	0.7 (2.01e-03)
		Women	0.66 (1.06e-03)	0.66 (1.24e-03)	0.65 (1.78e-03)
	N negative class	Overall	9712	9712	9712
		Men	4998	4998	4998
		Women	4714	4714	4714
	N positive class	Overall	109	109	109
		Men	62	62	62
		Women	47	47	47
2022	AUROC Mean (SD)	Overall	0.71 (1.16e-03)	0.71 (1.31e-03)	0.65 (2.73e-03)
		Men	0.66 (9.25e-04)	0.66 (1.39e-03)	0.63 (3.02e-03)
		Women	0.77 (2.23e-03)	0.77 (2.35e-03)	0.67 (4.39e-03)
	N negative class	Overall	6511	6511	6511
		Men	3464	3464	3464
		Women	3047	3047	3047
	N positive class	Overall	34	34	34
		Men	19	19	19
		Women	15	15	15

Supplementary Table 7. Predictive Performance (AUROC) by Year of Diabetes Diagnosis for Replica 4 (SSMR-Refined).

Diabetes diagnosis year	Metric	Sex	Hybrid	Real	Synthetic
2003	AUROC Mean (SD)	Overall	0.62 (3.73e-04)	0.62 (3.19e-04)	0.62 (5.38e-04)
		Men	0.62 (5.80e-04)	0.62 (3.64e-04)	0.62 (7.61e-04)
		Women	0.63 (5.36e-04)	0.62 (3.90e-04)	0.62 (6.36e-04)
	N negative class	Overall	4544	4544	4544
		Men	2131	2131	2131
		Women	2413	2413	2413
	N positive class	Overall	1474	1474	1474
		Men	765	765	765
		Women	709	709	709
2004	AUROC Mean (SD)	Overall	0.63 (3.33e-04)	0.63 (3.41e-04)	0.63 (5.66e-04)
		Men	0.62 (6.21e-04)	0.62 (4.10e-04)	0.61 (8.50e-04)
		Women	0.65 (4.07e-04)	0.65 (3.78e-04)	0.65 (5.49e-04)
	N negative class	Overall	5390	5390	5390
		Men	2572	2572	2572
		Women	2818	2818	2818
	N positive class	Overall	1425	1425	1425
		Men	739	739	739
		Women	686	686	686
2005	AUROC Mean (SD)	Overall	0.65 (2.86e-04)	0.65 (2.44e-04)	0.65 (5.72e-04)
		Men	0.64 (4.92e-04)	0.64 (3.24e-04)	0.64 (8.28e-04)
		Women	0.66 (3.90e-04)	0.66 (3.61e-04)	0.66 (5.38e-04)
	N negative class	Overall	5166	5166	5166
		Men	2582	2582	2582
		Women	2584	2584	2584
	N positive class	Overall	1330	1330	1330
		Men	718	718	718
		Women	612	612	612
2006	AUROC Mean (SD)	Overall	0.66 (2.94e-04)	0.65 (3.06e-04)	0.66 (5.07e-04)
		Men	0.66 (3.67e-04)	0.66 (3.51e-04)	0.66 (6.21e-04)
		Women	0.66 (4.54e-04)	0.66 (3.94e-04)	0.66 (6.13e-04)

	N negative class	Overall	5682	5682	5682
		Men	2859	2859	2859
		Women	2823	2823	2823
	N positive class	Overall	1252	1252	1252
		Men	705	705	705
		Women	547	547	547
2007	AUROC Mean (SD)	Overall	0.67 (3.03e-04)	0.67 (2.70e-04)	0.67 (5.38e-04)
		Men	0.68 (3.17e-04)	0.68 (2.89e-04)	0.68 (5.78e-04)
		Women	0.67 (4.66e-04)	0.66 (4.06e-04)	0.67 (6.38e-04)
	N negative class	Overall	7016	7016	7016
		Men	3612	3612	3612
		Women	3404	3404	3404
	N positive class	Overall	1460	1460	1460
		Men	791	791	791
		Women	669	669	669
2008	AUROC Mean (SD)	Overall	0.67 (3.28e-04)	0.67 (3.29e-04)	0.67 (5.75e-04)
		Men	0.65 (4.17e-04)	0.65 (3.88e-04)	0.65 (7.25e-04)
		Women	0.68 (4.77e-04)	0.68 (4.59e-04)	0.69 (7.71e-04)
	N negative class	Overall	7361	7361	7361
		Men	3867	3867	3867
		Women	3494	3494	3494
	N positive class	Overall	1292	1292	1292
		Men	702	702	702
		Women	590	590	590
2009	AUROC Mean (SD)	Overall	0.69 (2.77e-04)	0.69 (2.95e-04)	0.69 (5.34e-04)
		Men	0.69 (3.19e-04)	0.69 (3.37e-04)	0.69 (6.04e-04)
		Women	0.69 (4.11e-04)	0.7 (3.77e-04)	0.69 (7.38e-04)
	N negative class	Overall	7414	7414	7414
		Men	4052	4052	4052
		Women	3362	3362	3362
	N positive class	Overall	1136	1136	1136
		Men	658	658	658
		Women	478	478	478
2010	AUROC Mean (SD)	Overall	0.68 (3.77e-04)	0.69 (4.00e-04)	0.68 (7.15e-04)
		Men	0.7 (4.12e-04)	0.7 (3.96e-04)	0.69 (8.55e-04)
		Women	0.67 (5.36e-04)	0.67 (4.63e-04)	0.67 (1.01e-03)
	N negative class	Overall	7663	7663	7663

		Men	4094	4094	4094
		Women	3569	3569	3569
	N positive class	Overall	1100	1100	1100
		Men	641	641	641
		Women	459	459	459
2011	AUROC Mean (SD)	Overall	0.7 (3.68e-04)	0.7 (3.34e-04)	0.7 (5.73e-04)
		Men	0.7 (3.38e-04)	0.7 (3.11e-04)	0.69 (7.12e-04)
		Women	0.7 (6.53e-04)	0.7 (5.71e-04)	0.7 (8.47e-04)
	N negative class	Overall	7444	7444	7444
		Men	4075	4075	4075
		Women	3369	3369	3369
	N positive class	Overall	943	943	943
		Men	511	511	511
		Women	432	432	432
2012	AUROC Mean (SD)	Overall	0.71 (3.43e-04)	0.71 (3.73e-04)	0.71 (6.87e-04)
		Men	0.7 (4.60e-04)	0.7 (4.08e-04)	0.69 (7.69e-04)
		Women	0.73 (5.64e-04)	0.74 (4.98e-04)	0.73 (9.72e-04)
	N negative class	Overall	6730	6730	6730
		Men	3661	3661	3661
		Women	3069	3069	3069
	N positive class	Overall	715	715	715
		Men	432	432	432
		Women	283	283	283
2013	AUROC Mean (SD)	Overall	0.73 (3.02e-04)	0.73 (3.51e-04)	0.72 (9.04e-04)
		Men	0.72 (3.61e-04)	0.72 (3.73e-04)	0.72 (9.46e-04)
		Women	0.75 (5.61e-04)	0.75 (5.73e-04)	0.73 (1.29e-03)
	N negative class	Overall	7103	7103	7103
		Men	3809	3809	3809
		Women	3294	3294	3294
	N positive class	Overall	703	703	703
		Men	421	421	421
		Women	282	282	282
2014	AUROC Mean (SD)	Overall	0.74 (3.73e-04)	0.74 (4.04e-04)	0.74 (8.42e-04)
		Men	0.72 (4.30e-04)	0.72 (4.00e-04)	0.72 (9.02e-04)
		Women	0.76 (5.54e-04)	0.76 (5.56e-04)	0.75 (1.19e-03)
	N negative class	Overall	7011	7011	7011
		Men	3649	3649	3649

		Women	3362	3362	3362
	N positive class	Overall	548	548	548
		Men	307	307	307
		Women	241	241	241
2015	AUROC Mean (SD)	Overall	0.74 (3.96e-04)	0.74 (4.94e-04)	0.72 (1.19e-03)
		Men	0.75 (4.81e-04)	0.75 (6.07e-04)	0.72 (1.71e-03)
		Women	0.73 (6.86e-04)	0.73 (6.35e-04)	0.72 (1.16e-03)
	N negative class	Overall	6457	6457	6457
		Men	3426	3426	3426
		Women	3031	3031	3031
	N positive class	Overall	437	437	437
		Men	246	246	246
		Women	191	191	191
2016	AUROC Mean (SD)	Overall	0.75 (4.48e-04)	0.75 (3.96e-04)	0.73 (1.18e-03)
		Men	0.72 (5.69e-04)	0.72 (4.78e-04)	0.7 (1.58e-03)
		Women	0.79 (5.77e-04)	0.8 (5.79e-04)	0.77 (1.35e-03)
	N negative class	Overall	6561	6561	6561
		Men	3561	3561	3561
		Women	3000	3000	3000
	N positive class	Overall	383	383	383
		Men	205	205	205
		Women	178	178	178
2017	AUROC Mean (SD)	Overall	0.73 (4.34e-04)	0.73 (3.86e-04)	0.71 (1.09e-03)
		Men	0.71 (5.58e-04)	0.72 (5.38e-04)	0.7 (1.34e-03)
		Women	0.75 (5.65e-04)	0.75 (5.68e-04)	0.72 (1.66e-03)
	N negative class	Overall	6820	6820	6820
		Men	3643	3643	3643
		Women	3177	3177	3177
	N positive class	Overall	297	297	297
		Men	162	162	162
		Women	135	135	135
2018	AUROC Mean (SD)	Overall	0.75 (5.78e-04)	0.75 (6.12e-04)	0.73 (1.42e-03)
		Men	0.74 (7.52e-04)	0.74 (7.58e-04)	0.73 (1.64e-03)
		Women	0.76 (6.44e-04)	0.77 (6.56e-04)	0.74 (1.83e-03)
	N negative class	Overall	6951	6951	6951
		Men	3798	3798	3798
		Women	3153	3153	3153

	N positive class	Overall	277	277	277
		Men	156	156	156
		Women	121	121	121
2019	AUROC Mean (SD)	Overall	0.71 (6.31e-04)	0.72 (5.24e-04)	0.7 (1.80e-03)
		Men	0.72 (6.69e-04)	0.72 (6.23e-04)	0.71 (1.96e-03)
		Women	0.71 (1.03e-03)	0.72 (8.22e-04)	0.68 (2.58e-03)
	N negative class	Overall	7318	7318	7318
		Men	4010	4010	4010
		Women	3308	3308	3308
	N positive class	Overall	226	226	226
		Men	125	125	125
		Women	101	101	101
2020	AUROC Mean (SD)	Overall	0.77 (6.34e-04)	0.77 (8.07e-04)	0.75 (1.56e-03)
		Men	0.77 (6.78e-04)	0.78 (7.45e-04)	0.74 (2.13e-03)
		Women	0.77 (1.21e-03)	0.76 (1.27e-03)	0.75 (2.40e-03)
	N negative class	Overall	6464	6464	6464
		Men	3409	3409	3409
		Women	3055	3055	3055
	N positive class	Overall	152	152	152
		Men	83	83	83
		Women	69	69	69
2021	AUROC Mean (SD)	Overall	0.68 (6.06e-04)	0.68 (8.05e-04)	0.68 (1.65e-03)
		Men	0.7 (7.58e-04)	0.7 (8.76e-04)	0.68 (1.92e-03)
		Women	0.66 (9.85e-04)	0.66 (1.24e-03)	0.68 (2.30e-03)
	N negative class	Overall	9712	9712	9712
		Men	4998	4998	4998
		Women	4714	4714	4714
	N positive class	Overall	109	109	109
		Men	62	62	62
		Women	47	47	47
2022	AUROC Mean (SD)	Overall	0.71 (1.35e-03)	0.71 (1.31e-03)	0.65 (3.68e-03)
		Men	0.69 (1.46e-03)	0.66 (1.39e-03)	0.67 (3.96e-03)
		Women	0.74 (2.43e-03)	0.77 (2.35e-03)	0.63 (6.76e-03)
	N negative class	Overall	6511	6511	6511
		Men	3464	3464	3464
		Women	3047	3047	3047
	N positive class	Overall	34	34	34

		Men	19	19	19
		Women	15	15	15

Multiple Splits - Single Replica

Loss curves for Synthetic Generation

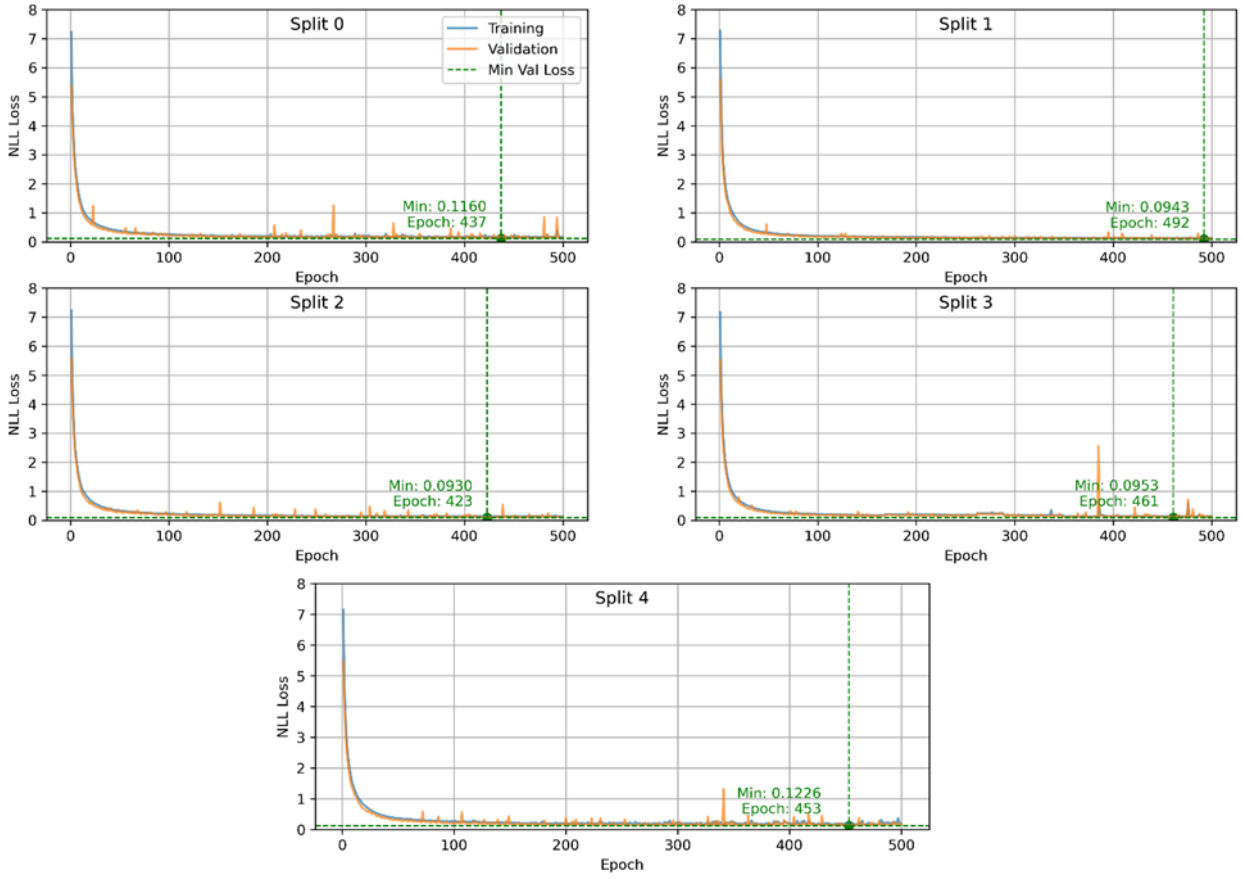

Supplementary Figure 7: Negative log-likelihood (NLL) Loss Curves (MSSR). Training and validation curves were obtained for data generation with a fixed-epoch approach (500 epochs). Although NLL Loss values stabilized around 100-200 epochs, minimal NLL values are found after 400 epochs in all the five generated cohorts.

Raw cohort Descriptive Analysis

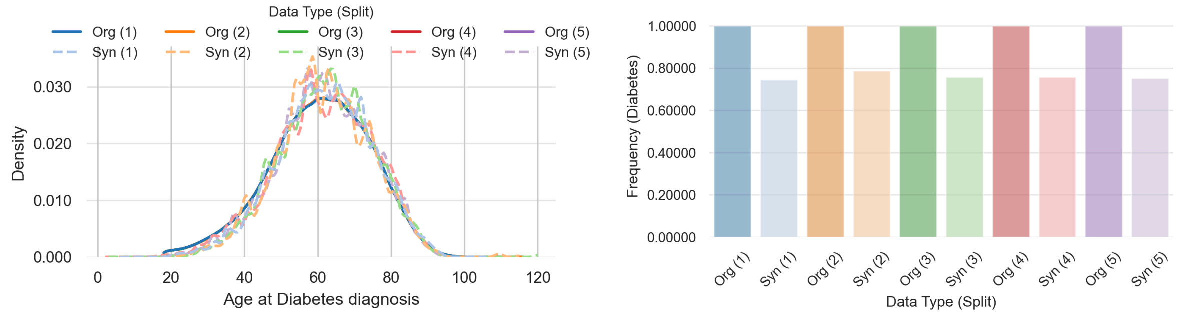

Supplementary Figure 8: Comparison of Diabetes Diagnosis Age and Frequency (MSSR-Raw). The figure illustrates the stability of the generative process across five different train-test splits of the original data. **(Left)** The density plot compares the age at diabetes diagnosis distributions between the five real data splits (solid lines) and their corresponding paired synthetic replicas (dashed lines), showing consistent alignment. **(Right)** The bar chart shows the frequency of patients with a diabetes diagnosis for each real split (100%) and each synthetic replica, confirming that the raw generative model does not consistently produce a diabetes diagnosis for every synthetic individual, regardless of the initial training data split.

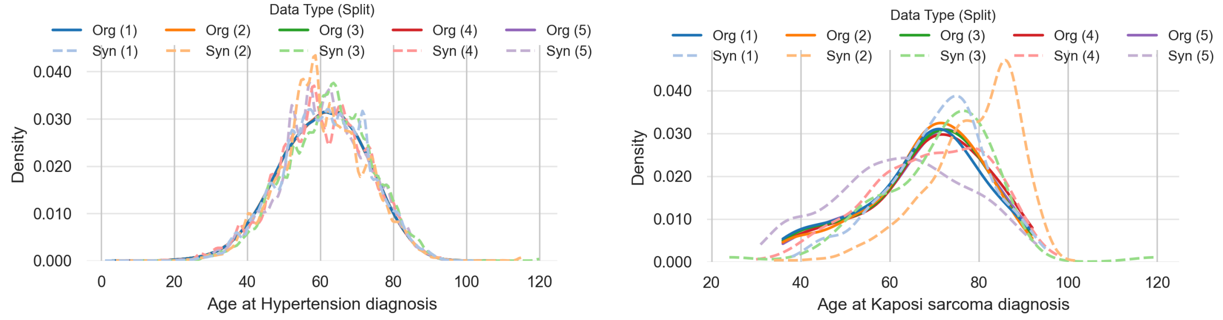

Supplementary Figure 9: Comparison of Age at Diagnosis Distributions (MSSR-Raw). The density plots compare the age at diagnosis distributions for a high-prevalence comorbidity (Hypertension, left) and a low-prevalence one (Kaposi's sarcoma, right). The distributions for the five real data splits (solid lines) are shown alongside their corresponding paired synthetic replicas (dashed lines). The plots demonstrate the generative model's consistent ability to capture the distribution of the high-prevalence disease, while showing greater variability when modeling the rare disease across different training data splits.

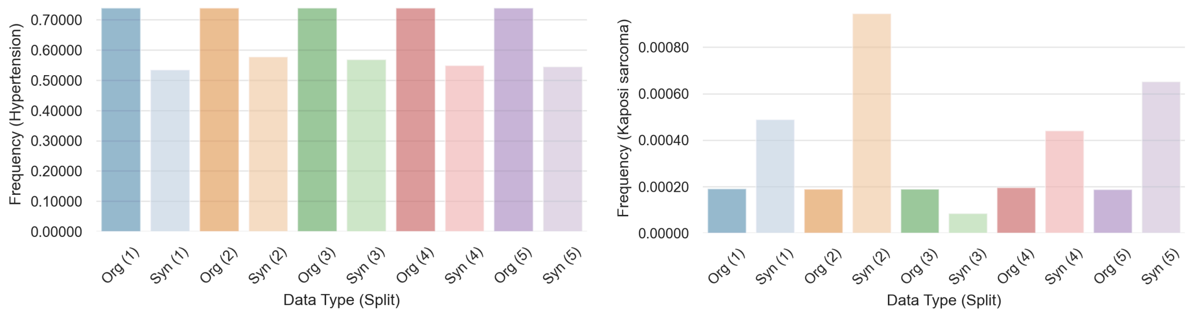

Supplementary Figure 10: Comparison of Categorical Disease Frequencies (MSSR-Raw). The bar charts compare the frequency of a high-prevalence comorbidity (Hypertension, left) and a low-prevalence one (Kaposi's sarcoma, right) between the five different real data splits (darker bars) and their corresponding paired synthetic replicas (lighter bars). The plots show that while the frequency of the common disease is relatively stable, the prevalence of the rare disease shows significant variability across both the real data splits and the synthetic replicas.

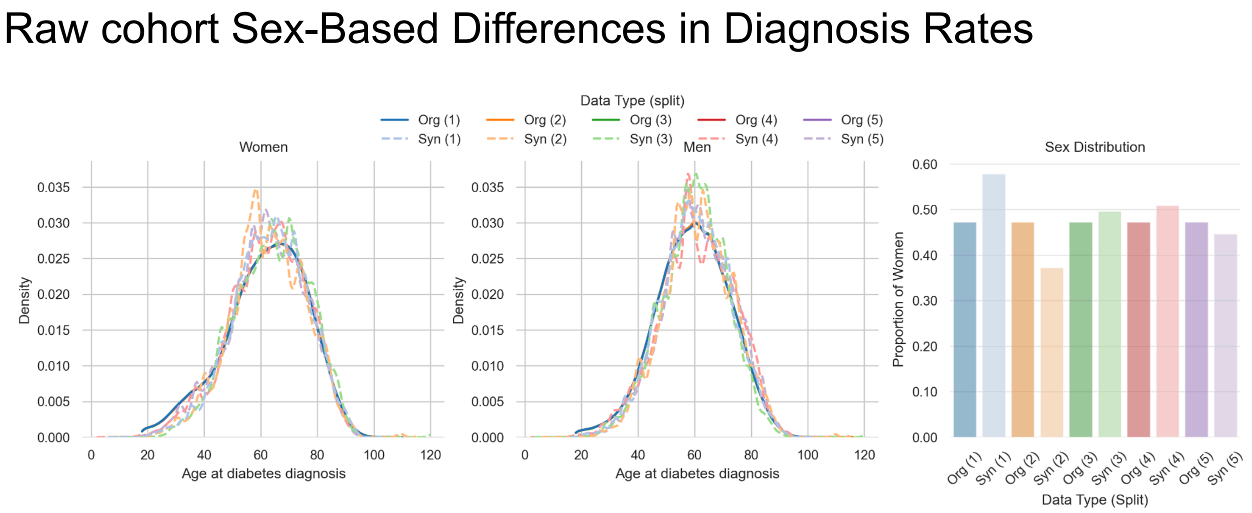

Supplementary Figure 11: Comparison of Age at Diabetes Diagnosis and Sex Distribution (MSSR-Raw). The figure illustrates the stability of the generative process across five different train-test splits of the original data. (Left & Center) The density plots compare the age at diabetes diagnosis distributions for women and men between the five real data splits (solid lines) and their corresponding paired synthetic replicas (dashed lines), showing consistent alignment. (Right) The bar chart shows the proportion of women for each real split and each synthetic replica, demonstrating a slight but consistent overrepresentation of women in the synthetic data, regardless of the initial training data split.

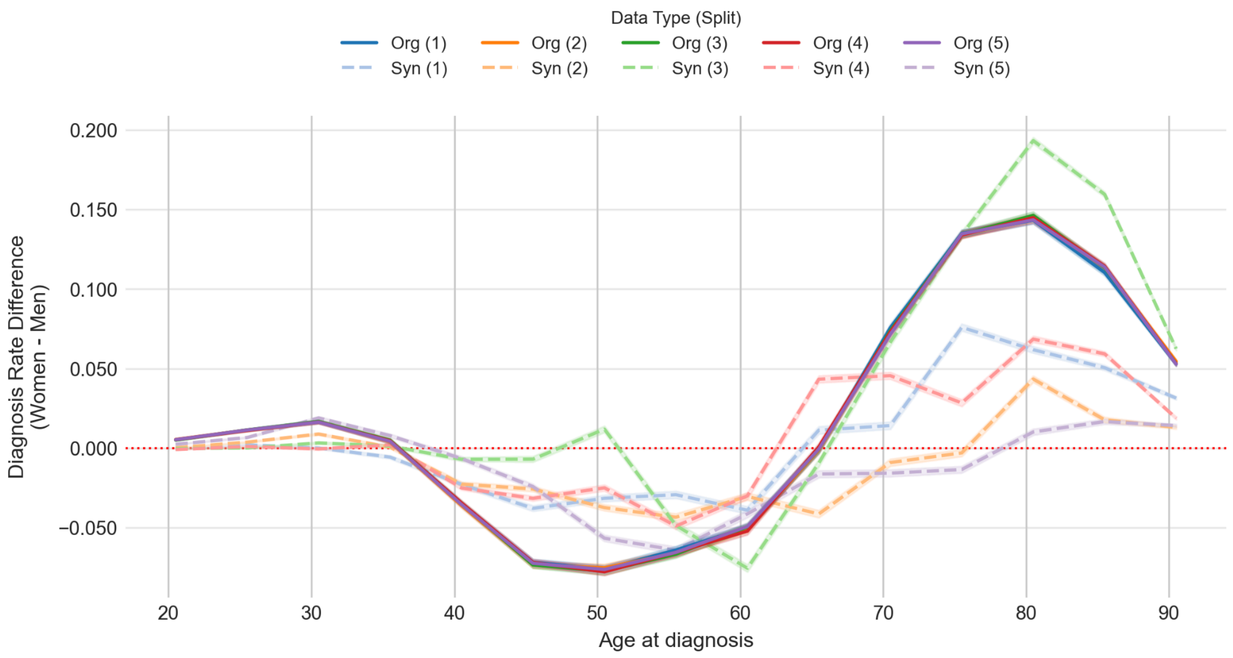

Supplementary Figure 12. Sex-Based Differences in the Rate of Diagnosis Across the Lifespan (Raw). The plot displays the absolute difference in the per-person diagnosis rate (Women - Men) across 5-year age intervals for the five different real data splits (solid lines) and their corresponding paired synthetic replicas (dashed lines). The results show that the general biomedical pattern is present across the different real data splits, and that the variability in how well the synthetic replicas capture this pattern is a consistent feature of the generative model, independent of the specific training data.

Type	Split_i	Split_j	DTW_Distance
Org-Org	1	2	0,01650298619
Org-Org	1	3	0,01601977268
Org-Org	1	4	0,01875182694
Org-Org	1	5	0,01261810776
Org-Org	2	3	0,01251181534
Org-Org	2	4	0,01526272518
Org-Org	2	5	0,008597199379
Org-Org	3	4	0,01227729936
Org-Org	3	5	0,01158663353
Org-Org	4	5	0,01247262788
Org-Syn	1	1	0,3928858674
Org-Syn	2	2	0,5190604374
Org-Syn	3	3	0,2232032456
Org-Syn	4	4	0,4805823572
Org-Syn	5	5	0,5583299566

Supplementary Table 8. DTW distances between different Original and paired Replicas (MSSR-Raw). The 'Org-Org' rows show the DTW distances between different random splits of the original data, demonstrating the inherent stability of the biomedical pattern (low distance). The 'Org-Syn' rows show the DTW distance between each original data split and its corresponding synthetic replica. The significantly higher distances in this group confirm that the variability in replicating the biomedical trajectory is a characteristic of the generative model and not an artifact of the initial data split.

Refined Cohort Descriptive Analysis

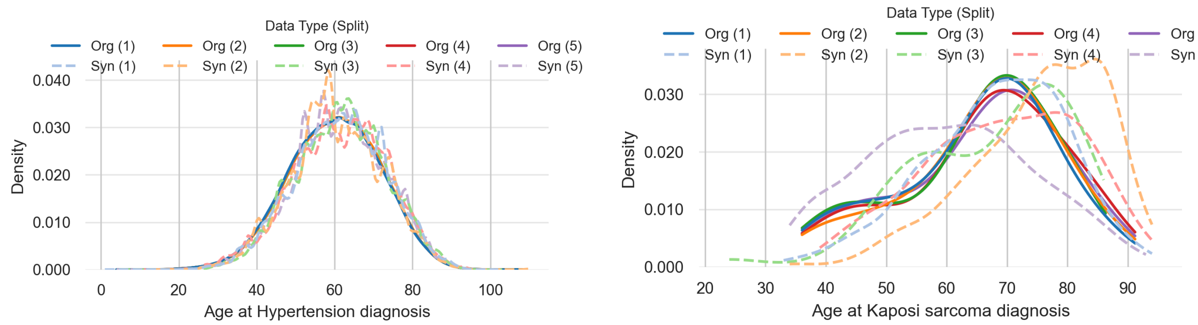

Supplementary Figure 13: Comparison of Age at Diagnosis Distributions (MSSR-Refined). The density plots compare the age at diagnosis distributions for a high-prevalence comorbidity (Hypertension, left) and a low-prevalence one (Kaposi's sarcoma, right). The distributions for the five real data splits (solid lines) are shown alongside their corresponding paired synthetic replicas (dashed lines). The plots demonstrate the generative model's consistent ability to capture the distribution of the high-prevalence disease, while showing greater variability when modeling the rare disease. A comparison with the raw cohort (Supplementary Figure 6) reveals that these distributional patterns are largely preserved after the refinement process.

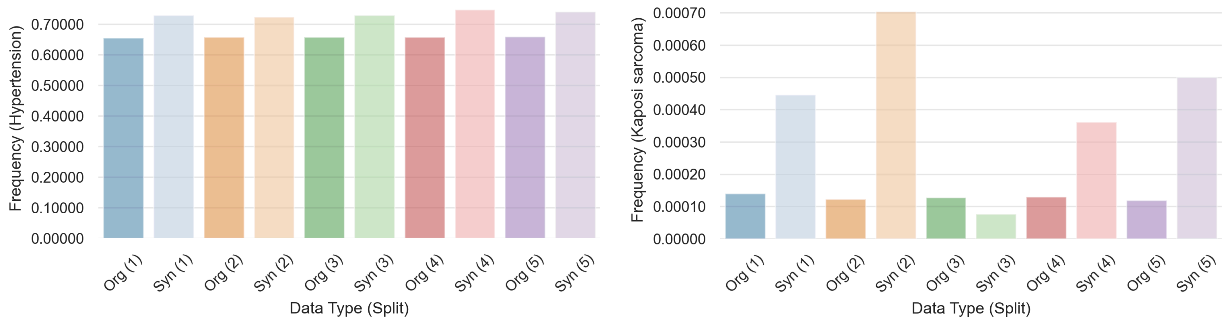

Supplementary Figure 14: Comparison of Categorical Disease Frequencies (MSSR-Refined). The bar charts compare the frequency of a high-prevalence comorbidity (Hypertension, left) and a low-prevalence one (Kaposi's sarcoma, right) between the five different real data splits (darker bars) and their corresponding paired synthetic replicas (lighter bars). The plots show that while the frequency of the common disease is relatively stable, the prevalence of the rare disease shows significant variability. A

comparison with the raw cohort analysis (Supplementary Figure 7) confirms that these frequency patterns are consistent before and after the refinement process.

Refined Cohort Sex-Based Differences in Diagnosis Rates

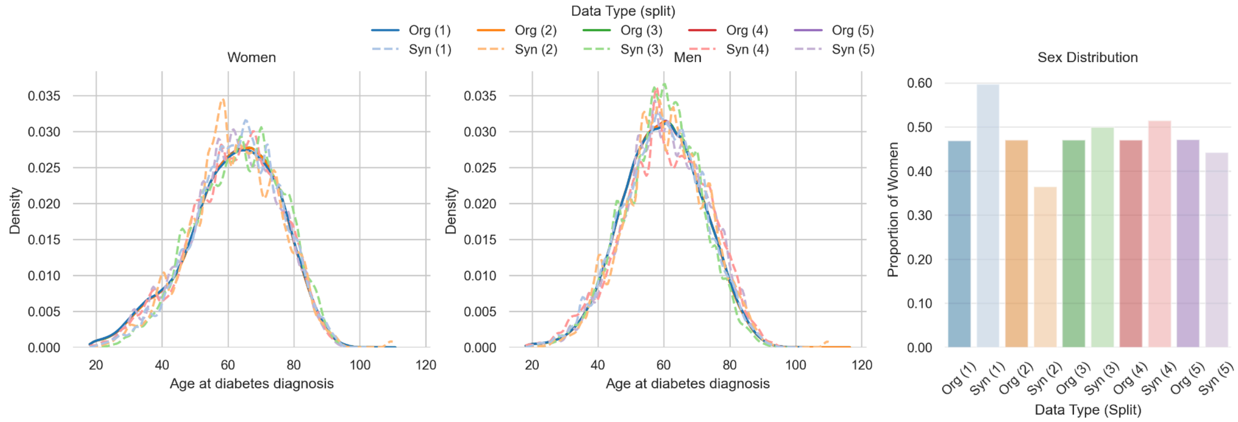

Supplementary Figure 15: Comparison of Age at Diabetes Diagnosis and Sex Distribution (MSSR-Refined). The figure illustrates the stability of the generative process across five different train-test splits of the original data. (Left & Center) The density plots compare the age at diabetes diagnosis distributions for women and men between the five real data splits (solid lines) and their corresponding paired synthetic replicas (dashed lines). (Right) The bar chart shows the proportion of women for each real split and each synthetic replica. A comparison with the raw cohort analysis (Supplementary Figure 8) confirms that the distributional shapes and the slight overrepresentation of women in the synthetic data are consistent patterns that are preserved after the refinement process.

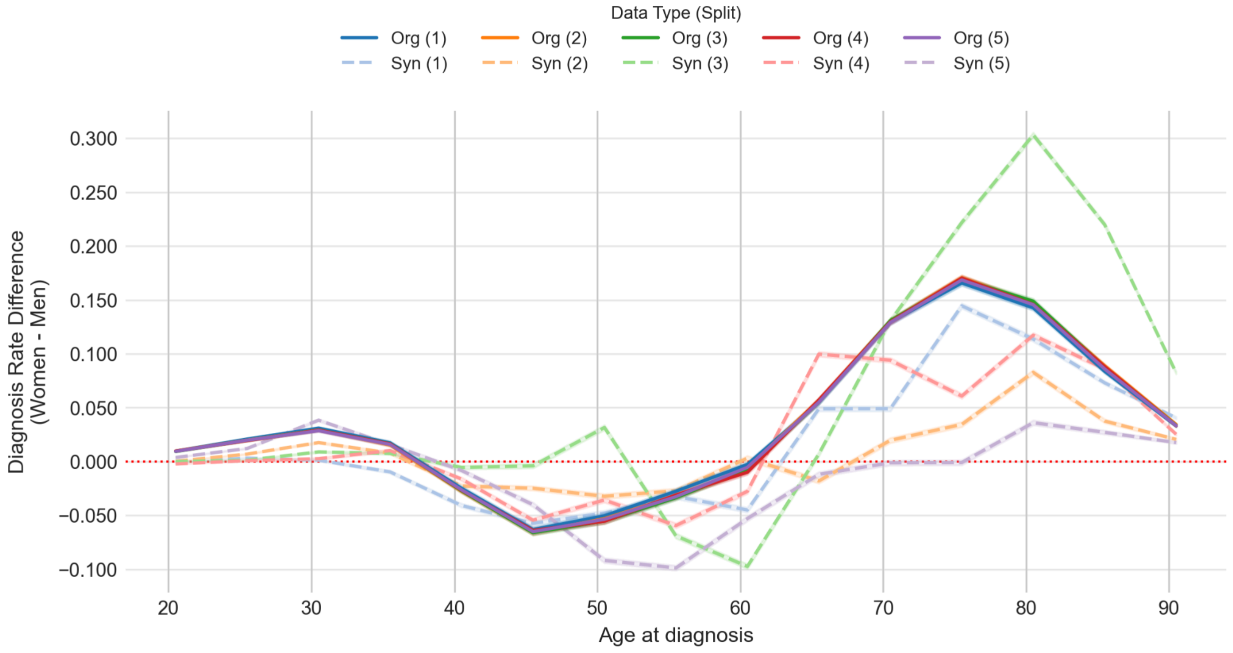

Supplementary Figure 16. Sex-Based Differences in the Rate of Diagnosis Across the Lifespan (MSSR-Refined). The plot displays the absolute difference and its 95% CI in the per-person diagnosis rate (Women - Men) across 5-year age intervals for the five different real data splits (solid lines) and their corresponding paired synthetic replicas (dashed lines).

Type	Split_1	Split_2	DTW_Distance
Org-Org	1	2	0,03967951491
Org-Org	1	3	0,04030669177
Org-Org	1	4	0,03730739835
Org-Org	1	5	0,02828427202
Org-Org	2	3	0,01298395406
Org-Org	2	4	0,01617682182
Org-Org	2	5	0,01518965332
Org-Org	3	4	0,01665794891
Org-Org	3	5	0,01511398228
Org-Org	4	5	0,01789206394
Org-Syn	1	1	0,2507168231
Org-Syn	2	2	0,3931504094
Org-Syn	3	3	0,5718870364
Org-Syn	4	4	0,3143154053
Org-Syn	5	5	0,5714694464

Supplementary Table 9. DTW distances between different Original and paired Replicas (MSSR-Refined). The 'Org-Org' rows show the DTW distances between different random splits of the original data, demonstrating the inherent stability of the biomedical pattern (low distance). The 'Org-Syn' rows show the DTW distance between each original data split and its corresponding synthetic replica. The significantly higher distances in this group confirm that the variability in replicating the biomedical trajectory is a characteristic of the generative model and not an artifact of the initial data split. Therefore, the DTW distances between the real and synthetic curves are of a similar magnitude to those observed in the raw cohort (Supplementary Table 8), indicating that the variability in replicating this biomedical pattern is a consistent characteristic of the generative model that is not significantly altered by the pre-processing pipeline.

Refined Cohort Comorbidities Prevalence

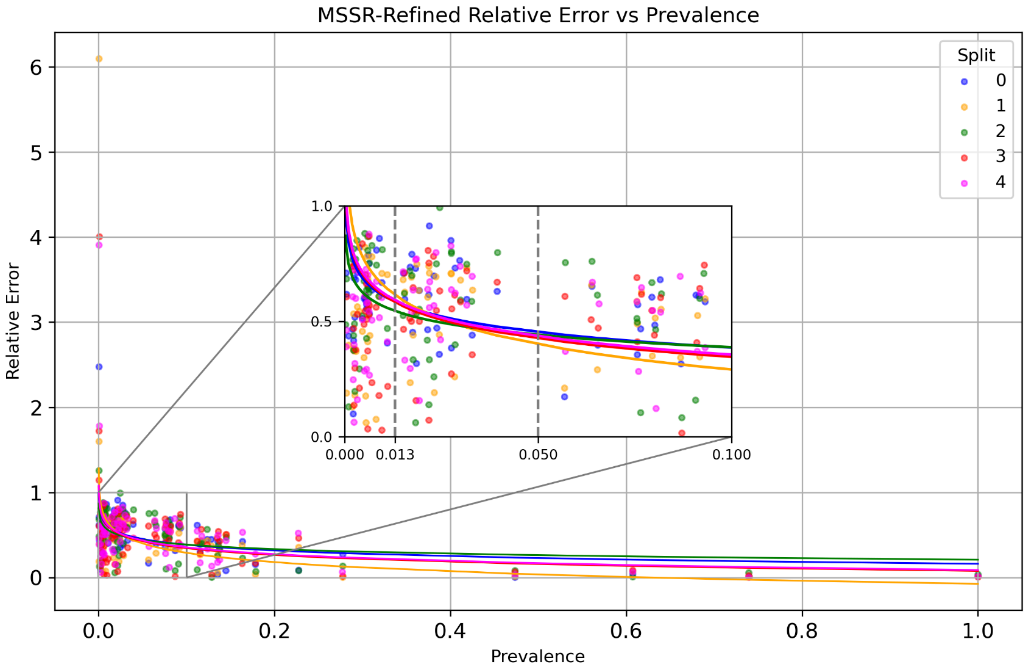

Supplementary Figure 17: Relative error against original prevalence for individual comorbidities (MSSR-Refined). Consistent thresholds as in SSMR-Refined cohorts were identified for prevalence of 0.05 and 0.013, below which synthetic generation becomes increasingly sparse and unreliable.

Train-Synthetic, Test-Real performance

Predictive performance

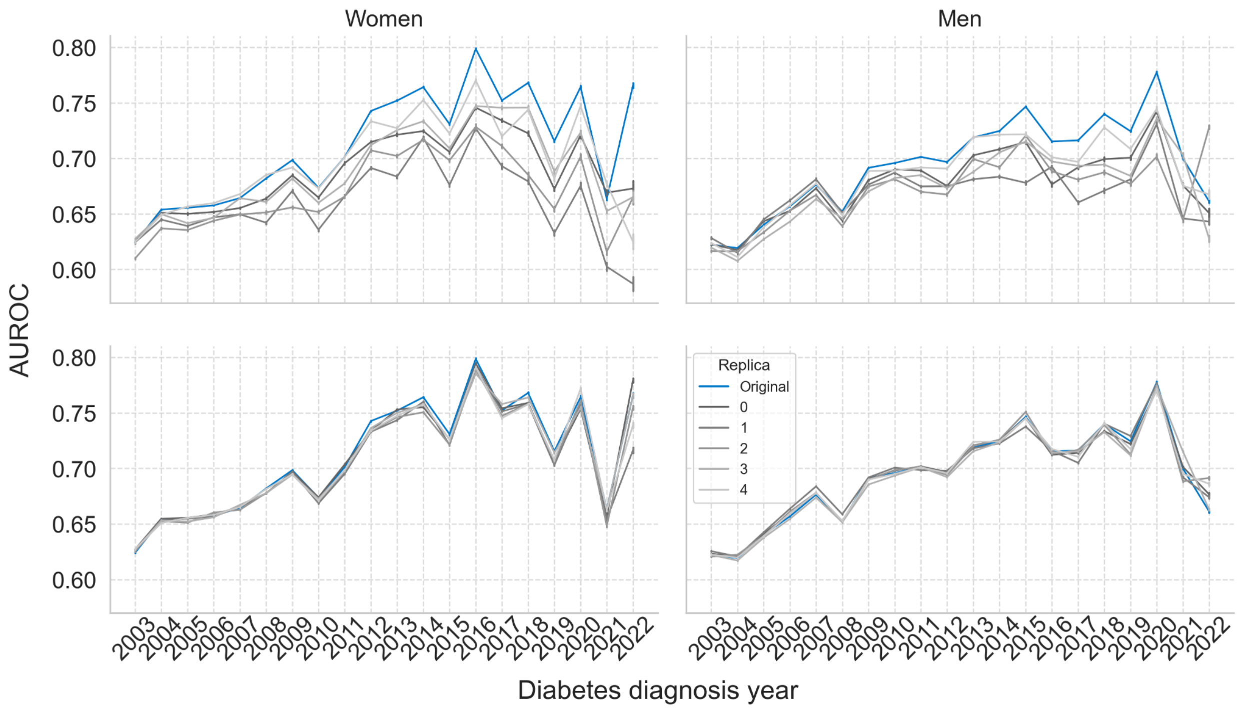

Supplementary Figure 18. Predictive Performance (AUROC) by Year of Diabetes Diagnosis (SSMR-Refined).

Comparison of model performance for women **(upper-left panel)** and men **(upper-right panel)** trained on original data (blue) or on independent synthetic replicas under a single dataset split (grey shades). Models trained on the original data demonstrate improving performance over time, with slightly greater temporal stability in men than in women. Models trained on synthetic replicas closely track the performance of those trained on the original data, albeit with a small performance gap, and similarly exhibit greater stability in men than in women. The **lower-left** and **lower-right** panels show the performance of models trained on augmented datasets combining original and independent synthetic replicas for women and men, respectively. These hybrid models largely overlap with those trained solely on original data in both sexes, indicating that augmentation with synthetic data does not improve predictive performance.

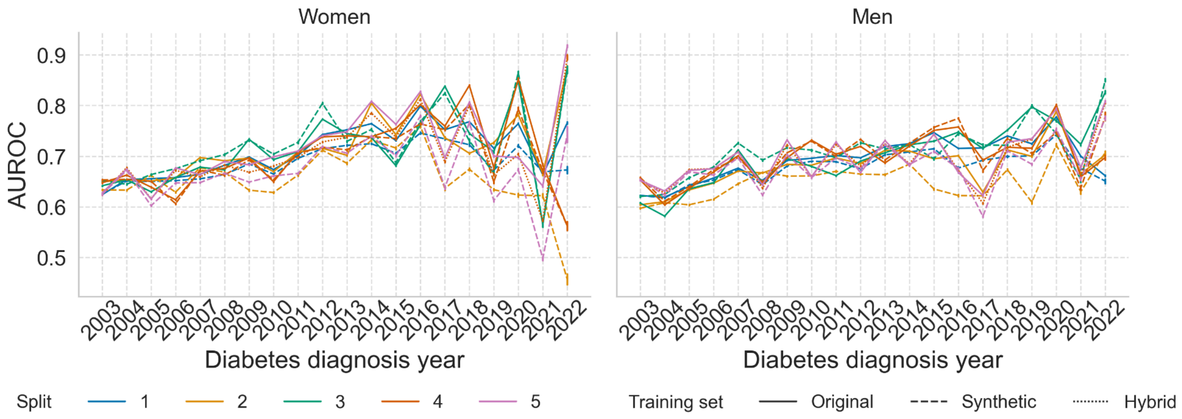

Supplementary Figure 19. Predictive Performance (AUROC) by Year of Diabetes Diagnosis (MSSR-Refined).

Comparison of model performance for women **(left panel)** and men **(right panel)**, evaluating models trained on original, synthetic, and hybrid data across multiple dataset splits. Across splits, the models exhibit similar performance trends in both sexes, indicating that predictive performance for CKD onset is robust and not driven by the initial patient split.

Supplementary Table 10. Predictive Performance (AUROC) by Year of Diabetes for Split 1 (MSSR-Refined).

Diabetes diagnosis year	Metric	Sex	Hybrid	Real	Synthetic
2003	AUROC Mean (SD)	Overall	0.62 (2.44e-04)	0.62 (3.19e-04)	0.62 (7.08e-04)
		Men	0.62 (3.20e-04)	0.62 (3.64e-04)	0.62 (6.47e-04)
		Women	0.63 (3.04e-04)	0.62 (3.90e-04)	0.63 (5.70e-04)
	N negative class	Overall	4544	4544	4544
		Men	2131	2131	2131
		Women	2413	2413	2413
	N positive class	Overall	1474	1474	1474
		Men	765	765	765
		Women	709	709	709
2004	AUROC Mean (SD)	Overall	0.64 (2.31e-04)	0.63 (3.41e-04)	0.63 (5.60e-04)
		Men	0.62 (3.78e-04)	0.62 (4.10e-04)	0.62 (7.26e-04)
		Women	0.65 (2.49e-04)	0.65 (3.78e-04)	0.65 (5.36e-04)
	N negative class	Overall	5390	5390	5390
		Men	2572	2572	2572
		Women	2818	2818	2818
	N positive class	Overall	1425	1425	1425

		Men	739	739	739
		Women	686	686	686
2005	AUROC Mean (SD)	Overall	0.65 (2.20e-04)	0.65 (2.44e-04)	0.64 (7.19e-04)
		Men	0.64 (3.02e-04)	0.64 (3.24e-04)	0.64 (6.89e-04)
		Women	0.66 (2.81e-04)	0.66 (3.61e-04)	0.65 (8.43e-04)
	N negative class	Overall	5166	5166	5166
		Men	2582	2582	2582
		Women	2584	2584	2584
	N positive class	Overall	1330	1330	1330
		Men	718	718	718
		Women	612	612	612
2006	AUROC Mean (SD)	Overall	0.66 (2.60e-04)	0.65 (3.06e-04)	0.65 (7.80e-04)
		Men	0.66 (3.06e-04)	0.66 (3.51e-04)	0.65 (8.35e-04)
		Women	0.66 (3.12e-04)	0.66 (3.94e-04)	0.65 (7.93e-04)
	N negative class	Overall	5682	5682	5682
		Men	2859	2859	2859
		Women	2823	2823	2823
	N positive class	Overall	1252	1252	1252
		Men	705	705	705
		Women	547	547	547
2007	AUROC Mean (SD)	Overall	0.67 (2.68e-04)	0.67 (2.70e-04)	0.66 (7.50e-04)
		Men	0.68 (2.64e-04)	0.68 (2.89e-04)	0.67 (7.04e-04)
		Women	0.67 (3.35e-04)	0.66 (4.06e-04)	0.66 (8.12e-04)
	N negative class	Overall	7016	7016	7016
		Men	3612	3612	3612
		Women	3404	3404	3404
	N positive class	Overall	1460	1460	1460
		Men	791	791	791
		Women	669	669	669
2008	AUROC Mean (SD)	Overall	0.66 (3.10e-04)	0.67 (3.29e-04)	0.65 (9.60e-04)
		Men	0.65 (2.86e-04)	0.65 (3.88e-04)	0.64 (8.50e-04)
		Women	0.68 (4.80e-04)	0.68 (4.59e-04)	0.66 (1.09e-03)
	N negative class	Overall	7361	7361	7361
		Men	3867	3867	3867
		Women	3494	3494	3494
	N positive class	Overall	1292	1292	1292
		Men	702	702	702

		Women	590	590	590
2009	AUROC Mean (SD)	Overall	0.69 (2.83e-04)	0.69 (2.95e-04)	0.68 (8.17e-04)
		Men	0.69 (3.11e-04)	0.69 (3.37e-04)	0.68 (7.16e-04)
		Women	0.7 (3.84e-04)	0.7 (3.77e-04)	0.68 (9.64e-04)
	N negative class	Overall	7414	7414	7414
		Men	4052	4052	4052
		Women	3362	3362	3362
	N positive class	Overall	1136	1136	1136
		Men	658	658	658
		Women	478	478	478
2010	AUROC Mean (SD)	Overall	0.69 (2.98e-04)	0.69 (4.00e-04)	0.68 (8.66e-04)
		Men	0.7 (3.04e-04)	0.7 (3.96e-04)	0.69 (6.86e-04)
		Women	0.67 (4.04e-04)	0.67 (4.63e-04)	0.66 (9.82e-04)
	N negative class	Overall	7663	7663	7663
		Men	4094	4094	4094
		Women	3569	3569	3569
	N positive class	Overall	1100	1100	1100
		Men	641	641	641
		Women	459	459	459
2011	AUROC Mean (SD)	Overall	0.7 (3.07e-04)	0.7 (3.34e-04)	0.68 (9.78e-04)
		Men	0.7 (3.03e-04)	0.7 (3.11e-04)	0.69 (8.53e-04)
		Women	0.7 (5.14e-04)	0.7 (5.71e-04)	0.7 (1.16e-03)
	N negative class	Overall	7444	7444	7444
		Men	4075	4075	4075
		Women	3369	3369	3369
	N positive class	Overall	943	943	943
		Men	511	511	511
		Women	432	432	432
2012	AUROC Mean (SD)	Overall	0.71 (3.25e-04)	0.71 (3.73e-04)	0.69 (9.47e-04)
		Men	0.7 (3.64e-04)	0.7 (4.08e-04)	0.68 (1.11e-03)
		Women	0.73 (4.45e-04)	0.74 (4.98e-04)	0.71 (1.12e-03)
	N negative class	Overall	6730	6730	6730
		Men	3661	3661	3661
		Women	3069	3069	3069
	N positive class	Overall	715	715	715
		Men	432	432	432
		Women	283	283	283

2013	AUROC Mean (SD)	Overall	0.73 (3.12e-04)	0.73 (3.51e-04)	0.71 (9.02e-04)
		Men	0.72 (3.38e-04)	0.72 (3.73e-04)	0.7 (1.01e-03)
		Women	0.75 (5.44e-04)	0.75 (5.73e-04)	0.72 (1.37e-03)
	N negative class	Overall	7103	7103	7103
		Men	3809	3809	3809
		Women	3294	3294	3294
	N positive class	Overall	703	703	703
		Men	421	421	421
		Women	282	282	282
2014	AUROC Mean (SD)	Overall	0.74 (3.35e-04)	0.74 (4.04e-04)	0.71 (9.66e-04)
		Men	0.73 (3.17e-04)	0.72 (4.00e-04)	0.71 (1.03e-03)
		Women	0.76 (5.11e-04)	0.76 (5.56e-04)	0.72 (1.18e-03)
	N negative class	Overall	7011	7011	7011
		Men	3649	3649	3649
		Women	3362	3362	3362
	N positive class	Overall	548	548	548
		Men	307	307	307
		Women	241	241	241
2015	AUROC Mean (SD)	Overall	0.74 (3.91e-04)	0.74 (4.94e-04)	0.71 (1.17e-03)
		Men	0.75 (4.57e-04)	0.75 (6.07e-04)	0.71 (1.40e-03)
		Women	0.73 (5.85e-04)	0.73 (6.35e-04)	0.71 (1.33e-03)
	N negative class	Overall	6457	6457	6457
		Men	3426	3426	3426
		Women	3031	3031	3031
	N positive class	Overall	437	437	437
		Men	246	246	246
		Women	191	191	191
2016	AUROC Mean (SD)	Overall	0.75 (4.38e-04)	0.75 (3.96e-04)	0.7 (1.54e-03)
		Men	0.71 (5.36e-04)	0.72 (4.78e-04)	0.68 (2.01e-03)
		Women	0.79 (5.27e-04)	0.8 (5.79e-04)	0.75 (1.65e-03)
	N negative class	Overall	6561	6561	6561
		Men	3561	3561	3561
		Women	3000	3000	3000
	N positive class	Overall	383	383	383
		Men	205	205	205
		Women	178	178	178
2017	AUROC Mean (SD)	Overall	0.73 (3.75e-04)	0.73 (3.86e-04)	0.7 (1.12e-03)

		Men	0.71 (5.01e-04)	0.72 (5.38e-04)	0.69 (1.37e-03)
		Women	0.75 (5.58e-04)	0.75 (5.68e-04)	0.73 (1.47e-03)
	N negative class	Overall	6820	6820	6820
		Men	3643	3643	3643
		Women	3177	3177	3177
	N positive class	Overall	297	297	297
		Men	162	162	162
		Women	135	135	135
2018	AUROC Mean (SD)	Overall	0.74 (5.59e-04)	0.75 (6.12e-04)	0.7 (1.63e-03)
		Men	0.73 (6.86e-04)	0.74 (7.58e-04)	0.7 (1.71e-03)
		Women	0.76 (6.08e-04)	0.77 (6.56e-04)	0.72 (2.13e-03)
	N negative class	Overall	6951	6951	6951
		Men	3798	3798	3798
		Women	3153	3153	3153
	N positive class	Overall	277	277	277
		Men	156	156	156
		Women	121	121	121
2019	AUROC Mean (SD)	Overall	0.71 (4.94e-04)	0.72 (5.24e-04)	0.68 (1.53e-03)
		Men	0.72 (5.75e-04)	0.72 (6.23e-04)	0.7 (2.10e-03)
		Women	0.71 (8.25e-04)	0.72 (8.22e-04)	0.67 (2.01e-03)
	N negative class	Overall	7318	7318	7318
		Men	4010	4010	4010
		Women	3308	3308	3308
	N positive class	Overall	226	226	226
		Men	125	125	125
		Women	101	101	101
2020	AUROC Mean (SD)	Overall	0.76 (6.72e-04)	0.77 (8.07e-04)	0.72 (1.56e-03)
		Men	0.77 (7.13e-04)	0.78 (7.45e-04)	0.74 (1.84e-03)
		Women	0.75 (1.16e-03)	0.76 (1.27e-03)	0.72 (2.43e-03)
	N negative class	Overall	6464	6464	6464
		Men	3409	3409	3409
		Women	3055	3055	3055
	N positive class	Overall	152	152	152
		Men	83	83	83
		Women	69	69	69
2021	AUROC Mean (SD)	Overall	0.68 (6.16e-04)	0.68 (8.05e-04)	0.67 (1.69e-03)
		Men	0.7 (6.23e-04)	0.7 (8.76e-04)	0.68 (1.90e-03)

		Women	0.66 (1.02e-03)	0.66 (1.24e-03)	0.67 (2.44e-03)
	N negative class	Overall	9712	9712	9712
		Men	4998	4998	4998
		Women	4714	4714	4714
	N positive class	Overall	109	109	109
		Men	62	62	62
		Women	47	47	47
2022	AUROC Mean (SD)	Overall	0.72 (1.11e-03)	0.71 (1.31e-03)	0.66 (3.69e-03)
		Men	0.68 (1.39e-03)	0.66 (1.39e-03)	0.65 (4.13e-03)
		Women	0.78 (1.92e-03)	0.77 (2.35e-03)	0.67 (6.91e-03)
	N negative class	Overall	6511	6511	6511
		Men	3464	3464	3464
		Women	3047	3047	3047
	N positive class	Overall	34	34	34
		Men	19	19	19
		Women	15	15	15

Supplementary Table 11. Predictive Performance (AUROC) by Year of Diabetes Diagnosis for Split 2 (MSSR-Refined).

Diabetes diagnosis year	Metric	Sex	Hybrid	Real	Synthetic
2003	AUROC Mean (SD)	Overall	0.62 (6.62e-04)	0.62 (3.74e-04)	0.62 (7.82e-04)
		Men	0.6 (5.67e-04)	0.6 (6.47e-04)	0.6 (1.17e-03)
		Women	0.65 (5.97e-04)	0.65 (6.46e-04)	0.63 (1.21e-03)
	N negative class	Overall	4544	4544	4544
		Men	2131	2131	2131
		Women	2413	2413	2413
	N positive class	Overall	1474	1474	1474
		Men	765	765	765
		Women	709	709	709
2004	AUROC Mean (SD)	Overall	0.62 (4.65e-04)	0.63 (5.06e-04)	0.62 (6.77e-04)
		Men	0.61 (7.56e-04)	0.61 (7.68e-04)	0.61 (1.15e-03)
		Women	0.65 (5.80e-04)	0.65 (6.54e-04)	0.63 (1.23e-03)
	N negative class	Overall	5390	5390	5390
		Men	2572	2572	2572

		Women	2818	2818	2818
	N positive class	Overall	1425	1425	1425
		Men	739	739	739
		Women	686	686	686
2005	AUROC Mean (SD)	Overall	0.63 (5.71e-04)	0.64 (4.82e-04)	0.64 (8.57e-04)
		Men	0.63 (6.89e-04)	0.63 (7.58e-04)	0.6 (1.20e-03)
		Women	0.66 (6.59e-04)	0.65 (5.81e-04)	0.66 (1.12e-03)
	N negative class	Overall	5166	5166	5166
		Men	2582	2582	2582
		Women	2584	2584	2584
	N positive class	Overall	1330	1330	1330
		Men	718	718	718
		Women	612	612	612
2006	AUROC Mean (SD)	Overall	0.65 (4.38e-04)	0.65 (4.26e-04)	0.62 (8.55e-04)
		Men	0.64 (6.22e-04)	0.65 (7.00e-04)	0.62 (1.01e-03)
		Women	0.66 (6.97e-04)	0.66 (7.59e-04)	0.63 (1.13e-03)
	N negative class	Overall	5682	5682	5682
		Men	2859	2859	2859
		Women	2823	2823	2823
	N positive class	Overall	1252	1252	1252
		Men	705	705	705
		Women	547	547	547
2007	AUROC Mean (SD)	Overall	0.67 (6.17e-04)	0.68 (6.31e-04)	0.66 (1.04e-03)
		Men	0.67 (6.28e-04)	0.67 (5.58e-04)	0.65 (1.45e-03)
		Women	0.69 (9.18e-04)	0.7 (1.10e-03)	0.68 (1.62e-03)
	N negative class	Overall	7016	7016	7016
		Men	3612	3612	3612
		Women	3404	3404	3404
	N positive class	Overall	1460	1460	1460
		Men	791	791	791
		Women	669	669	669
2008	AUROC Mean (SD)	Overall	0.68 (4.71e-04)	0.68 (4.92e-04)	0.67 (9.25e-04)
		Men	0.67 (6.87e-04)	0.67 (5.77e-04)	0.67 (1.10e-03)
		Women	0.7 (8.37e-04)	0.69 (9.05e-04)	0.67 (1.44e-03)
	N negative class	Overall	7361	7361	7361
		Men	3867	3867	3867
		Women	3494	3494	3494

	N positive class	Overall	1292	1292	1292
		Men	702	702	702
		Women	590	590	590
2009	AUROC Mean (SD)	Overall	0.68 (5.62e-04)	0.69 (4.44e-04)	0.65 (8.98e-04)
		Men	0.68 (6.77e-04)	0.68 (5.97e-04)	0.66 (1.14e-03)
		Women	0.69 (9.33e-04)	0.7 (8.11e-04)	0.63 (1.66e-03)
	N negative class	Overall	7414	7414	7414
		Men	4052	4052	4052
		Women	3362	3362	3362
	N positive class	Overall	1136	1136	1136
		Men	658	658	658
		Women	478	478	478
2010	AUROC Mean (SD)	Overall	0.67 (5.09e-04)	0.68 (5.01e-04)	0.65 (1.05e-03)
		Men	0.68 (4.80e-04)	0.68 (5.07e-04)	0.66 (1.09e-03)
		Women	0.67 (7.24e-04)	0.67 (8.31e-04)	0.63 (1.42e-03)
	N negative class	Overall	7663	7663	7663
		Men	4094	4094	4094
		Women	3569	3569	3569
	N positive class	Overall	1100	1100	1100
		Men	641	641	641
		Women	459	459	459
2011	AUROC Mean (SD)	Overall	0.7 (4.91e-04)	0.7 (5.34e-04)	0.67 (1.09e-03)
		Men	0.69 (6.38e-04)	0.7 (7.17e-04)	0.67 (1.46e-03)
		Women	0.7 (7.20e-04)	0.71 (7.56e-04)	0.66 (1.57e-03)
	N negative class	Overall	7444	7444	7444
		Men	4075	4075	4075
		Women	3369	3369	3369
	N positive class	Overall	943	943	943
		Men	511	511	511
		Women	432	432	432
2012	AUROC Mean (SD)	Overall	0.7 (5.78e-04)	0.7 (5.28e-04)	0.69 (1.01e-03)
		Men	0.69 (8.40e-04)	0.69 (7.02e-04)	0.66 (1.37e-03)
		Women	0.72 (9.27e-04)	0.72 (9.24e-04)	0.71 (1.53e-03)
	N negative class	Overall	6730	6730	6730
		Men	3661	3661	3661
		Women	3069	3069	3069
	N positive class	Overall	715	715	715

		Men	432	432	432
		Women	283	283	283
2013	AUROC Mean (SD)	Overall	0.71 (6.01e-04)	0.71 (6.78e-04)	0.68 (1.31e-03)
		Men	0.71 (6.26e-04)	0.71 (6.05e-04)	0.66 (1.31e-03)
		Women	0.71 (1.09e-03)	0.7 (1.50e-03)	0.69 (2.11e-03)
	N negative class	Overall	7103	7103	7103
		Men	3809	3809	3809
		Women	3294	3294	3294
	N positive class	Overall	703	703	703
		Men	421	421	421
		Women	282	282	282
2014	AUROC Mean (SD)	Overall	0.75 (6.60e-04)	0.76 (7.22e-04)	0.71 (1.72e-03)
		Men	0.71 (6.45e-04)	0.71 (1.00e-03)	0.68 (1.80e-03)
		Women	0.79 (1.03e-03)	0.8 (1.12e-03)	0.73 (2.53e-03)
	N negative class	Overall	7011	7011	7011
		Men	3649	3649	3649
		Women	3362	3362	3362
	N positive class	Overall	548	548	548
		Men	307	307	307
		Women	241	241	241
2015	AUROC Mean (SD)	Overall	0.71 (6.37e-04)	0.71 (7.53e-04)	0.67 (1.41e-03)
		Men	0.69 (7.46e-04)	0.69 (1.01e-03)	0.64 (1.66e-03)
		Women	0.74 (1.15e-03)	0.74 (1.22e-03)	0.72 (2.30e-03)
	N negative class	Overall	6457	6457	6457
		Men	3426	3426	3426
		Women	3031	3031	3031
	N positive class	Overall	437	437	437
		Men	246	246	246
		Women	191	191	191
2016	AUROC Mean (SD)	Overall	0.75 (6.12e-04)	0.76 (7.04e-04)	0.69 (1.78e-03)
		Men	0.69 (8.48e-04)	0.7 (8.84e-04)	0.62 (2.38e-03)
		Women	0.82 (8.33e-04)	0.82 (1.15e-03)	0.76 (2.56e-03)
	N negative class	Overall	6561	6561	6561
		Men	3561	3561	3561
		Women	3000	3000	3000
	N positive class	Overall	383	383	383
		Men	205	205	205

		Women	178	178	178
2017	AUROC Mean (SD)	Overall	0.67 (8.23e-04)	0.67 (1.01e-03)	0.63 (1.95e-03)
		Men	0.63 (9.65e-04)	0.63 (1.23e-03)	0.62 (2.14e-03)
		Women	0.72 (1.92e-03)	0.74 (2.22e-03)	0.64 (3.91e-03)
	N negative class	Overall	6820	6820	6820
		Men	3643	3643	3643
		Women	3177	3177	3177
	N positive class	Overall	297	297	297
		Men	162	162	162
		Women	135	135	135
2018	AUROC Mean (SD)	Overall	0.7 (9.60e-04)	0.71 (1.11e-03)	0.67 (1.43e-03)
		Men	0.7 (1.42e-03)	0.71 (1.66e-03)	0.67 (1.85e-03)
		Women	0.71 (1.29e-03)	0.71 (1.46e-03)	0.67 (2.45e-03)
	N negative class	Overall	6951	6951	6951
		Men	3798	3798	3798
		Women	3153	3153	3153
	N positive class	Overall	277	277	277
		Men	156	156	156
		Women	121	121	121
2019	AUROC Mean (SD)	Overall	0.7 (8.77e-04)	0.71 (8.57e-04)	0.62 (2.21e-03)
		Men	0.69 (1.18e-03)	0.7 (9.74e-04)	0.61 (3.24e-03)
		Women	0.71 (1.26e-03)	0.73 (1.75e-03)	0.63 (2.10e-03)
	N negative class	Overall	7318	7318	7318
		Men	4010	4010	4010
		Women	3308	3308	3308
	N positive class	Overall	226	226	226
		Men	125	125	125
		Women	101	101	101
2020	AUROC Mean (SD)	Overall	0.76 (9.71e-04)	0.79 (9.50e-04)	0.69 (2.60e-03)
		Men	0.77 (1.18e-03)	0.79 (1.06e-03)	0.72 (3.36e-03)
		Women	0.74 (1.74e-03)	0.78 (2.50e-03)	0.62 (3.74e-03)
	N negative class	Overall	6464	6464	6464
		Men	3409	3409	3409
		Women	3055	3055	3055
	N positive class	Overall	152	152	152
		Men	83	83	83
		Women	69	69	69

2021	AUROC Mean (SD)	Overall	0.67 (1.32e-03)	0.67 (2.13e-03)	0.64 (3.40e-03)
		Men	0.68 (1.79e-03)	0.66 (2.97e-03)	0.63 (4.78e-03)
		Women	0.66 (2.22e-03)	0.67 (3.47e-03)	0.62 (4.15e-03)
	N negative class	Overall	9712	9712	9712
		Men	4998	4998	4998
		Women	4714	4714	4714
	N positive class	Overall	109	109	109
		Men	62	62	62
		Women	47	47	47
2022	AUROC Mean (SD)	Overall	0.74 (2.16e-03)	0.77 (1.92e-03)	0.62 (3.01e-03)
		Men	0.7 (2.00e-03)	0.7 (2.10e-03)	0.71 (2.65e-03)
		Women	0.8 (5.06e-03)	0.87 (3.54e-03)	0.46 (1.06e-02)
	N negative class	Overall	6511	6511	6511
		Men	3464	3464	3464
		Women	3047	3047	3047
	N positive class	Overall	34	34	34
		Men	19	19	19
		Women	15	15	15

Supplementary Table 11. Predictive Performance (AUROC) by Year of Diabetes Diagnosis for Split 3 (MSSR-Refined).

Diabetes diagnosis year	Metric	Sex	Hybrid	Real	Synthetic
2003	AUROC Mean (SD)	Overall	0.62 (6.63e-04)	0.62 (8.01e-04)	0.62 (1.62e-03)
		Men	0.61 (9.86e-04)	0.61 (7.63e-04)	0.62 (1.92e-03)
		Women	0.64 (7.35e-04)	0.64 (7.70e-04)	0.65 (1.70e-03)
	N negative class	Overall	4544	4544	4544
		Men	2131	2131	2131
		Women	2413	2413	2413
	N positive class	Overall	1474	1474	1474
		Men	765	765	765
		Women	709	709	709
2004	AUROC Mean (SD)	Overall	0.62 (6.72e-04)	0.61 (6.59e-04)	0.62 (1.43e-03)
		Men	0.6 (1.00e-03)	0.58 (9.10e-04)	0.62 (2.48e-03)
		Women	0.65 (9.39e-04)	0.65 (7.81e-04)	0.64 (1.49e-03)

	N negative class	Overall	5390	5390	5390
		Men	2572	2572	2572
		Women	2818	2818	2818
	N positive class	Overall	1425	1425	1425
		Men	739	739	739
		Women	686	686	686
2005	AUROC Mean (SD)	Overall	0.63 (6.89e-04)	0.63 (5.56e-04)	0.64 (1.01e-03)
		Men	0.64 (1.09e-03)	0.64 (7.70e-04)	0.66 (1.70e-03)
		Women	0.63 (8.88e-04)	0.63 (8.80e-04)	0.66 (1.45e-03)
	N negative class	Overall	5166	5166	5166
		Men	2582	2582	2582
		Women	2584	2584	2584
	N positive class	Overall	1330	1330	1330
		Men	718	718	718
		Women	612	612	612
2006	AUROC Mean (SD)	Overall	0.66 (7.53e-04)	0.65 (6.40e-04)	0.67 (1.33e-03)
		Men	0.65 (9.70e-04)	0.65 (7.43e-04)	0.68 (1.52e-03)
		Women	0.66 (8.24e-04)	0.66 (8.34e-04)	0.68 (2.16e-03)
	N negative class	Overall	5682	5682	5682
		Men	2859	2859	2859
		Women	2823	2823	2823
	N positive class	Overall	1252	1252	1252
		Men	705	705	705
		Women	547	547	547
2007	AUROC Mean (SD)	Overall	0.71 (5.07e-04)	0.69 (6.58e-04)	0.7 (1.27e-03)
		Men	0.72 (6.25e-04)	0.71 (6.21e-04)	0.73 (1.41e-03)
		Women	0.69 (7.97e-04)	0.68 (9.45e-04)	0.69 (2.13e-03)
	N negative class	Overall	7016	7016	7016
		Men	3612	3612	3612
		Women	3404	3404	3404
	N positive class	Overall	1460	1460	1460
		Men	791	791	791
		Women	669	669	669
2008	AUROC Mean (SD)	Overall	0.67 (6.27e-04)	0.66 (5.87e-04)	0.69 (1.09e-03)
		Men	0.66 (8.13e-04)	0.65 (7.16e-04)	0.69 (1.60e-03)
		Women	0.67 (8.21e-04)	0.67 (9.85e-04)	0.7 (1.63e-03)
	N negative class	Overall	7361	7361	7361

		Men	3867	3867	3867
		Women	3494	3494	3494
	N positive class	Overall	1292	1292	1292
		Men	702	702	702
		Women	590	590	590
2009	AUROC Mean (SD)	Overall	0.72 (5.42e-04)	0.71 (4.41e-04)	0.72 (1.24e-03)
		Men	0.7 (7.70e-04)	0.7 (5.92e-04)	0.72 (1.02e-03)
		Women	0.73 (7.90e-04)	0.73 (7.46e-04)	0.73 (2.01e-03)
	N negative class	Overall	7414	7414	7414
		Men	4052	4052	4052
		Women	3362	3362	3362
	N positive class	Overall	1136	1136	1136
		Men	658	658	658
		Women	478	478	478
2010	AUROC Mean (SD)	Overall	0.7 (5.07e-04)	0.69 (7.29e-04)	0.7 (1.25e-03)
		Men	0.69 (7.74e-04)	0.68 (7.89e-04)	0.71 (1.70e-03)
		Women	0.7 (7.30e-04)	0.69 (1.23e-03)	0.7 (1.65e-03)
	N negative class	Overall	7663	7663	7663
		Men	4094	4094	4094
		Women	3569	3569	3569
	N positive class	Overall	1100	1100	1100
		Men	641	641	641
		Women	459	459	459
2011	AUROC Mean (SD)	Overall	0.7 (6.67e-04)	0.68 (6.64e-04)	0.7 (1.26e-03)
		Men	0.68 (8.43e-04)	0.66 (7.08e-04)	0.7 (1.52e-03)
		Women	0.72 (8.91e-04)	0.71 (1.14e-03)	0.73 (1.90e-03)
	N negative class	Overall	7444	7444	7444
		Men	4075	4075	4075
		Women	3369	3369	3369
	N positive class	Overall	943	943	943
		Men	511	511	511
		Women	432	432	432
2012	AUROC Mean (SD)	Overall	0.73 (7.62e-04)	0.72 (6.46e-04)	0.75 (1.37e-03)
		Men	0.7 (8.54e-04)	0.69 (7.96e-04)	0.73 (1.77e-03)
		Women	0.78 (1.12e-03)	0.77 (9.96e-04)	0.8 (1.89e-03)
	N negative class	Overall	6730	6730	6730
		Men	3661	3661	3661

		Women	3069	3069	3069
	N positive class	Overall	715	715	715
		Men	432	432	432
		Women	283	283	283
2013	AUROC Mean (SD)	Overall	0.73 (5.79e-04)	0.72 (7.59e-04)	0.71 (1.44e-03)
		Men	0.72 (8.63e-04)	0.71 (8.82e-04)	0.71 (1.58e-03)
		Women	0.74 (8.00e-04)	0.74 (1.06e-03)	0.73 (1.96e-03)
	N negative class	Overall	7103	7103	7103
		Men	3809	3809	3809
		Women	3294	3294	3294
	N positive class	Overall	703	703	703
		Men	421	421	421
		Women	282	282	282
2014	AUROC Mean (SD)	Overall	0.73 (5.82e-04)	0.73 (7.11e-04)	0.74 (1.50e-03)
		Men	0.73 (6.31e-04)	0.72 (8.47e-04)	0.72 (1.93e-03)
		Women	0.74 (1.24e-03)	0.74 (1.17e-03)	0.75 (2.24e-03)
	N negative class	Overall	7011	7011	7011
		Men	3649	3649	3649
		Women	3362	3362	3362
	N positive class	Overall	548	548	548
		Men	307	307	307
		Women	241	241	241
2015	AUROC Mean (SD)	Overall	0.71 (7.30e-04)	0.71 (7.39e-04)	0.69 (1.90e-03)
		Men	0.73 (9.74e-04)	0.73 (1.01e-03)	0.7 (2.63e-03)
		Women	0.68 (9.39e-04)	0.68 (1.02e-03)	0.69 (1.74e-03)
	N negative class	Overall	6457	6457	6457
		Men	3426	3426	3426
		Women	3031	3031	3031
	N positive class	Overall	437	437	437
		Men	246	246	246
		Women	191	191	191
2016	AUROC Mean (SD)	Overall	0.76 (7.46e-04)	0.75 (9.40e-04)	0.76 (1.72e-03)
		Men	0.76 (1.13e-03)	0.75 (1.26e-03)	0.74 (3.11e-03)
		Women	0.78 (1.27e-03)	0.76 (1.34e-03)	0.77 (1.83e-03)
	N negative class	Overall	6561	6561	6561
		Men	3561	3561	3561
		Women	3000	3000	3000

	N positive class	Overall	383	383	383
		Men	205	205	205
		Women	178	178	178
2017	AUROC Mean (SD)	Overall	0.77 (7.09e-04)	0.77 (7.74e-04)	0.76 (1.44e-03)
		Men	0.72 (1.04e-03)	0.72 (1.27e-03)	0.72 (2.41e-03)
		Women	0.83 (1.05e-03)	0.84 (9.27e-04)	0.82 (1.84e-03)
	N negative class	Overall	6820	6820	6820
		Men	3643	3643	3643
		Women	3177	3177	3177
	N positive class	Overall	297	297	297
		Men	162	162	162
		Women	135	135	135
2018	AUROC Mean (SD)	Overall	0.75 (1.01e-03)	0.75 (8.02e-04)	0.72 (1.95e-03)
		Men	0.75 (1.17e-03)	0.75 (1.05e-03)	0.72 (2.22e-03)
		Women	0.74 (1.58e-03)	0.74 (1.43e-03)	0.73 (3.57e-03)
	N negative class	Overall	6951	6951	6951
		Men	3798	3798	3798
		Women	3153	3153	3153
	N positive class	Overall	277	277	277
		Men	156	156	156
		Women	121	121	121
2019	AUROC Mean (SD)	Overall	0.77 (1.00e-03)	0.77 (1.14e-03)	0.75 (1.96e-03)
		Men	0.8 (1.36e-03)	0.8 (1.47e-03)	0.8 (2.60e-03)
		Women	0.71 (1.34e-03)	0.71 (2.10e-03)	0.67 (3.99e-03)
	N negative class	Overall	7318	7318	7318
		Men	4010	4010	4010
		Women	3308	3308	3308
	N positive class	Overall	226	226	226
		Men	125	125	125
		Women	101	101	101
2020	AUROC Mean (SD)	Overall	0.81 (1.00e-03)	0.8 (1.58e-03)	0.78 (2.67e-03)
		Men	0.78 (1.28e-03)	0.77 (1.95e-03)	0.74 (2.97e-03)
		Women	0.86 (1.32e-03)	0.85 (1.56e-03)	0.87 (2.57e-03)
	N negative class	Overall	6464	6464	6464
		Men	3409	3409	3409
		Women	3055	3055	3055
	N positive class	Overall	152	152	152

		Men	83	83	83
		Women	69	69	69
2021	AUROC Mean (SD)	Overall	0.65 (1.32e-03)	0.64 (1.95e-03)	0.61 (3.56e-03)
		Men	0.73 (1.66e-03)	0.72 (2.66e-03)	0.65 (4.88e-03)
		Women	0.57 (2.35e-03)	0.57 (2.89e-03)	0.56 (4.42e-03)
	N negative class	Overall	9712	9712	9712
		Men	4998	4998	4998
		Women	4714	4714	4714
	N positive class	Overall	109	109	109
		Men	62	62	62
		Women	47	47	47
2022	AUROC Mean (SD)	Overall	0.86 (1.61e-03)	0.85 (2.43e-03)	0.86 (3.20e-03)
		Men	0.83 (1.95e-03)	0.83 (2.58e-03)	0.85 (2.63e-03)
		Women	0.88 (2.73e-03)	0.87 (4.22e-03)	0.87 (5.29e-03)
	N negative class	Overall	6511	6511	6511
		Men	3464	3464	3464
		Women	3047	3047	3047
	N positive class	Overall	34	34	34
		Men	19	19	19
		Women	15	15	15

Supplementary Table 13. Predictive Performance (AUROC) by Year of Diabetes Diagnosis for Split 4 (MSSR-Refined).

Diabetes diagnosis year	Metric	Sex	Hybrid	Real	Synthetic
2003	AUROC Mean (SD)	Overall	0.65 (4.36e-04)	0.65 (5.27e-04)	0.65 (8.12e-04)
		Men	0.65 (5.92e-04)	0.65 (6.55e-04)	0.66 (9.24e-04)
		Women	0.65 (6.97e-04)	0.65 (8.08e-04)	0.65 (1.07e-03)
	N negative class	Overall	4544	4544	4544
		Men	2131	2131	2131
		Women	2413	2413	2413
	N positive class	Overall	1474	1474	1474
		Men	765	765	765
		Women	709	709	709
2004	AUROC Mean (SD)	Overall	0.63 (4.20e-04)	0.63 (5.72e-04)	0.63 (8.72e-04)

		Men	0.6 (6.51e-04)	0.6 (7.11e-04)	0.61 (9.84e-04)
		Women	0.66 (7.80e-04)	0.66 (8.05e-04)	0.65 (1.01e-03)
	N negative class	Overall	5390	5390	5390
		Men	2572	2572	2572
		Women	2818	2818	2818
	N positive class	Overall	1425	1425	1425
		Men	739	739	739
		Women	686	686	686
2005	AUROC Mean (SD)	Overall	0.64 (5.15e-04)	0.64 (4.94e-04)	0.64 (7.08e-04)
		Men	0.64 (6.98e-04)	0.64 (7.31e-04)	0.64 (9.77e-04)
		Women	0.65 (7.50e-04)	0.64 (9.02e-04)	0.65 (1.07e-03)
	N negative class	Overall	5166	5166	5166
		Men	2582	2582	2582
		Women	2584	2584	2584
	N positive class	Overall	1330	1330	1330
		Men	718	718	718
		Women	612	612	612
2006	AUROC Mean (SD)	Overall	0.64 (4.01e-04)	0.64 (5.91e-04)	0.64 (6.45e-04)
		Men	0.67 (5.22e-04)	0.67 (6.75e-04)	0.67 (7.78e-04)
		Women	0.61 (7.90e-04)	0.61 (8.01e-04)	0.61 (1.09e-03)
	N negative class	Overall	5682	5682	5682
		Men	2859	2859	2859
		Women	2823	2823	2823
	N positive class	Overall	1252	1252	1252
		Men	705	705	705
		Women	547	547	547
2007	AUROC Mean (SD)	Overall	0.69 (3.83e-04)	0.69 (4.96e-04)	0.69 (5.89e-04)
		Men	0.7 (5.24e-04)	0.7 (7.26e-04)	0.7 (8.96e-04)
		Women	0.67 (6.78e-04)	0.67 (7.70e-04)	0.67 (1.14e-03)
	N negative class	Overall	7016	7016	7016
		Men	3612	3612	3612
		Women	3404	3404	3404
	N positive class	Overall	1460	1460	1460
		Men	791	791	791
		Women	669	669	669
2008	AUROC Mean (SD)	Overall	0.66 (4.96e-04)	0.66 (5.80e-04)	0.66 (7.73e-04)
		Men	0.65 (6.29e-04)	0.64 (5.85e-04)	0.65 (1.04e-03)

		Women	0.67 (8.92e-04)	0.68 (1.08e-03)	0.66 (1.40e-03)
	N negative class	Overall	7361	7361	7361
		Men	3867	3867	3867
		Women	3494	3494	3494
	N positive class	Overall	1292	1292	1292
		Men	702	702	702
		Women	590	590	590
2009	AUROC Mean (SD)	Overall	0.7 (5.25e-04)	0.69 (5.16e-04)	0.7 (7.03e-04)
		Men	0.7 (5.95e-04)	0.7 (6.55e-04)	0.71 (6.73e-04)
		Women	0.7 (9.33e-04)	0.7 (8.27e-04)	0.69 (1.26e-03)
	N negative class	Overall	7414	7414	7414
		Men	4052	4052	4052
		Women	3362	3362	3362
	N positive class	Overall	1136	1136	1136
		Men	658	658	658
		Women	478	478	478
2010	AUROC Mean (SD)	Overall	0.7 (5.40e-04)	0.7 (6.50e-04)	0.7 (8.62e-04)
		Men	0.73 (7.02e-04)	0.73 (7.23e-04)	0.73 (9.65e-04)
		Women	0.65 (9.01e-04)	0.65 (1.04e-03)	0.65 (1.48e-03)
	N negative class	Overall	7663	7663	7663
		Men	4094	4094	4094
		Women	3569	3569	3569
	N positive class	Overall	1100	1100	1100
		Men	641	641	641
		Women	459	459	459
2011	AUROC Mean (SD)	Overall	0.71 (4.14e-04)	0.71 (5.02e-04)	0.7 (8.99e-04)
		Men	0.71 (5.35e-04)	0.7 (6.46e-04)	0.7 (1.15e-03)
		Women	0.71 (6.88e-04)	0.71 (8.54e-04)	0.71 (1.26e-03)
	N negative class	Overall	7444	7444	7444
		Men	4075	4075	4075
		Women	3369	3369	3369
	N positive class	Overall	943	943	943
		Men	511	511	511
		Women	432	432	432
2012	AUROC Mean (SD)	Overall	0.72 (5.41e-04)	0.72 (6.06e-04)	0.72 (7.49e-04)
		Men	0.72 (7.23e-04)	0.72 (7.58e-04)	0.73 (8.80e-04)
		Women	0.73 (9.80e-04)	0.74 (9.70e-04)	0.72 (1.33e-03)

	N negative class	Overall	6730	6730	6730
		Men	3661	3661	3661
		Women	3069	3069	3069
	N positive class	Overall	715	715	715
		Men	432	432	432
		Women	283	283	283
2013	AUROC Mean (SD)	Overall	0.71 (5.97e-04)	0.71 (6.87e-04)	0.7 (1.01e-03)
		Men	0.69 (7.36e-04)	0.69 (8.44e-04)	0.69 (1.13e-03)
		Women	0.74 (9.32e-04)	0.74 (1.10e-03)	0.71 (1.67e-03)
	N negative class	Overall	7103	7103	7103
		Men	3809	3809	3809
		Women	3294	3294	3294
	N positive class	Overall	703	703	703
		Men	421	421	421
		Women	282	282	282
2014	AUROC Mean (SD)	Overall	0.73 (5.78e-04)	0.73 (6.25e-04)	0.73 (9.54e-04)
		Men	0.72 (8.45e-04)	0.72 (8.25e-04)	0.73 (1.10e-03)
		Women	0.74 (7.24e-04)	0.74 (8.02e-04)	0.74 (1.33e-03)
	N negative class	Overall	7011	7011	7011
		Men	3649	3649	3649
		Women	3362	3362	3362
	N positive class	Overall	548	548	548
		Men	307	307	307
		Women	241	241	241
2015	AUROC Mean (SD)	Overall	0.76 (6.31e-04)	0.75 (8.12e-04)	0.75 (1.21e-03)
		Men	0.76 (8.90e-04)	0.75 (9.74e-04)	0.76 (1.67e-03)
		Women	0.75 (1.04e-03)	0.75 (1.45e-03)	0.73 (2.57e-03)
	N negative class	Overall	6457	6457	6457
		Men	3426	3426	3426
		Women	3031	3031	3031
	N positive class	Overall	437	437	437
		Men	246	246	246
		Women	191	191	191
2016	AUROC Mean (SD)	Overall	0.77 (6.37e-04)	0.78 (7.57e-04)	0.76 (1.24e-03)
		Men	0.76 (7.69e-04)	0.76 (8.01e-04)	0.77 (1.10e-03)
		Women	0.79 (8.88e-04)	0.8 (9.33e-04)	0.76 (2.09e-03)
	N negative class	Overall	6561	6561	6561

		Men	3561	3561	3561
		Women	3000	3000	3000
	N positive class	Overall	383	383	383
		Men	205	205	205
		Women	178	178	178
2017	AUROC Mean (SD)	Overall	0.71 (9.75e-04)	0.73 (1.06e-03)	0.71 (1.57e-03)
		Men	0.69 (1.80e-03)	0.69 (1.77e-03)	0.67 (2.70e-03)
		Women	0.75 (1.35e-03)	0.76 (1.24e-03)	0.75 (2.07e-03)
	N negative class	Overall	6820	6820	6820
		Men	3643	3643	3643
		Women	3177	3177	3177
	N positive class	Overall	297	297	297
		Men	162	162	162
		Women	135	135	135
2018	AUROC Mean (SD)	Overall	0.77 (7.69e-04)	0.77 (7.48e-04)	0.77 (1.72e-03)
		Men	0.72 (1.21e-03)	0.72 (1.03e-03)	0.74 (1.93e-03)
		Women	0.84 (9.61e-04)	0.84 (1.09e-03)	0.8 (3.55e-03)
	N negative class	Overall	6951	6951	6951
		Men	3798	3798	3798
		Women	3153	3153	3153
	N positive class	Overall	277	277	277
		Men	156	156	156
		Women	121	121	121
2019	AUROC Mean (SD)	Overall	0.69 (1.00e-03)	0.69 (1.14e-03)	0.67 (1.92e-03)
		Men	0.72 (1.11e-03)	0.72 (1.19e-03)	0.71 (2.34e-03)
		Women	0.67 (1.57e-03)	0.68 (2.06e-03)	0.65 (2.81e-03)
	N negative class	Overall	7318	7318	7318
		Men	4010	4010	4010
		Women	3308	3308	3308
	N positive class	Overall	226	226	226
		Men	125	125	125
		Women	101	101	101
2020	AUROC Mean (SD)	Overall	0.8 (9.89e-04)	0.82 (1.31e-03)	0.77 (2.23e-03)
		Men	0.77 (1.43e-03)	0.8 (1.54e-03)	0.75 (3.01e-03)
		Women	0.84 (1.49e-03)	0.85 (2.15e-03)	0.79 (3.70e-03)
	N negative class	Overall	6464	6464	6464
		Men	3409	3409	3409

		Women	3055	3055	3055
	N positive class	Overall	152	152	152
		Men	83	83	83
		Women	69	69	69
2021	AUROC Mean (SD)	Overall	0.66 (1.70e-03)	0.67 (1.90e-03)	0.65 (3.45e-03)
		Men	0.64 (2.71e-03)	0.66 (2.74e-03)	0.64 (4.42e-03)
		Women	0.67 (1.88e-03)	0.68 (2.19e-03)	0.67 (4.24e-03)
	N negative class	Overall	9712	9712	9712
		Men	4998	4998	4998
		Women	4714	4714	4714
	N positive class	Overall	109	109	109
		Men	62	62	62
		Women	47	47	47
2022	AUROC Mean (SD)	Overall	0.64 (2.47e-03)	0.62 (2.92e-03)	0.65 (5.66e-03)
		Men	0.75 (4.02e-03)	0.7 (4.61e-03)	0.78 (8.00e-03)
		Women	0.57 (3.46e-03)	0.56 (3.52e-03)	0.56 (8.63e-03)
	N negative class	Overall	6511	6511	6511
		Men	3464	3464	3464
		Women	3047	3047	3047
	N positive class	Overall	34	34	34
		Men	19	19	19
		Women	15	15	15

Supplementary Table 14. Predictive Performance (AUROC) by Year of Diabetes Diagnosis for Split 5 (MSSR-Refined).

Diabetes diagnosis year	Metric	Sex	Hybrid	Real	Synthetic
2003	AUROC Mean (SD)	Overall	0.64 (3.99e-04)	0.64 (5.20e-04)	0.64 (7.53e-04)
		Men	0.65 (7.17e-04)	0.65 (6.24e-04)	0.65 (1.23e-03)
		Women	0.62 (6.86e-04)	0.63 (7.25e-04)	0.62 (1.43e-03)
	N negative class	Overall	4544	4544	4544
		Men	2131	2131	2131
		Women	2413	2413	2413
	N positive class	Overall	1474	1474	1474
		Men	765	765	765
		Women	709	709	709

2004	AUROC Mean (SD)	Overall	0.65 (4.80e-04)	0.65 (4.97e-04)	0.65 (7.43e-04)
		Men	0.63 (6.48e-04)	0.63 (6.14e-04)	0.62 (1.19e-03)
		Women	0.68 (6.02e-04)	0.67 (7.68e-04)	0.67 (1.02e-03)
	N negative class	Overall	5390	5390	5390
		Men	2572	2572	2572
		Women	2818	2818	2818
	N positive class	Overall	1425	1425	1425
		Men	739	739	739
		Women	686	686	686
2005	AUROC Mean (SD)	Overall	0.64 (4.51e-04)	0.64 (5.21e-04)	0.64 (8.73e-04)
		Men	0.67 (8.31e-04)	0.67 (7.40e-04)	0.67 (1.65e-03)
		Women	0.62 (6.14e-04)	0.62 (6.36e-04)	0.6 (1.05e-03)
	N negative class	Overall	5166	5166	5166
		Men	2582	2582	2582
		Women	2584	2584	2584
	N positive class	Overall	1330	1330	1330
		Men	718	718	718
		Women	612	612	612
2006	AUROC Mean (SD)	Overall	0.67 (5.16e-04)	0.67 (5.23e-04)	0.66 (9.36e-04)
		Men	0.68 (5.65e-04)	0.68 (5.67e-04)	0.66 (1.24e-03)
		Women	0.67 (9.54e-04)	0.68 (9.00e-04)	0.65 (1.31e-03)
	N negative class	Overall	5682	5682	5682
		Men	2859	2859	2859
		Women	2823	2823	2823
	N positive class	Overall	1252	1252	1252
		Men	705	705	705
		Women	547	547	547
2007	AUROC Mean (SD)	Overall	0.69 (4.10e-04)	0.69 (4.18e-04)	0.67 (6.26e-04)
		Men	0.71 (4.56e-04)	0.71 (4.80e-04)	0.7 (7.13e-04)
		Women	0.66 (7.10e-04)	0.66 (7.39e-04)	0.65 (1.02e-03)
	N negative class	Overall	7016	7016	7016
		Men	3612	3612	3612
		Women	3404	3404	3404
	N positive class	Overall	1460	1460	1460
		Men	791	791	791
		Women	669	669	669
2008	AUROC Mean (SD)	Overall	0.66 (5.13e-04)	0.67 (5.72e-04)	0.65 (7.52e-04)

		Men	0.64 (6.06e-04)	0.64 (6.66e-04)	0.62 (8.36e-04)
		Women	0.68 (5.97e-04)	0.69 (7.26e-04)	0.67 (1.04e-03)
	N negative class	Overall	7361	7361	7361
		Men	3867	3867	3867
		Women	3494	3494	3494
	N positive class	Overall	1292	1292	1292
		Men	702	702	702
		Women	590	590	590
2009	AUROC Mean (SD)	Overall	0.7 (4.26e-04)	0.71 (3.91e-04)	0.68 (7.52e-04)
		Men	0.72 (4.25e-04)	0.73 (4.86e-04)	0.7 (1.19e-03)
		Women	0.67 (7.81e-04)	0.68 (7.40e-04)	0.65 (1.12e-03)
	N negative class	Overall	7414	7414	7414
		Men	4052	4052	4052
		Women	3362	3362	3362
	N positive class	Overall	1136	1136	1136
		Men	658	658	658
		Women	478	478	478
2010	AUROC Mean (SD)	Overall	0.67 (4.48e-04)	0.68 (4.83e-04)	0.66 (6.60e-04)
		Men	0.66 (5.49e-04)	0.66 (6.66e-04)	0.66 (9.32e-04)
		Women	0.68 (9.44e-04)	0.7 (7.81e-04)	0.66 (1.01e-03)
	N negative class	Overall	7663	7663	7663
		Men	4094	4094	4094
		Women	3569	3569	3569
	N positive class	Overall	1100	1100	1100
		Men	641	641	641
		Women	459	459	459
2011	AUROC Mean (SD)	Overall	0.71 (4.63e-04)	0.72 (5.52e-04)	0.68 (7.74e-04)
		Men	0.72 (6.51e-04)	0.73 (6.29e-04)	0.7 (1.06e-03)
		Women	0.7 (7.88e-04)	0.71 (1.04e-03)	0.67 (1.28e-03)
	N negative class	Overall	7444	7444	7444
		Men	4075	4075	4075
		Women	3369	3369	3369
	N positive class	Overall	943	943	943
		Men	511	511	511
		Women	432	432	432
2012	AUROC Mean (SD)	Overall	0.7 (4.67e-04)	0.69 (5.23e-04)	0.69 (8.41e-04)
		Men	0.68 (7.17e-04)	0.67 (6.88e-04)	0.68 (1.12e-03)

		Women	0.73 (7.07e-04)	0.74 (7.84e-04)	0.72 (1.30e-03)
	N negative class	Overall	6730	6730	6730
		Men	3661	3661	3661
		Women	3069	3069	3069
	N positive class	Overall	715	715	715
		Men	432	432	432
		Women	283	283	283
2013	AUROC Mean (SD)	Overall	0.73 (4.56e-04)	0.74 (7.38e-04)	0.72 (7.40e-04)
		Men	0.72 (6.75e-04)	0.73 (7.71e-04)	0.72 (9.50e-04)
		Women	0.74 (8.32e-04)	0.75 (1.33e-03)	0.71 (1.31e-03)
	N negative class	Overall	7103	7103	7103
		Men	3809	3809	3809
		Women	3294	3294	3294
	N positive class	Overall	703	703	703
		Men	421	421	421
		Women	282	282	282
2014	AUROC Mean (SD)	Overall	0.73 (5.92e-04)	0.74 (6.94e-04)	0.71 (9.60e-04)
		Men	0.69 (7.84e-04)	0.68 (9.18e-04)	0.68 (1.50e-03)
		Women	0.78 (9.49e-04)	0.81 (1.23e-03)	0.74 (1.41e-03)
	N negative class	Overall	7011	7011	7011
		Men	3649	3649	3649
		Women	3362	3362	3362
	N positive class	Overall	548	548	548
		Men	307	307	307
		Women	241	241	241
2015	AUROC Mean (SD)	Overall	0.74 (5.69e-04)	0.75 (7.27e-04)	0.7 (1.06e-03)
		Men	0.74 (8.12e-04)	0.74 (8.79e-04)	0.71 (1.46e-03)
		Women	0.74 (9.32e-04)	0.76 (1.05e-03)	0.7 (1.56e-03)
	N negative class	Overall	6457	6457	6457
		Men	3426	3426	3426
		Women	3031	3031	3031
	N positive class	Overall	437	437	437
		Men	246	246	246
		Women	191	191	191
2016	AUROC Mean (SD)	Overall	0.73 (5.98e-04)	0.73 (7.71e-04)	0.72 (1.11e-03)
		Men	0.67 (8.80e-04)	0.67 (9.35e-04)	0.68 (1.54e-03)
		Women	0.81 (9.70e-04)	0.83 (1.31e-03)	0.78 (1.59e-03)

	N negative class	Overall	6561	6561	6561
		Men	3561	3561	3561
		Women	3000	3000	3000
	N positive class	Overall	383	383	383
		Men	205	205	205
		Women	178	178	178
2017	AUROC Mean (SD)	Overall	0.65 (9.20e-04)	0.66 (8.62e-04)	0.61 (2.05e-03)
		Men	0.61 (1.24e-03)	0.62 (1.78e-03)	0.58 (1.63e-03)
		Women	0.69 (1.24e-03)	0.7 (1.44e-03)	0.64 (3.40e-03)
	N negative class	Overall	6820	6820	6820
		Men	3643	3643	3643
		Women	3177	3177	3177
	N positive class	Overall	297	297	297
		Men	162	162	162
		Women	135	135	135
2018	AUROC Mean (SD)	Overall	0.75 (7.05e-04)	0.76 (9.28e-04)	0.73 (1.50e-03)
		Men	0.72 (8.68e-04)	0.73 (1.35e-03)	0.71 (1.81e-03)
		Women	0.8 (1.06e-03)	0.81 (1.28e-03)	0.77 (2.47e-03)
	N negative class	Overall	6951	6951	6951
		Men	3798	3798	3798
		Women	3153	3153	3153
	N positive class	Overall	277	277	277
		Men	156	156	156
		Women	121	121	121
2019	AUROC Mean (SD)	Overall	0.7 (7.59e-04)	0.72 (9.14e-04)	0.65 (1.65e-03)
		Men	0.73 (1.00e-03)	0.73 (9.84e-04)	0.68 (2.02e-03)
		Women	0.66 (1.66e-03)	0.7 (1.66e-03)	0.61 (2.59e-03)
	N negative class	Overall	7318	7318	7318
		Men	4010	4010	4010
		Women	3308	3308	3308
	N positive class	Overall	226	226	226
		Men	125	125	125
		Women	101	101	101
2020	AUROC Mean (SD)	Overall	0.74 (1.05e-03)	0.74 (1.56e-03)	0.71 (1.70e-03)
		Men	0.79 (1.30e-03)	0.79 (1.82e-03)	0.75 (2.48e-03)
		Women	0.7 (1.41e-03)	0.7 (1.91e-03)	0.67 (2.28e-03)
	N negative class	Overall	6464	6464	6464

		Men	3409	3409	3409
		Women	3055	3055	3055
	N positive class	Overall	152	152	152
		Men	83	83	83
		Women	69	69	69
2021	AUROC Mean (SD)	Overall	0.63 (8.67e-04)	0.66 (1.39e-03)	0.59 (1.68e-03)
		Men	0.66 (1.17e-03)	0.68 (1.67e-03)	0.65 (1.80e-03)
		Women	0.57 (1.35e-03)	0.64 (1.95e-03)	0.5 (3.55e-03)
	N negative class	Overall	9712	9712	9712
		Men	4998	4998	4998
		Women	4714	4714	4714
	N positive class	Overall	109	109	109
		Men	62	62	62
		Women	47	47	47
2022	AUROC Mean (SD)	Overall	0.83 (1.72e-03)	0.84 (1.45e-03)	0.77 (5.09e-03)
		Men	0.81 (1.64e-03)	0.81 (1.84e-03)	0.78 (3.98e-03)
		Women	0.89 (5.02e-03)	0.92 (2.42e-03)	0.74 (1.56e-02)
	N negative class	Overall	6511	6511	6511
		Men	3464	3464	3464
		Women	3047	3047	3047
	N positive class	Overall	34	34	34
		Men	19	19	19
		Women	15	15	15

Trajectory-based Predictive performance

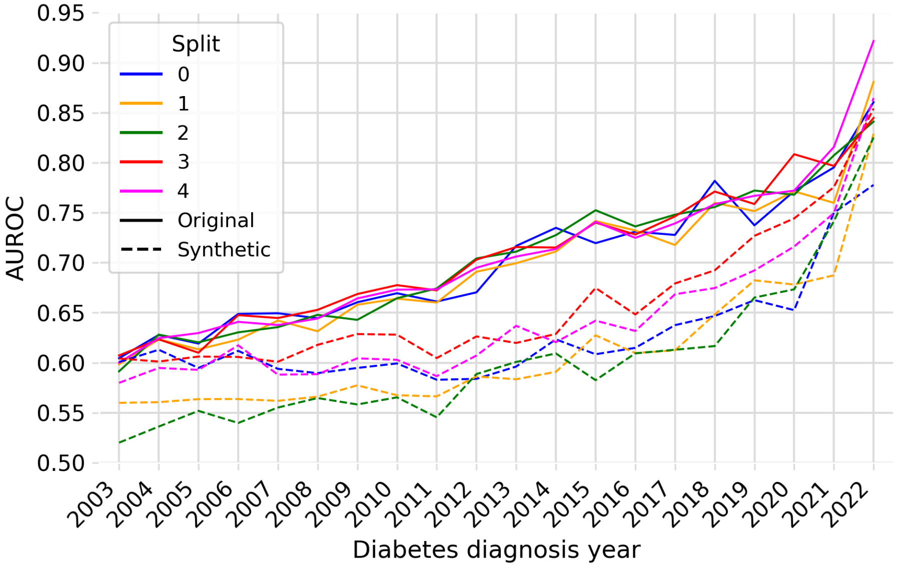

Supplementary Figure 20. Predictive Performance (AUROC) for trajectory-based LSTM models by Year of Diabetes Diagnosis (MSSR-Refined). Comparison of performance for trajectory-based models trained on original (solid) and synthetic (dashed) cohorts across multiple dataset splits. Across splits, the models exhibit similar performance trends with a reduced gap in recent years, indicating that predictive performance for CKD onset is consistent and not affected by initial patient split.

Model Stability

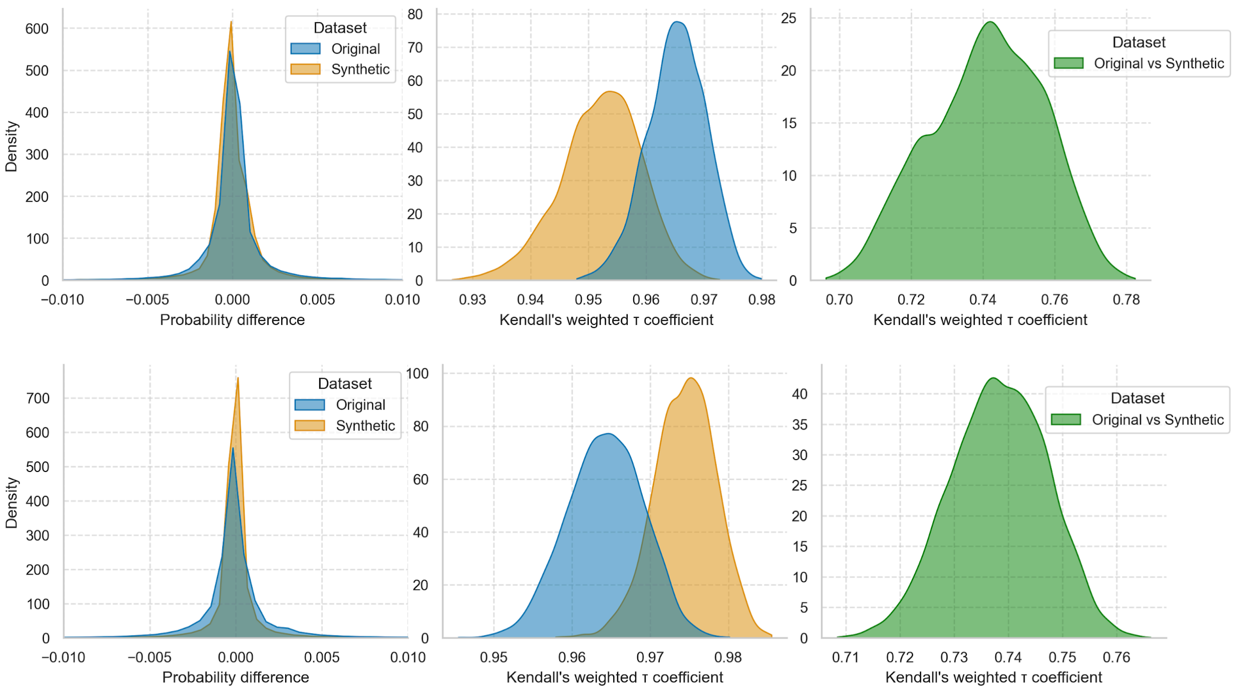

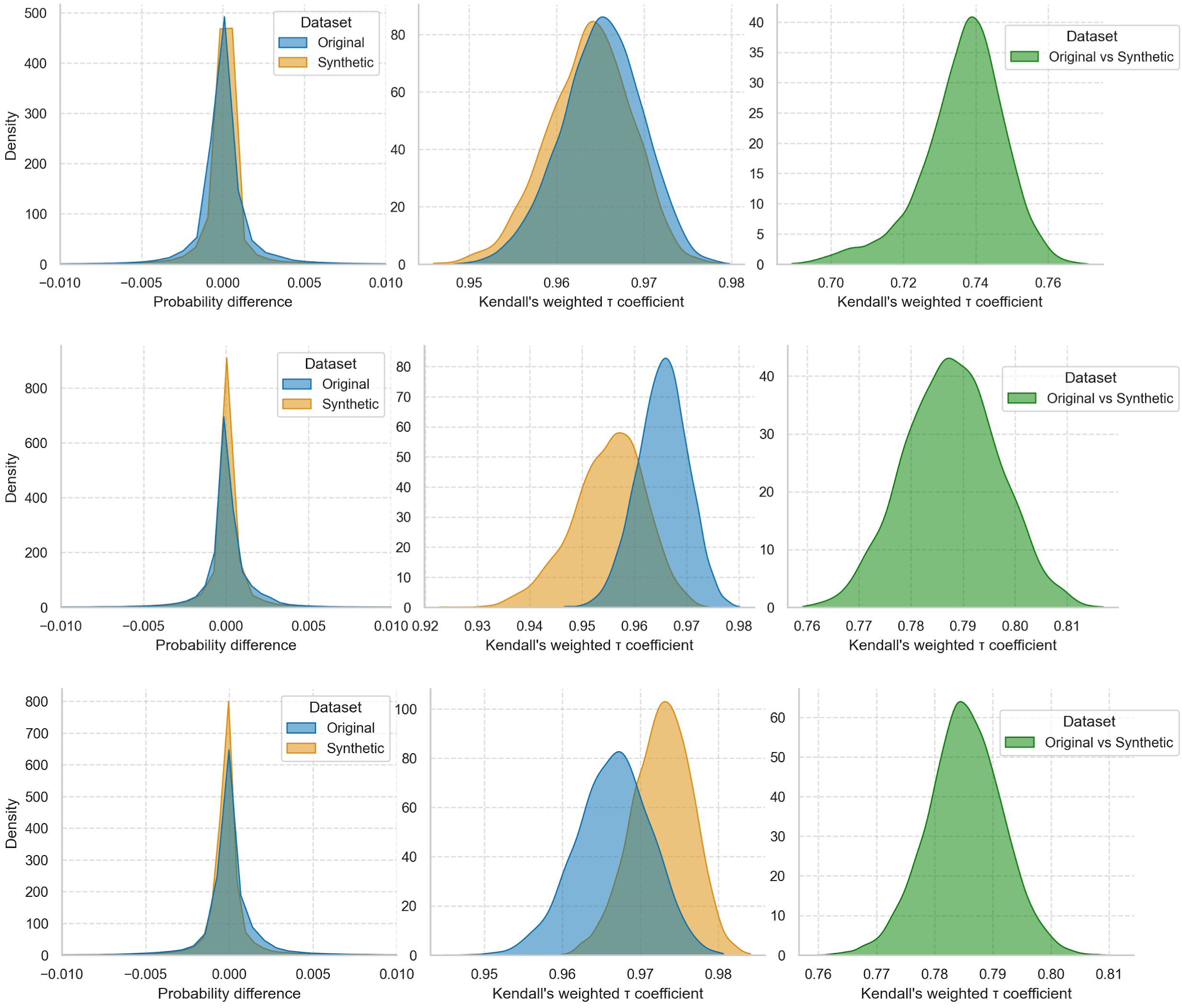

Supplementary Figure 21. Representation stability (MSSR-Refined).

Prediction distribution probabilities, internal feature ranking stability and feature ranking stability between real and synthetic models trained varying dataset splits.

Preprocessing impact

Supplementary Table 15. Percentages of synthetic individuals dropped during preprocessing and refinement steps

Split	Replicate	Percentage no diabetes	Percentage end-point before diabetes	Percentage non-ascending ages	Total percentage dropped
1	0	7.4	2.02	1.3	10.72
2	0	5.28	1.2	0.9	7.38

3	0	9.06	0.8	0.9	10.76
4	0	5.82	1.92	3.13	10.86
5	0	7.77	0.96	0.81	9.54
1	0	7.4	2.02	1.3	10.72
1	1	7.3	2.35	1.03	10.68
1	2	6.27	1.12	2.42	9.8
1	3	12.16	0.83	0.67	13.67
1	4	9.17	1.11	0.98	11.27

Supplementary Table 16. Effect sizes comparing the original and synthetic datasets, quantified using standardized mean differences (Cohen's *d*) for comorbidity prevalence and a multinomial Mahalanobis standardized difference for age at comorbidity diagnosis.

Comorbidity	Effect size of proportions (mean ± SD)	Effect size of age at diagnosis (mean ± SD)
Chronic kidney failure	0.199 ± 0.069	-0.215 ± 0.043
Hepatic steatosis	0.192 ± 0.033	-0.197 ± 0.037
Urinary lithiasis	0.189 ± 0.067	-0.160 ± 0.055
Gout and other crystal arthropathies	0.177 ± 0.048	-0.153 ± 0.039
Asthma	0.176 ± 0.046	-0.158 ± 0.045
Gastroesophageal reflux disease	0.172 ± 0.034	-0.172 ± 0.032
Other arthropathy	0.160 ± 0.028	-0.167 ± 0.018
Osteoarthritis	0.159 ± 0.037	0.158 ± 0.040
Psoriasis	0.152 ± 0.020	-0.152 ± 0.022
Osteoporosis	0.151 ± 0.046	-0.166 ± 0.031
Hypertension	0.149 ± 0.055	0.172 ± 0.047
Tobacco dependence	0.147 ± 0.067	-0.165 ± 0.067
Glaucoma	0.144 ± 0.049	-0.146 ± 0.048
Malabsorption syndrome and food intolerance	0.141 ± 0.026	-0.142 ± 0.015
Fibromyalgia	0.137 ± 0.035	-0.132 ± 0.039
Hypothyroidism	0.136 ± 0.059	-0.135 ± 0.042
Anxiety disorder	0.118 ± 0.060	0.083 ± 0.082
Ischemic heart disease	0.113 ± 0.062	-0.123 ± 0.056
Male	0.111 ± 0.085	-
Female	0.111 ± 0.085	-
Chronic liver disease except cirrhosis	0.109 ± 0.057	-0.117 ± 0.051
Dyslipidemia	0.108 ± 0.086	0.107 ± 0.046

Alcohol dependence	0.107 ± 0.040	-0.118 ± 0.025
Neurological disease with motor deficit not stroke	0.103 ± 0.017	-0.103 ± 0.025
Prostate cancer	0.102 ± 0.027	-0.105 ± 0.023
Retinopathy	0.099 ± 0.035	-0.052 ± 0.076
Obesity	0.099 ± 0.093	-0.100 ± 0.145
Breast cancer	0.094 ± 0.032	-0.110 ± 0.026
COPD (Chronic Obstructive Pulmonary Disease)	0.094 ± 0.073	-0.086 ± 0.083
Limb arteriopathy	0.092 ± 0.033	0.016 ± 0.093
Rheumatoid arthritis and related diseases	0.092 ± 0.020	-0.092 ± 0.014
Colorectal cancer	0.091 ± 0.031	-0.090 ± 0.034
Age-related macular degeneration	0.089 ± 0.025	-0.086 ± 0.023
Schizophrenic disorder	0.089 ± 0.023	-0.090 ± 0.026
Other organic mental disorder	0.081 ± 0.040	-0.073 ± 0.038
Mood disorder	0.080 ± 0.064	-0.104 ± 0.050
Atrial fibrillation	0.076 ± 0.054	-0.033 ± 0.075
Epilepsy	0.072 ± 0.029	-0.064 ± 0.037
Extrapyramidal syndrome	0.072 ± 0.025	-0.066 ± 0.028
Personality and adult behavior disorder	0.072 ± 0.029	-0.070 ± 0.023
Heart failure	0.072 ± 0.089	0.062 ± 0.072
Acquired valvular disease	0.071 ± 0.048	-0.046 ± 0.083
Liver cirrhosis	0.070 ± 0.026	-0.059 ± 0.037
Bladder cancer	0.067 ± 0.032	-0.061 ± 0.027
Regional enteritis and ulcerative colitis	0.066 ± 0.020	-0.058 ± 0.021
Parkinson's disease	0.064 ± 0.028	-0.066 ± 0.032
Collagen disease and vasculitis	0.064 ± 0.032	-0.056 ± 0.026
Occlusion or stenosis of precerebral arteries	0.061 ± 0.028	-0.065 ± 0.027
Intellectual disability	0.056 ± 0.017	-0.054 ± 0.012
Dependence on other substances	0.056 ± 0.028	-0.055 ± 0.028
Aneurysm of aorta	0.056 ± 0.022	-0.056 ± 0.022
Ill-defined and other cerebrovascular disease	0.054 ± 0.037	-0.034 ± 0.044
Sequelae of cerebrovascular disease	0.052 ± 0.028	-0.018 ± 0.065
Diabetes	0.051 ± 0.024	0.051 ± 0.024
Uterine cancer	0.048 ± 0.025	-0.054 ± 0.023

Atopic dermatitis	0.047 ± 0.017	-0.046 ± 0.016
Head and neck cancer	0.044 ± 0.022	-0.036 ± 0.018
Skin melanoma	0.041 ± 0.015	-0.038 ± 0.017
Non-Hodgkin lymphoma	0.041 ± 0.015	-0.033 ± 0.024
Dementia	0.039 ± 0.019	-0.030 ± 0.029
HIV	0.038 ± 0.020	-0.040 ± 0.016
Other functional disorder	0.037 ± 0.022	-0.035 ± 0.018
Thyroid cancer	0.031 ± 0.012	-0.034 ± 0.010
Congenital heart and circulatory anomaly	0.030 ± 0.015	-0.034 ± 0.016
Early childhood adolescence disorder	0.029 ± 0.026	-0.028 ± 0.024
Intraabdominal arteriopathy	0.028 ± 0.016	-0.028 ± 0.018
Kidney and renal pelvis cancer	0.028 ± 0.019	-0.024 ± 0.018
Cervical cancer	0.024 ± 0.010	-0.027 ± 0.008
Leukemia	0.022 ± 0.012	-0.018 ± 0.013
Ovarian cancer	0.020 ± 0.012	-0.023 ± 0.012
Bronchial and lung cancer	0.019 ± 0.014	-0.014 ± 0.017
Pancreatic cancer	0.018 ± 0.014	-0.016 ± 0.012
Bone and soft tissue cancer	0.017 ± 0.010	-0.010 ± 0.017
Hodgkin's disease	0.017 ± 0.014	0.013 ± 0.022
Stomach cancer	0.016 ± 0.010	-0.003 ± 0.017
Kaposi's sarcoma	0.016 ± 0.013	0.014 ± 0.013
Eating disorder	0.014 ± 0.011	-0.006 ± 0.013
Immunoproliferative cancer	0.012 ± 0.011	-0.007 ± 0.011
Other developmental disorder	0.011 ± 0.011	-0.006 ± 0.013
Testicular cancer	0.009 ± 0.006	-0.003 ± 0.008
Autism spectrum disorder	0.009 ± 0.006	0.004 ± 0.012
Liver and biliary tract cancer	0.008 ± 0.010	-0.008 ± 0.007
