# Supplementary material for: High‐Fidelity Synthetic Data Replicates Clinical Prediction Performance in a Million‐Patient Diabetes Cohort
Source: Adv Sci (Weinh). 2026 Mar 16;13(29):e16196. doi: 10.1002/advs.202516196 (PMC13205598; doi:10.1002/advs.202516196)
Supplement: Supplementary file 1 — Supporting File: advs74764‐sup‐0001‐SuppMat.zip. [file ADVS-13-e16196-s001.zip › advs74764-sup-0002-SuppMat.pdf]

Supplementary Methods: High-Fidelity Synthetic Data Replicates Clinical Prediction Performance in a Million-Patient Diabetes Cohort

Francisco Ortuño[1,+,*], Victor de la Oliva Roque[2,3,+], Javier-Ignacio Ramirez-Lopez[1,], David P. Kreil[4], Joaquín Dopazo[2,3,*], Carlos Loucera[2,3,5,*]

1. Department of Computer Engineering, Automation and Robotics, University of Granada, Granada, Spain.

2. Platform for Computational Medicine, Andalusian Public Foundation Progress and Health-FPS, Seville, Spain

3. Institute of Biomedicine of Seville, IBiS, University Hospital Virgen del Rocío/CSIC/University of Sevilla, 41013 Sevilla, Spain

4. Institute of Molecular Biotechnology, Department of Biotechnology and Food Science, BOKU University, Vienna, Austria

5. Department of Computer Science and Artificial Intelligence, Universidad de Sevilla, Avda. Reina Mercedes s/n, 41012, Sevilla, Spain

+: equal contribution

*: corresponding author

- fortuno@ugr.es
- joaquin.dopazo@juntadeandalucia.es
- clou@us.es

Trajectory-based model

Model Architecture

We employed a recurrent neural network based on a Long Short-Term Memory (LSTM) architecture to model temporal dependencies in patient EHRs. The model took as input a three-dimensional tensor of shape (batch size, time steps, number of pathologies). The architecture included the following components:

- Masking layer ignoring padded time steps.
- A single LSTM layer that summarizes the temporal sequence into a fixed-length latent representation.
- A fully connected dense layer with ReLU activation.

- A sigmoid-activated output neuron producing the predicted probability of the target pathology.

The number of LSTM units and the learning rate are treated as hyperparameters and optimized automatically.

Hyperparameter Tuning

The hyperparameter tuning was performed using the Hyperband algorithm, which efficiently explores the search space by allocating computational resources adaptively. The following hyperparameters were considered in this step:

- Number of LSTM units (64 to 128, step size 64)
- Learning rate of the Adam optimizer (sampled logarithmically between 1e-5 and 1e-4)

Hyperparameter selection was based on maximizing the validation Area Under the Receiver Operating Characteristic Curve (AUROC), which is particularly suitable for imbalanced binary classification problems.

Training Procedure

The model training was conducted with a training-validation split of 70%-30% respectively to ensure robust performance estimation. To mitigate class imbalance (positive class represents less than 10% of the overall dataset), the training data was balanced to have an equal number of positive and negative samples (downsampling approach). Training was performed using early stopping based on validation Area Under the ROC Curve (AUROC) and a patience of 5, with restoration of the best-performing weights. Additionally, learning rate reduction on plateau was employed to stabilize convergence. All models were trained using binary cross-entropy loss and optimized with the Adam optimizer.

Model Evaluation

The best-performing model was evaluated on its corresponding validation subset. Performance was assessed using multiple complementary metrics, including:

- Area Under the ROC Curve (AUROC)
- F1-score
- Precision
- Recall

As previously mentioned, AUROC metric was considered our primary metric. Global performance statistics were then computed from predicted probabilities to generate evaluation plots.

Final Model

Training: After tuning, a final model was trained using the optimal hyperparameters identified during the tuning process. This model was trained on the full training dataset (80%) and validated on a held-out validation set (20%). The resulting model was saved for subsequent testing step with the original independent test cohort.

Testing: An independent test cohort, which was not used during training or hyperparameter tuning, was considered. From the test cohort, samples containing invalid structures in either inputs or outputs were excluded from evaluation. Also, those inputs where the endpoint was occurring before diabetes were also discarded. Performance on the test set was quantified using AUROC.

Implementation and Reproducibility

All experiments were implemented using TensorFlow and Keras libraries in Python. The complete experimental pipeline, including data preprocessing, model training, evaluation and figure generation, is fully automated and reproducible. Metrics and visualizations are generated programmatically and stored for subsequent analysis and reporting.
